# Metabolomic and lipidomic changes in heat-stressed chickpea seeds

**DOI:** 10.3389/fpls.2025.1668751

**Published:** 2025-10-13

**Authors:** Uday Chand Jha, Marilyn L. Warburton, Harsh Nayyar, Kadambot H. M. Siddique, P. V. Vara Prasad

**Affiliations:** ^1^ Indian Council for Agricultural Research (ICAR) - Indian Institute of Pulses Research (IIPR), Kanpur, Uttar Pradesh, India; ^2^ Department of Agronomy, Kansas State University, Manhattan, KS, United States; ^3^ United States Department of Agriculture – Agricultural Research Service, Western Regional Plant Introduction Station, Pullman, WA, United States; ^4^ Department of Botany, Panjab University, Chandigarh, India; ^5^ The UWA Institute of Agriculture, The University of Western Australia, Crawley, Perth, WA, Australia

**Keywords:** chickpea, metabolomics, lipidome, heat stress, climate change

## Abstract

Extreme climate induced heat stress during the reproductive phase significantly reduces yield and seed quality in chickpea, a vital cool-season pulse crop. While chickpea plants deploy various biochemical and molecular mechanisms, including the production of protective compounds and heat shock proteins to cope with heat stress, the metabolomic and lipidomic bases of heat tolerance remain poorly understood. This study used untargeted metabolomics and lipidomics to identify key metabolites, lipids, and potential biomarkers in seeds of a heat-tolerant (PI518255) and a heat-sensitive (PI598080) chickpea genotypes exposed to heat stress (35 °C day/20 °C night) under controlled environments. Results from volcano plot analysis revealed that 65 metabolites and 131 lipids were upregulated, while 17 metabolites and 195 lipids were downregulated under heat stress. Heatmap analysis showed that the heat-tolerant genotype had elevated metabolites (Naringenin, Astilbin,1-O-Cinnamoyl-(6-arabinosylglucose), Hesperetin 7-glucoside, luteolin, and neoandrographolide) and lipids [dimethylphosphatidylethanolamine (dMePE), phosphatidylinositol phosphates (PIP), phosphatidylethanolamine (PE), phosphatidylcholines (PC), phosphatidylglycerol (PG), phosphatidylinositol (PI), diacylglycerol monogalactoside (DGMG) (36:5), monogalactosyldiacylglycerol (MGDG), phosphatidic acid (PA), phosphatidylmonomethylethanolamine (PMe), Biotinyl Phosphatidylethanolamine (BiotinylPE), (O-acyl)-omega-hydroxy fatty acids (OAHFAs)], which may serve as diagnostic biomarkers for heat tolerance. Pathway enrichment analysis (KEGG) identified several heat stress-responsive metabolic pathways, including the pentose phosphate pathway, pyruvate metabolism, citrate (TCA) cycle, glyoxylate and dicarboxylate metabolism, starch and sucrose metabolism, glycolysis/gluconeogenesis, and cysteine and methionine metabolism. Lipid metabolic pathways involving MGDG, glycerophosphocholine, PI, PA, PC, phosphatidylcholines, lysophosphatidylcholine (LPC), lysophosphatidylglycerol (LPG), glycerophosphoinositol, and phosphoglyceric acid were also significantly affected. Future research employing targeted metabolomics and lipidomics profiling could elucidate candidate markers to enhance seed yield and quality, and support breeding programs to develop heat- and climate- resilient chickpea cultivars.

## Introduction

Considering the escalating climate variability and occurrence of extreme events, heat stress is emerging as a major constraint to productivity of crops (Firmansyah and Argosubekti, 2020). Climate projections suggest more frequent and intense heat waves (Han et al., 2022), posing a significant threat to food production (Kompas et al., 2024). Heat stress is known to decrease yields of cereal grains and legume crops (Prasad et al., 2017; Sher et al., 2024; Siddique et al., 2025). Chickpea (*Cicer arietinum* L.) is a critical source of plant-based protein and essential micronutrients, contributing to global food and nutrition security (Jha et al., 2024a) and vulnerable to heat stress (Jha et al., 2025a). India is the world’s leading producer (12.3 million tons, about 75% of global production in 2023; FAOSTAT, 2023) of chickpeas. Heat stress adversely affects cellular metabolism across all stages of chickpea development from germination to reproduction and maturity (Jha et al., 2014; Bhandari et al., 2020; Devi et al., 2022, 2023). The crop is particularly vulnerable during the flowering and seed-filling stages, where above optimum temperatures can drastically reduce seed yield and quality (Bhandari et al., 2020; Devi et al., 2023; Jha et al., 2024b; 2025b).Rising temperatures and extreme events have increased heat exposure during the reproductive phase of chickpea (Devasirvatham et al., 2012). Heat stress during reproductive phase impairs pollen viability, stigmatic function, fertilization, pod development, and seed filling (Rani et al., 2020; Bhandari et al., 2020). In turn, this leads to substantial losses in both yield and seed quality (Awasthi et al., 2024).

In addition to yield, heat stress alters the biochemical composition of seeds in many crops (Kumar et al., 2023a; Jha et al., 2024b). While the impact of heat on seed metabolites has been extensively studied in cereal crops (Kumar et al., 2023a; Han et al., 2025), there is limited research on the effects of heat stress on seed quality traits including metabolites and lipids in legumes, and particularly in chickpea. A few studies have reported stress-induced changes in amino acids, carbohydrates, lipids, proteins, and secondary metabolites in legumes such as soybean (*Glycine max* L. Merr., Das et al., 2017), grass pea (*Lathyrus sativus*, Aloui et al., 2023), lentil (*Lens culinaris* L., Sehgal et al., 2019), mung bean (*Vigna radiata* L. R. Wilczek, Priya et al., 2023; Jha et al., 2025c), and chickpea (Devi et al., 2023; Malviya et al., 2025; Ruan et al., 2025).

Plants, including chickpea, activate various biochemical and molecular defense strategies under heat stress, such as producing heat shock proteins and protective metabolites (Al-Whaibi, 2011; Hasanuzzaman et al., 2013). Advances in conventional breeding and genomics have enabled the identification of heat-tolerant genotypes and the discovery of important quantitative trait loci (QTLs) and genes associated with heat tolerance (Jha et al., 2021a, 2021; Kumar et al., 2023b; Mohanty et al., 2025). Functional genomics has further facilitated the identification of candidate genes with putative roles in the stress response (Mohanty et al., 2024). The emerging disciplines of metabolomics and lipidomics offer promising tools to explore the complex biochemical responses to abiotic stress. Metabolomics has already provided valuable insights into drought-responsive metabolic pathways in chickpea (Chaturvedi et al., 2024), while lipidomics is beginning to shed light on stress-induced lipid remodeling in plants (Higashi and Saito, 2019; Qian et al., 2023). However, the investigation and integration of metabolomics and lipidomics to study chickpea’s response to heat stress remains limited.

To address this gap, we conducted untargeted metabolomic and lipidomic profiling of seeds from two chickpea genotypes, one heat tolerant (PI518255) and a heat-sensitive (PI598080), under non-stress and heat stress conditions in controlled environments to identify metabolites and lipids associated with heat tolerance. The biomarkers identified here could serve as potential tools for screening heat tolerance in breeding programs and improving seed quality under rising temperature scenarios.

## Materials and methods

Two contrasting chickpea genotypes, PI518255 (heat-tolerant) (Kaloki et al., 2019; Patre et al., 2023) and PI598080 (heat-sensitive) (Wang et al., 2006) were grown in a greenhouse under a 25°C (day)/15°C (night) controlled temperature regime for 60 days until flower initiation and then transferred to a growth chamber and subjected to 25°C/15°C (non stress) and heat stress regime of 35°C (day)/20°C (night). Seeds were sown in 20 cm diameter pots filled with potting soil (Fafard^®^3B Mix/Metro-Mix^®^830, SUNGRO Horticulture, Agawam, MA, USA). Each genotype was grown with three biological replicates, and each biological replicate included two individual plants. Seeds were harvested at maturity, with three seeds selected randomly from each plant for downstream analysis.

The growth chamber provided photosynthetically active radiation (400–700 nm) at 600 μmol m^-^² s^-^¹ using cool fluorescent lamps, with a 12 h photoperiod. Relative humidity averaged 60% (Jha et al., 2025c). Plants were watered regularly to maintain field capacity (saturated soil without runoff) and received nutrient supplementation every 7–14 days using 1/2 teaspoon of Miracle-Gro (24-8-16) per 4.5 L of water (Jha et al., 2025c). Temperature data were recorded using a HOBO^®^ data logger (Onset Computer Corporation, USA) and are presented in **Supplementary Figure S1**.

### Sample preparation for metabolite isolation

Physiologically matured seed samples were thawed on ice. A 100 mg sample was weighed and transferred into a tube. Then, 800 µL of 80% methanol was added. All samples were grounded on a grinder at 65 Hz for 180 seconds, vortexed, and sonicated for 30 minutes at 4°C.

Each sample was then kept at -20°C for 1 hour, vortexed for 30 seconds, and kept at 4°C for 30 minutes. The samples were then centrifuged at 12,000 rpm and 4°C for 15 minutes. The supernatant was transferred to a new tube and kept at -20°C for 1 hour, then centrifuged again at 12,000 rpm and 4°C for 15 minutes. Finally, 200 µL of the supernatant and 5 µL of DL-o-Chlorophenylalanine (0.14 mg/mL) were transferred to a vial for LC-MS analysis (Chianese et al., 2022; Wang et al., 2024).

Metabolite separation was performed using an ACQUITY ultra-performance liquid chromatography (UPLC) (Waters, Milford, MA, USA) system coupled with a Q Exactive MS (Thermo), with screening by electrospray ionization-mass spectrometry (ESI-MS). The liquid chromatography (LC) system, equipped with an ACQUITY UPLC HSS T3 column (100 × 2.1 mm, 1.8 µm), used a mobile phase of Solvent A (0.05% formic acid in water) and Solvent B (acetonitrile). The gradient elution was as follows: 0–1 min, 5% B; 1–12 min, 5%–95% B; 12–13.5 min, 95% B; 13.5–13.6 min, 95%–5% B; and 13.6–16 min, 5% B. The flow rate was 0.3 mL/min, the column temperature was 40°C, and the sample manager temperature was 4°C.

Mass spectrometry was conducted in both positive (ESI+) and negative (ESI–) electrospray ionization modes with the following settings: ESI+ mode—heater temperature (300°C), sheath gas flow rate (45 arb), auxiliary gas flow rate (15 arb), sweep gas flow rate (1 arb), spray voltage (3.0 kV), capillary temperature (350°C), and S-Lens RF level (30%); ESI–mode—heater temperature (300°C), sheath gas flow rate (45 arb), auxiliary gas flow rate (15arb), sweep gas flow rate (1 arb), spray voltage (3.2 kV), capillary temperature (350°C), and S-Lens RF level (60%) (Thermo Scientific Q Exactive™ Orbitrap MS).

### Sample preparation for lipid isolation

Samples were thawed on ice. Fifty milligrams of each sample was weighed into a tube, and 1.5 mL of chloroform: methanol (2:1, v/v) solution was added. The mixture was then vortexed for 1 minute and ground for 180 seconds at 65 Hz. Following this, 0.5 mL of ultrapure water was added, and the samples were sonicated for 30 minutes at 4°C. The samples were then centrifuged for 10 minutes at 3,000 rpm and 4°C. The lower phase was carefully transferred to a new tube and dried under a stream of nitrogen. The dried extract was resuspended in 200 µL of an isopropyl alcohol: methanol (1:1, v/v) solution. Five microliters of lysophosphatidylcholine (12:0) (0.14 mg/mL) was added as an internal standard. Finally, the solution was centrifuged for 10 minutes at 12,000 rpm and 4°C, and the resulting supernatant was transferred for LC-MS analysis (Chianese et al., 2022; Wang et al., 2024).

Lipid separation was carried out using UPLC coupled with a Q Exactive mass spectrometer (Thermo Fisher Scientific). The UPLC system was equipped with an ACQUITY UPLC BEH C18 column (100 × 2.1 mm × 1.7 μm particle size; Waters, Milford, MA, USA). The mobile phases comprised solvent A (60% acetonitrile, 40% water, + 10 mM ammonium formate) and solvent B (10% acetonitrile, 90% isopropyl alcohol, 10 mM ammonium formate).The elution gradient was as follows: 0–1.0 min, 30% B; 1.0–10.5 min, gradient from 30–100% B; 10.5–12.5 min, 100% B; 12.5–12.51 min, gradient from 100–30% B; 12.51–16 min, 30% B. The flow rate was 0.3 mL/min, the column temperature was maintained at 40°C, and the auto sampler was kept at 4°C (Chianese et al., 2022; Wang et al., 2024).

Mass spectrometry was conducted in both positive (ESI+) and negative (ESI–) electrospray ionization modes with the following settings: ESI+ mode—heater temperature (300°C), sheath gas flow rate (45 arb), auxiliary gas flow rate (15 arb), sweep gas flow rate (1 arb), spray voltage (3.0 kV), capillary temperature (350°C), and S-Lens RF level (30%); ESI–mode—heater temperature (300°C), sheath gas flow rate (45 arb), auxiliary gas flow rate (15arb), sweep gas flow rate (1 arb), spray voltage (3.2 kV),capillary temperature (350°C), and S-Lens RF level (60%) (Thermo Scientific Q Exactive™ Orbitrap MS).

### Statistical analysis

For metabolites, the raw data were acquired and aligned using the Compound Discoverer (3.0, Thermo) based on the m/z value and the retention time of the ion signals. Ions from both ESI- and ESI+ were merged and imported into the SIMCA-P program (version 14.1) for multivariate analysis. For lipid, raw LC-MS data were acquired and processed using LipidSearch software (Thermo Fisher Scientific), with ion alignment based on mass-to-charge ratio (m/z) and retention time. Ion data from ESI+ and ESI– modes were merged and imported into SIMCA-P (version 14.1) for multivariate statistical analysis. Principal components analysis (PCA) was used as an unsupervised method to visualize data distribution and identify potential outliers (Wold et al., 1987). Supervised regression modeling was then applied using Partial Least Squares Discriminant Analysis (PLS-DA) (Barker and Rayens, 2003) and Orthogonal Partial Least Squares Discriminant Analysis (OPLS-DA) to identify potential biomarkers. These biomarkers were then filtered and confirmed based on Variable Importance in Projection (VIP> 1.5) scores (Wold et al., 1993), fold-change thresholds (|log_2_FC|>1) {where FC=Average abundance in PI518255 Group under non stress/Average abundance in PI598080 Group under non stress} and FC=Average abundance in PI518255 Group under heat stress/Average abundance in PI598080 Group under heat stress}. Metabolites and lipids with log2 fold change of ≥ 1 (up-regulated) and ≤ -1 (down-regulated) and P-value < 0.05 (statistical significance) were considered as differentially expressed.

### Identification of potential biomarkers

Key metabolites were identified by matching accurate mass and MS/MS fragmentation data against publicly available online databases, including the Human Metabolome Database (www.hmdb.ca), Chemspider (www.chemspider.com), and Mass Bank (www.massbank.jp). Raw LC-MS data were processed using LipidSearch software (Thermo Fisher Scientific) for lipid biomarker identification, with alignment based on m/z ratios and retention time. Where necessary, identities were further confirmed using authentic standards by comparing retention times and MS/MS fragmentation patterns with those of the candidate compounds.

### Cluster analysis

Hierarchical clustering analysis (HCA) was performed using the complete linkage algorithm implemented in Cluster 3.0 (Stanford University).The results were visualized using the PheatmapR package (version 1.0.12, RaivoKolde). The HCA was based on metabolite ratios from two independent experiments using significantly altered metabolites. In the heatmaps, color intensity represents the degree of metabolite change, with red indicating increased levels and green indicating decreased levels relative to the mean.

### Correlation network of metabolites

A correlation network was constructed using pathway and annotation data from the KEGG database (Kanehisa and Goto, 2000) and the MetaboAnalyst platform to explore the functional relationships among significantly altered metabolites. All significant metabolites (P < 0.05, as determined by MetaboAnalyst) were subjected to categorical annotation using the HMDB and KEGG databases to identify associated metabolic pathways, enzyme interactions, and related biological functions. Enriched pathways were visualized using dot plots and network diagrams, offering insights into the interconnected roles of key biomarkers in response to heat stress.

## Results

Under non-stress condition (25°C day/15°C night), PI518255 had effective pods/plant (57) and seeds per plant (60) and PI598080 had (50 and 52, respectively). The other morpho-physiological trait values are given in **Supplementary Table S1**.

**Figure 1:**
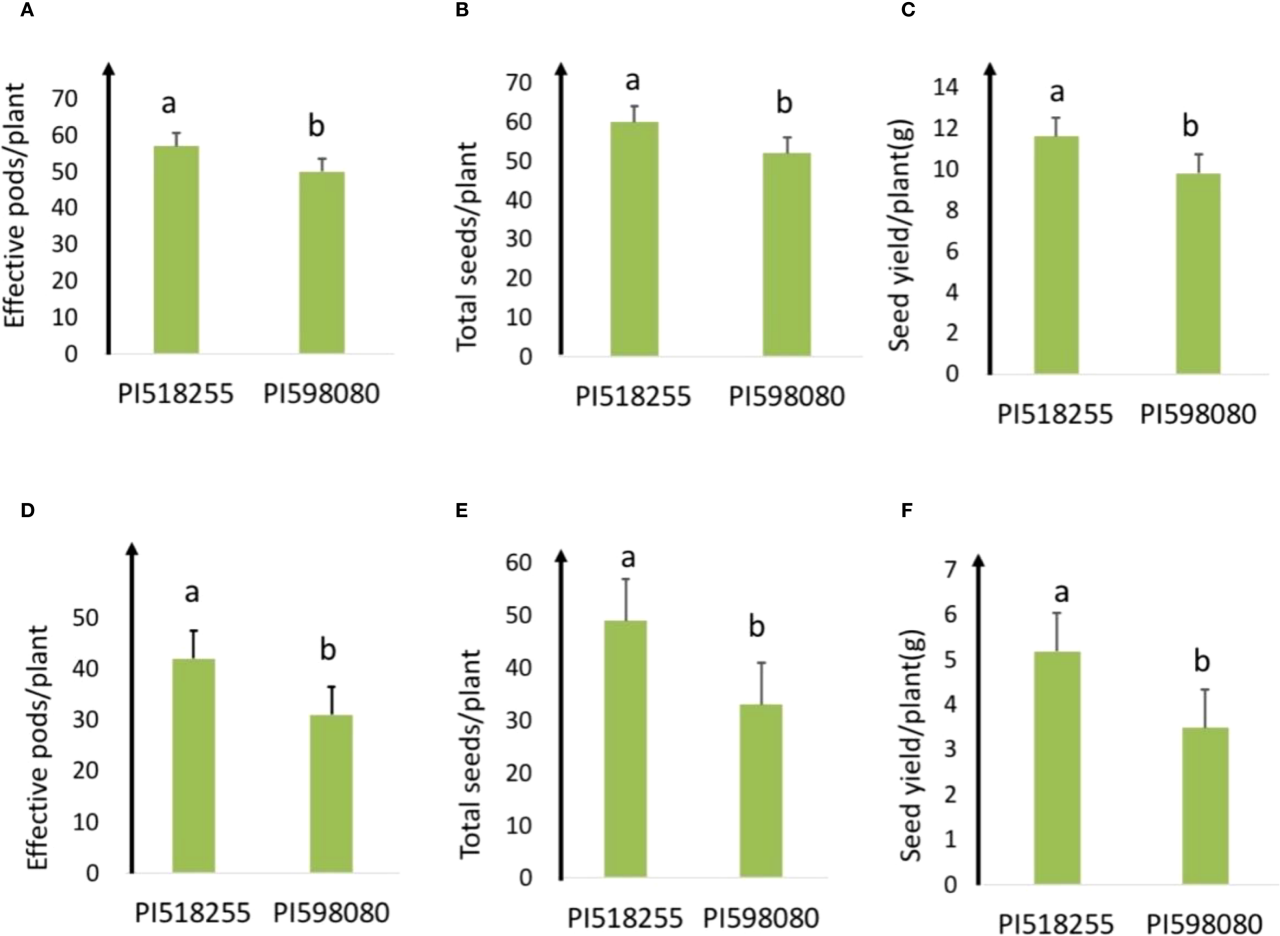
**(A)** Effective pods/plant; **(B)** total seeds/plant; and **(C)** seed yield/plant of PI 518255 and PI 598080 chickpea genotypes at 25°/15 °C(non-stress) and **(D)** effective pods/plant; **(E)** total seed/plant; and **(F)** seed yield/plant of PI 518255 and PI 598080 chickpea genotypes at 35°/20 °C under heat stress (HS) conditions. LSD (least significant difference) values (*P* < 0.05); Values are means + SE (standard error). (*n* = 5).

Under heat stress (35°C day/20°C night), the heat-tolerant genotype PI518255 produced more effective pods per plant (42) and seeds per plant (49) than the heat-sensitive genotype PI598080 (31 and 33, respectively). Correspondingly, PI518255 produced significantly higher seed yield per plant (5.2g) than PI598080 (3.5g), confirming its greater tolerance to heat stress (**Figure 1**).

### Multivariate statistical analysis

**Figure 2:**
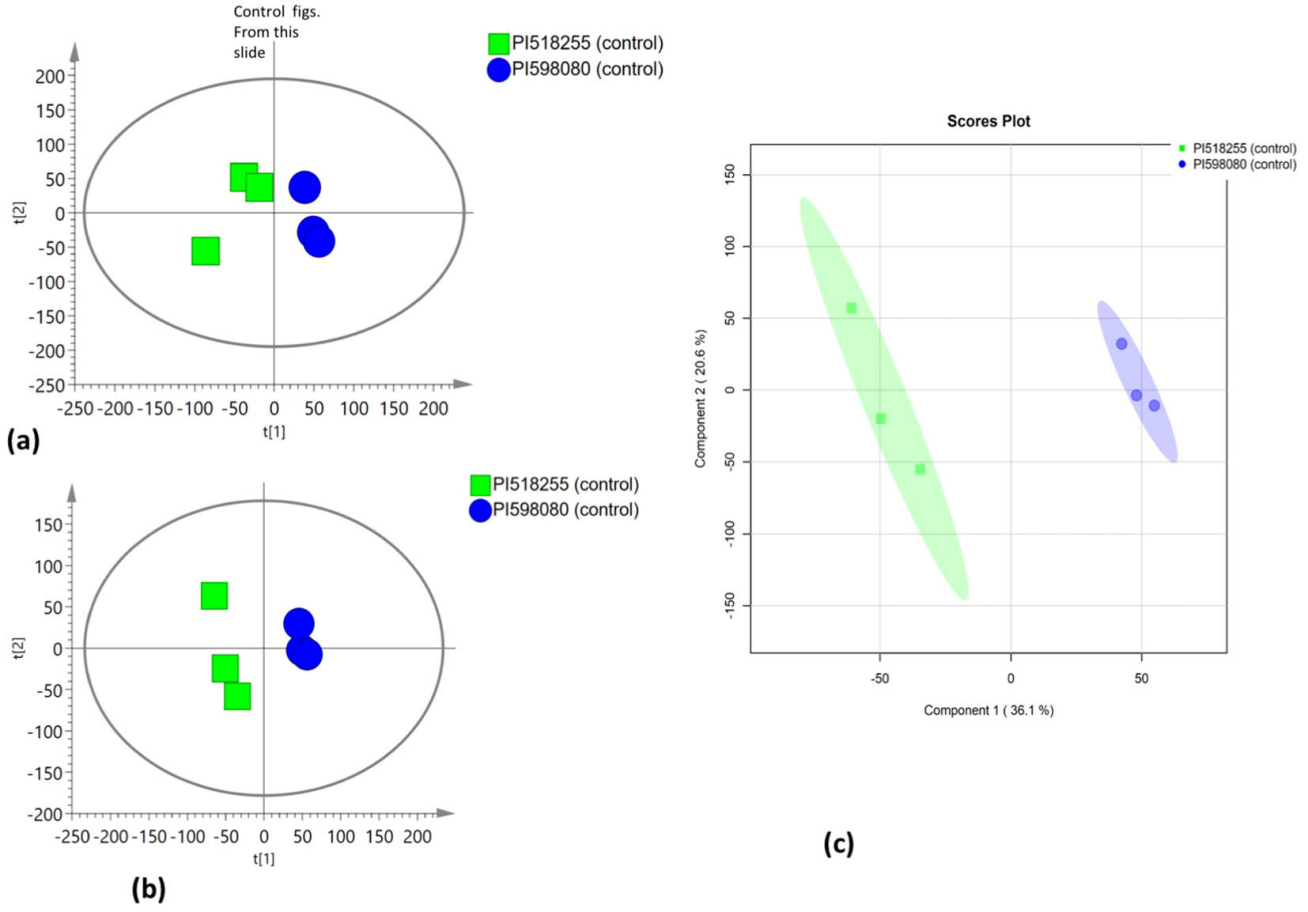
**(A)** The scores scatter plot of PCA model for the identified metabolites under non stress; **(B)** The scores scatter plot of PLSDA model for the identified metabolites under non stress; and **(C)** The scores scatter plot of OPLS-DA model for the identified metabolites under non stress.

**Figure 3:**
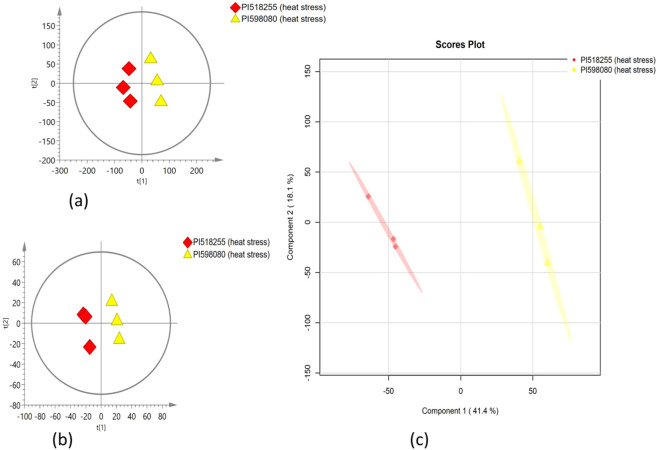
**(A)** The scores scatter plot of PCA model for the identified metabolites under heat stress; **(B)** The scores scatter plot of PLSDA model for the identified metabolites under heat stress; and **(C)** The scores scatter plot of OPLS-DA model for the identified metabolites under heat stress.

**Figure 4:**
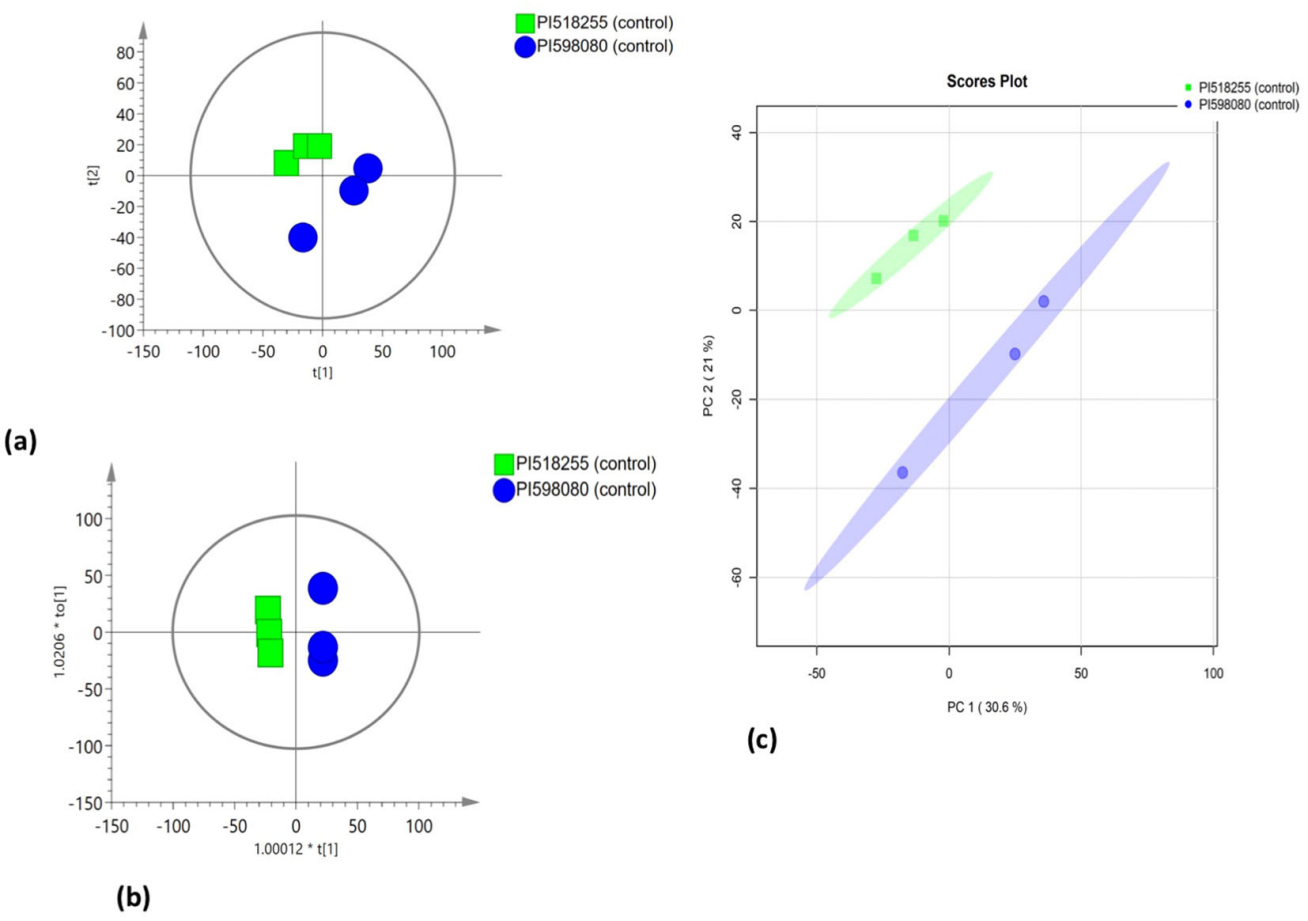
The scores scatter plots of **(A)** PCA model for the identified lipids under non-stress; **(B)** PLSDA model for the identified lipids under heat stress; and **(C)** OPLS-DA model for the identified lipids under non-stress.

**Figure 5:**
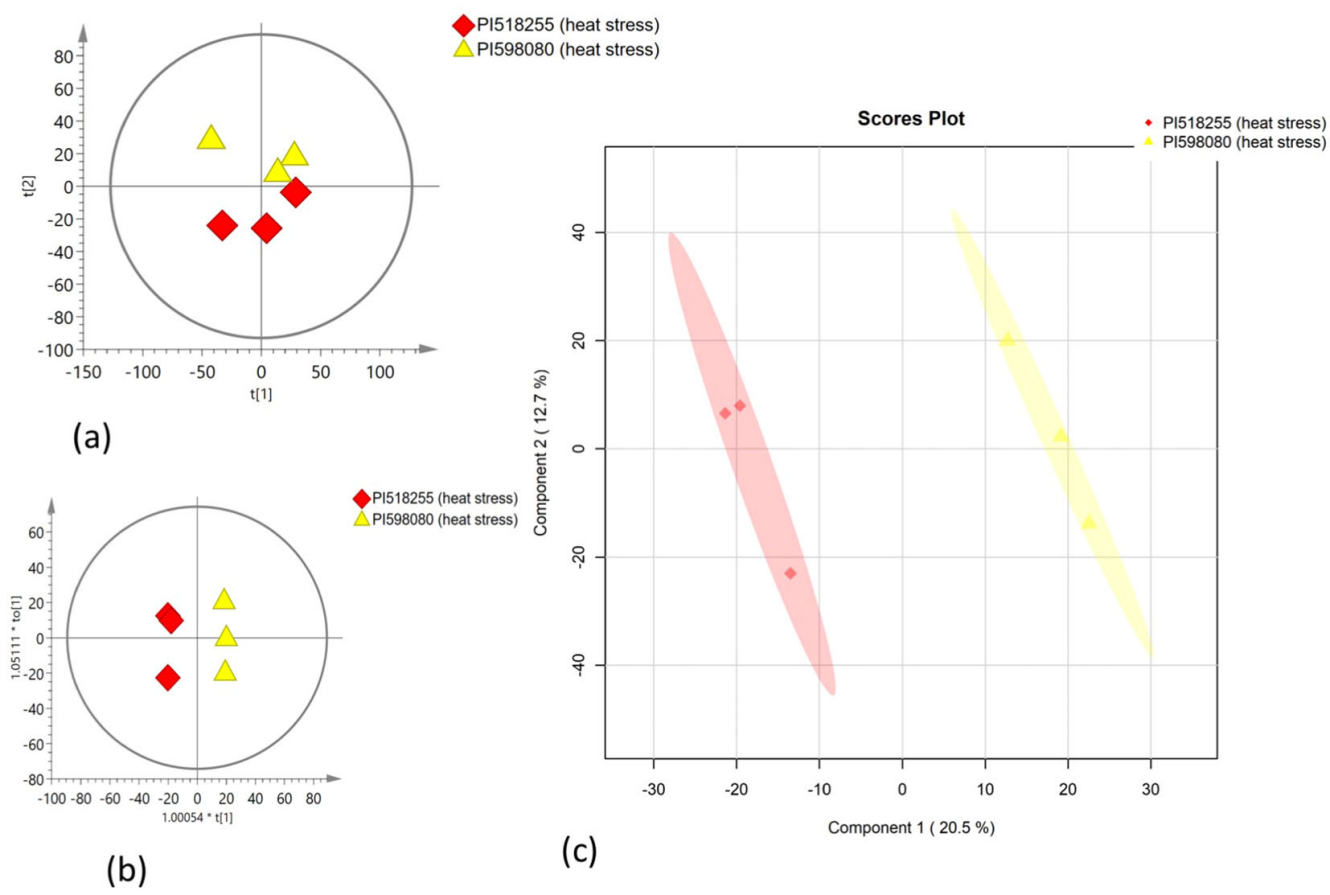
The scores scatter plots of **(A)** PCA model for the identified lipids under heat stress; **(B)** PLSDA model for the identified lipids under heat stress; and **(C)** OPLS-DA model for the identified lipids under heat stress.

**Figure 6:**
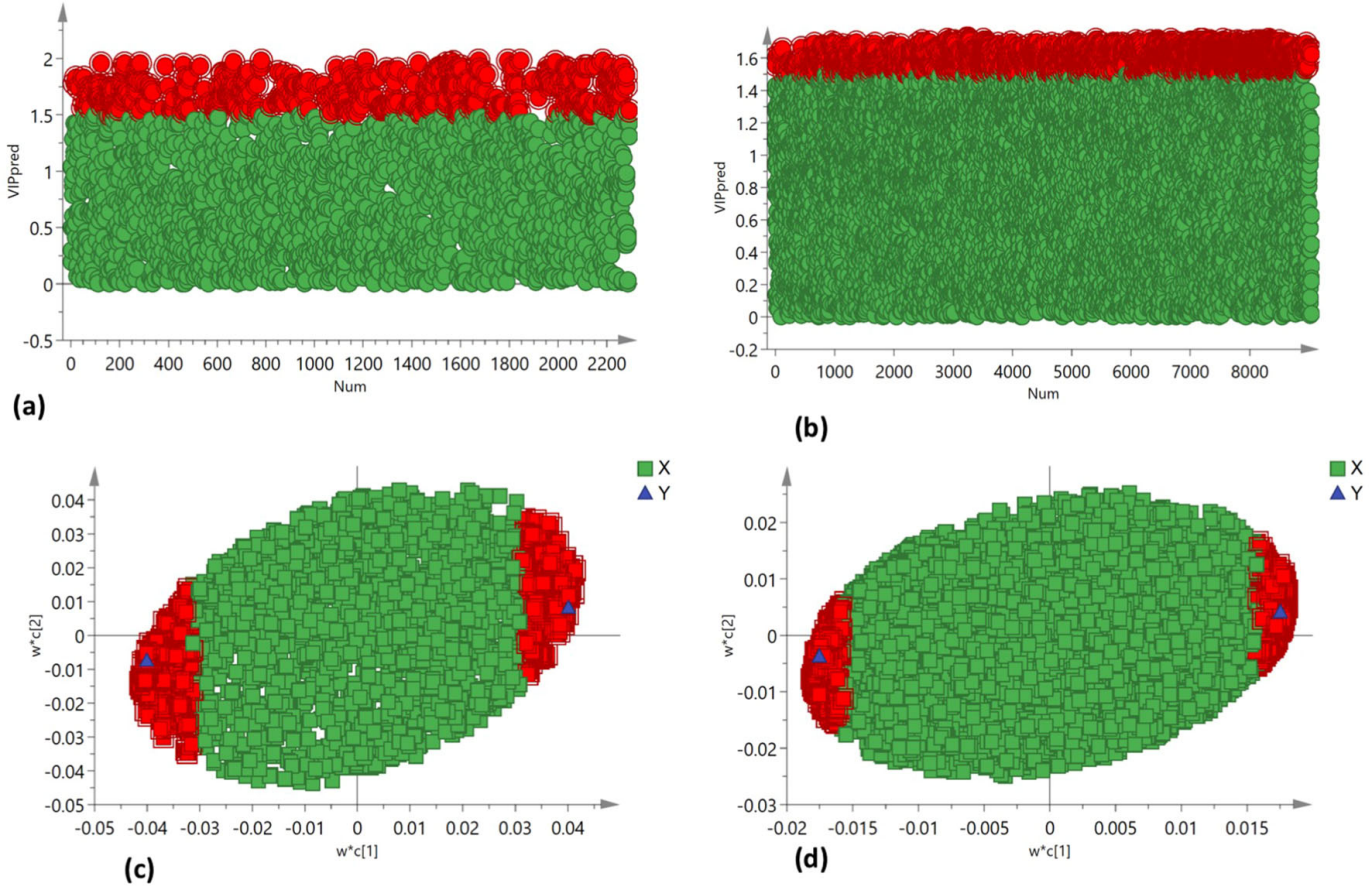
The distribution of **(A)** VIP values (VIP > 1.5) for the metabolites under non-stress; **(B)** VIP values (VIP > 1.5) for the lipids under non stress; loading plot of **(C)** PLS-DA model, the metabolites with red box were labeled as significant compounds (VIP > 1.5); and **(D)** PLS-DA model, the lipids with red box were labeled as significant compounds (VIP > 1.5).

**Figure 7:**
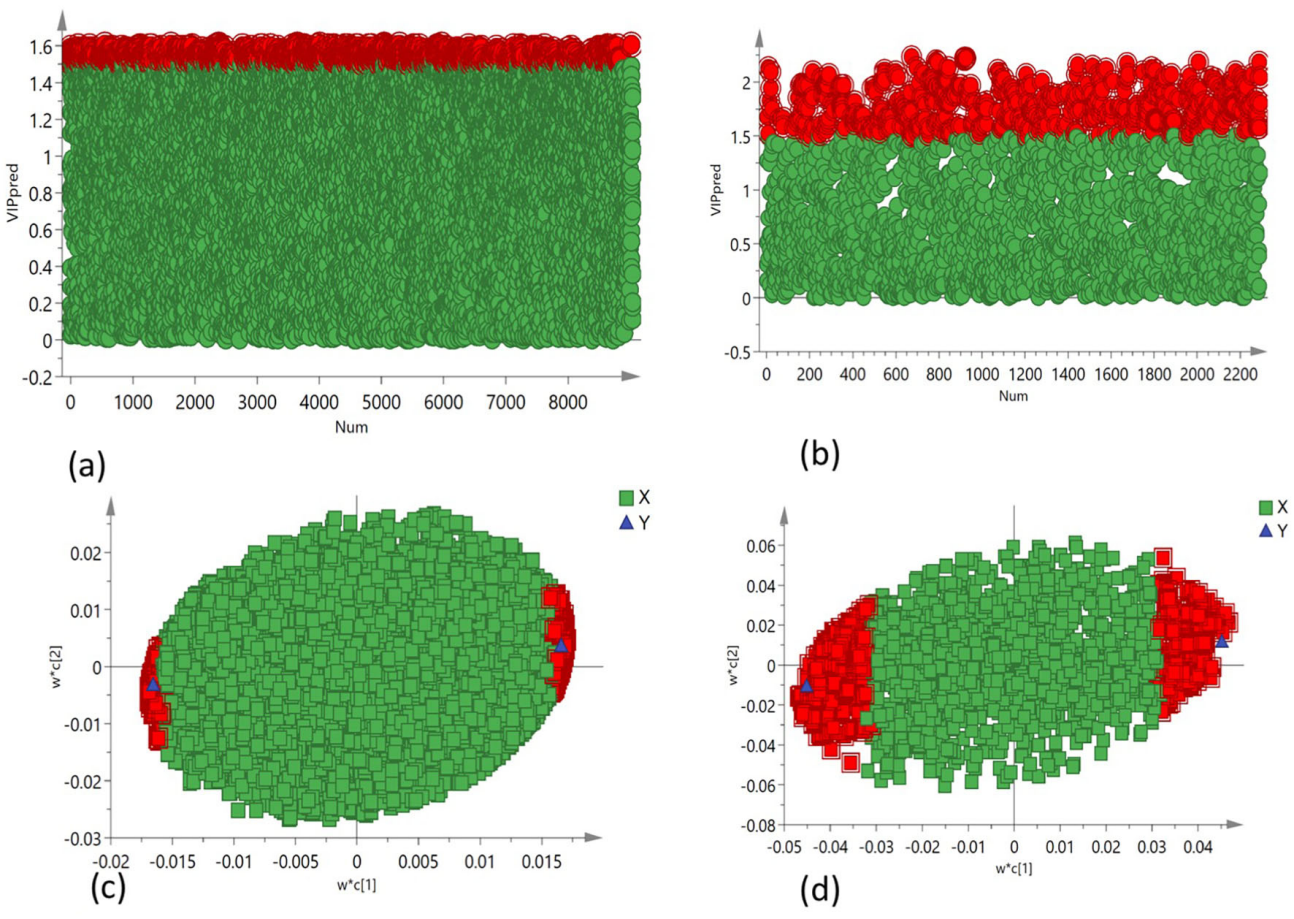
The distribution of **(A)** VIP values (VIP > 1.5) for the metabolites under heat stress; **(B)** VIP values (VIP > 1.5) for the lipids under heat stress; loading plot of **(C)** PLS-DA model, the metabolites with red box were labeled as significant compounds (VIP > 1.5); and **(D)** PLS-DA model, the lipids with red box were labeled as significant compounds (VIP > 1.5).

PCA was employed to assess the global variation in metabolomic and lipidomic datasets. The PCA score plots [**Figure 2A** (under non stress) and **Figure 3A** (under heat stress) for metabolites; **Figure 4A** (under non stress) and **Figure 5A** (under heat stress) for lipids] displayed clear grouping trends, reflecting separation between the two genotypes. To further refine group differences and minimize non-specific effects, supervised classification methods PLS-DA [**Figure 2B** (under non stress) and **Figure 3B** (under heat stress) for metabolites, **Figure 4B** (under non stress) and **Figure 5B** (under heat stress) for lipids] and OPLS-DA [**Figure 2C** (under non stress) and **Figure 3C** (under heat stress) for metabolites, **Figure 4C** (under non stress) and **Figure 5C** (under heat stress) for lipids] were applied. Both models revealed distinct separation between the genotypes under non stress and heat stress. Significantly altered metabolites and lipids were filtered using VIP values (VIP > 1.5). The distributions of both PI518255 and PI598080 are shown in **Figure 6A** (metabolites), **Figure 6B** (lipids) under non-stress, and **Figures 7A** (metabolites), **Figures 7B** (lipids) under heat stress conditions. Corresponding loading plots (**Figures 6C, D**) under non-stress and (**Figures 7C, D**) under heat stress highlight the most influential features, with red boxes indicating key discriminative metabolites and lipids.

### Single variable analysis

**Figure 8:**
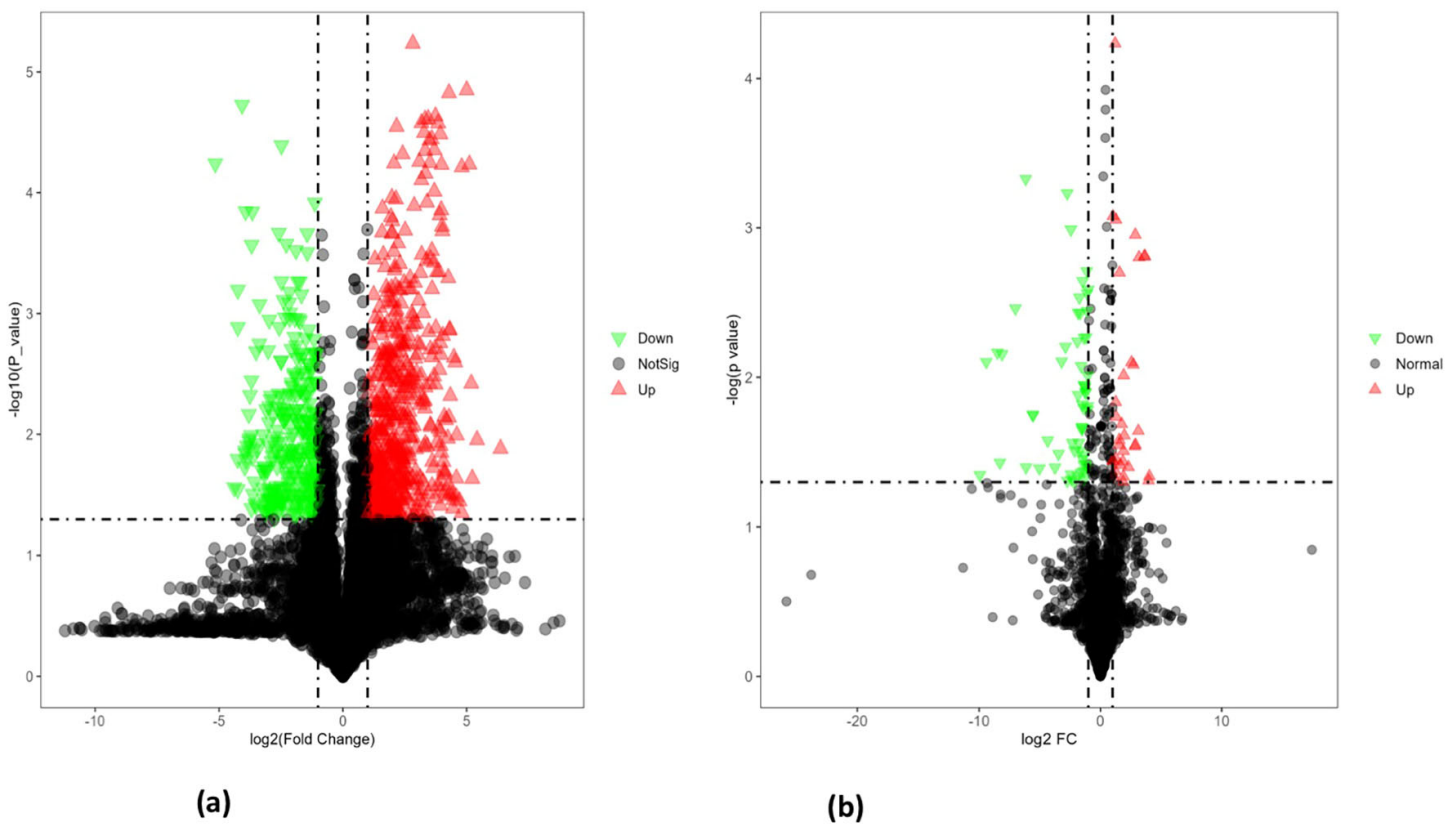
**(A)** The volcano plot for differentially expressed metabolites in PI518255 compared to PI598080 under non-stress condition. Red represents the up-regulated metabolites in PI518255 compared with PI598080, green represents the downregulated metabolites in PI518255 compared with PI598080, and gray represents the metabolites with no difference between PI518255 and PI598080, **(B)** volcano plot for differentially expressed lipids in PI518255 and PI598080. Red represents the up-regulated lipids in PI518255 compared with PI598080, green represents the downregulated lipids in PI518255 compared with PI598080, and gray represents the lipids with no difference between PI518255 and PI598080.

Volcano plot analysis (**Figures 8A, B**) of seeds from heat-tolerant and heat-sensitive genotypes under non-stress conditions revealed significant differences in metabolite and lipid levels. Specifically, the heat-tolerant genotypes had 28 upregulated and 58 downregulated metabolites, as well as 77 upregulated and 93 downregulated lipids (all at p ≤ 0.05) compared to the heat-sensitive genotype.

**Figure 9:**
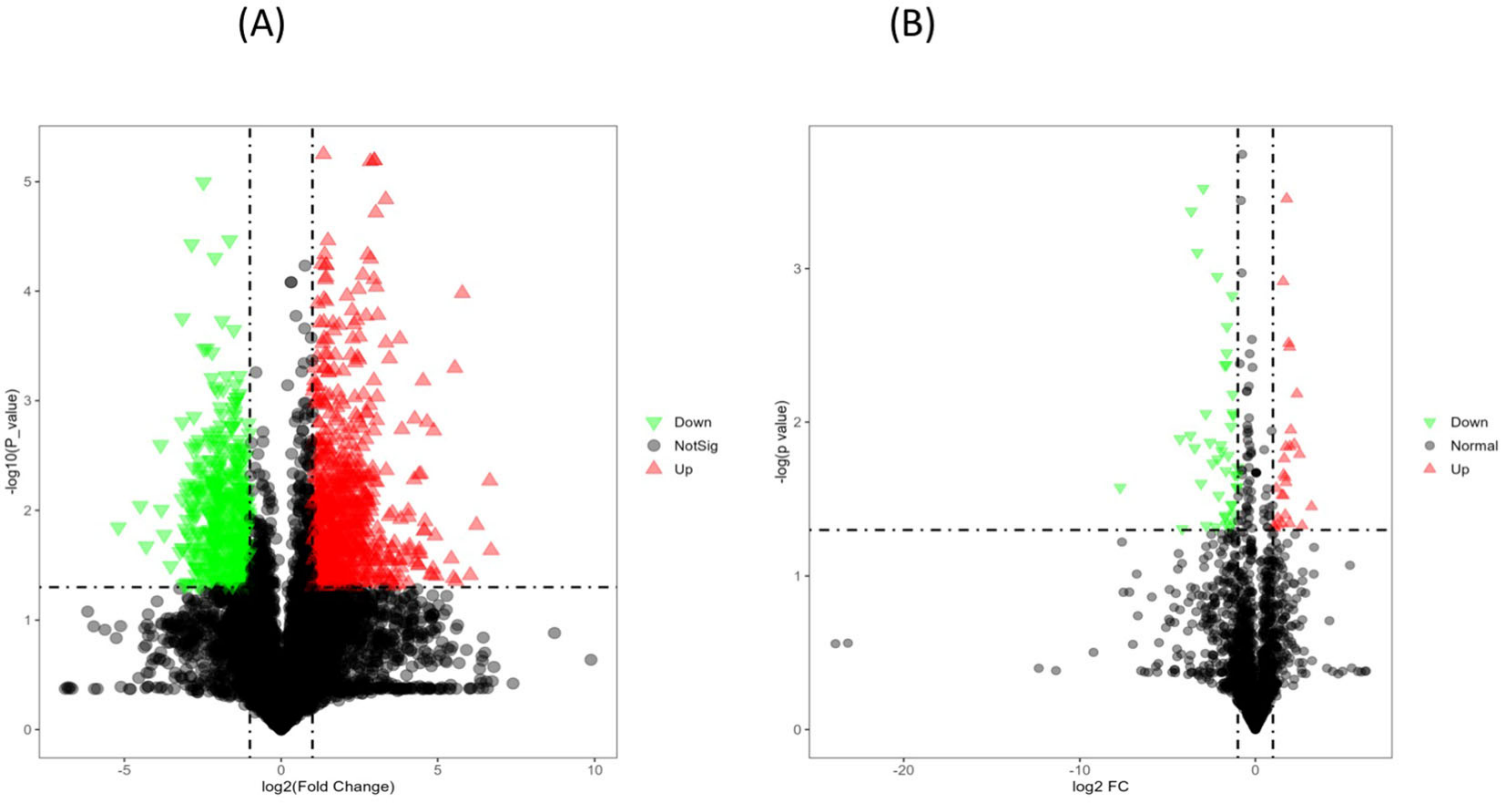
**(A)** The volcano plot for differentially expressed metabolites in PI518255 compared to PI598080 under heat-stress condition. Red represents the up-regulated metabolites in PI518255 compared with PI598080, green represents the downregulated metabolites in PI518255 compared with PI598080, and gray represents the metabolites with no difference between PI518255 and PI598080, **(B)** Volcano plot for differentially expressed lipids in PI518255 and PI598080. Red represents the up-regulated lipids in PI518255 compared with PI598080, green represents the downregulated lipids in PI518255 compared with PI598080, and gray represents the lipids with no difference between PI518255 and PI598080.

Similarly, under heat stress conditions (**Figures 9A, B**), the heat-tolerant genotypes showed 65 upregulated and 17 downregulated metabolites, along with 131 upregulated and 195 downregulated lipids compared to the heat-sensitive genotype.

### Metabolome profiling

Under non stress condition a total of 1,671 metabolites were detected in PI518255 and PI598080 seeds. Among them 28 were upregulated and 58 were downregulated in PI518255. Notable significantly upregulated metabolites included Moracetin, γ-Glutamylcysteinylγ-glutamylcysteinylglycine, N-(N-Acetylmethionyl) dopamine, Tricin 7-neohesperidoside and Pentacarboxyl porphyrinogen III [(VIP > 1.5, fold-change (|log_2_ FC| > 1), Student’s t-test (p < 0.05)]. The significantly down regulated metabolites included were Luteolin, Quercitrin, 5-Hydroxyferulic acid, Kaempferol and Diosmetin (**Table 1**; **Supplementary Table S2**).

**Table 1:** List of selected differentially expressed metabolite in the chickpea seeds of PI518255 compared to PI598080 under non stress condition.

SL. NO.	RT [min]	Molecular weight	m/z	HMDB_ID	Compound name	Chemical formula	Log2(FC)	T-Test	VIP	Regulation
1	5.681	286.04854	285.04127	HMDB0005801	Kaempferol	C15H10O6	-2.593	0.044	1.61	DR
2	6.006	286.0487	285.04142	HMDB0005800	Luteolin	C15H10O6	-2.783	0.039	1.59	DR
3	6.006	/	429.08409	HMDB0033751	Quercitrin	C21H20O11	-2.978	0.030	1.60	DR
4	4.684	788.20683	787.19955	HMDB0040487	Moracetin	C33H40O22	5.112	0.000	1.70	UR
5	4.9	/	419.09924	HMDB0035484	5-Hydroxyferulic acid	C10H10O5	-2.670	0.011	1.66	DR
6	6.661	/	371.1263	HMDB0244495	N-(N-Acetylmethionyl)dopamine	C15H22N2O4S	3.438	0.031	1.67	UR
7	1.617	539.13702	538.12975	CSID28184670	γ-Glutamylcysteinylγ-glutamylcysteinylglycine	C18H29N5O10S2	3.815	0.027	1.64	UR
8	5.442	638.18815	637.18087	HMDB0037462	Tricin 7-neohesperidoside	C29H34O16	3.718	0.034	1.60	UR
9	6.392	700.2767	699.26943	HMDB0001957	Pentacarboxyl porphyrinogen III	C37H40N4O10	3.654	0.019	1.64	UR
10	7.603	300.06291	301.07018	HMDB0029676	Diosmetin	C16H12O6	-4.97	0.035	1.66	DR

DR, downregulated; UR, upregulated.

A total of 1,671 metabolites were detected in PI518255 and PI598080 seeds under heat stress. Among these, 82 showed significant differential expression: 65 upregulated and 17 downregulated in PI518255. Notably upregulated metabolites included cinnamyl alcohol, leucyl-threonine, hesperetin 7-glucoside, 1-O-cinnamoyl-(6-arabinosylglucose), 3-feruloylquinic acid, moracetin, luteolin [(VIP > 1.5, fold-change (|log2 FC| >1), Student’s t-test (p < 0.05)]. In contrast, significantly downregulated metabolites included N-acetylphenylalanine, Frangulanine, Cortolone-3-glucuronide, Apigenin, and Olmesartan medoxomil (**Table 2**; **Supplementary Table S3**).

**Table 2:** List of selected differentially expressed metabolite in the chickpea seeds of PI518255 compared to PI598080 under heat tress condition.

SL. NO.	RT [min]	Molecular weight	m/z	HMDB_ID	Compound name	Chemical formula	Log2(FC)	T-Test	VIP	Regulation
1	4.764	368.11543	367.11	HMDB0030669	3-Feruloylquinic acid	C17H20O9	2.99	0.0212	1.54	UR
2	5.016	/	431.1	HMDB0033850	Astilbin	C21H22O11	3.04	0.0061	1.51	UR
3	4.755	442.15229	441.15	HMDB0030294	1-O-Cinnamoyl-(6-arabinosylglucose)	C20H26O11	4.87	0.0019	1.6	UR
4	4.747	/	509.13	HMDB0030747	Hesperetin 7-glucoside	C22H24O11	4.42	0.0227	1.53	UR
5	7.945	/	525.27	HMDB0255516	Neoandrographolide	C26H40O8	3.15	0.0016	1.51	UR
6	4.724	696.1584	695.15	HMDB0303154	Luteolin 7-O-(6''-O-malonyl)-diglucoside	C30H32O19	3	0.0457	1.57	UR
7	4.684	788.20683	787.2	HMDB0040487	Moracetin	C33H40O22	2.46	0.0112	1.56	UR
8	6.005	270.05249	271.06	HMDB0002124	Apigenin	C15H10O5	-2	2.82E-02	1.61	DR
9	4.115	558.21803	557.21	HMDB0255956	Olmesartan medoxomil	C29H30N6O6	-3.56	0.0105	1.56	DR
10	6.844	/	541.27	HMDB0010320	Cortolone-3-glucuronide	C27H42O11	-2.7	0.0327	1.51	DR
11	5.109	207.09016	206.08	HMDB0000512	N-Acetylphenylalanine	C11H13NO3	-1.65	0.001	1.61	DR
12	8.251	/	545.33	HMDB0030199	Frangulanine	C28H44N4O4	-1.52	0.0088	1.53	DR

DR, downregulated; UR, upregulated.

Under non stress condition a total of 10,846 lipids were detected, with 170 showing significant differential expression. Of these lipids, 77 were found to be upregulated in PI518255 and 93 were down regulated in PI518255. Notably upregulated lipids were DG (36:3e)+NH4, PE (36:2)+H, MG(16:0)+H, and PE(18:0_20:1)+Na. The notable down regulated lipids included MGDG (27:5e)-H, PA (38:1_11:4)-H, PEt(18:3_14:2)-H (See **Table 3**; **Supplementary Table S4**).

**Table 3:** List of selected differentially expressed lipids in seeds of PI518255 compared to PI598080 under non stress condition.

SL.NO	LipidIon	Lipid group	Class	FattyAcid	FA1	FA2	FA3	CalcMz	ObsMz	Rt	IonFormula	Log2(FC)	T-Test	VIP	Regulation
1	MGDG(27:5e)-H	MGDG(27:5e)-H	MGDG	(27:5e)	(27:5e)			635.416459	635.41646	3.3195	C36 H59 O9	-3.20	0.008	1.67	DR
2	PA(38:1_11:4)-H	PA(49:5)-H	PA	(38:1_11:4)	(38:1)	(11:4)		875.653532	875.65353	11.8795	C52 H92 O8 N0 P1	-2.43	0.001	1.72	DR
3	PEt(18:3_14:2)-H	PEt(32:5)-H	PEt	(18:3_14:2)	(18:3)	(14:2)		665.418782	665.41878	7.178784956	C37 H62 O8 N0 P1	-8.49	0.007	1.91	DR
4	PS(38:6+3O)-H	PS(38:6+3O)-H	PS	(38:6+3O)	(38:6+3O)			854.482506	854.48251	7.5255	C44 H73 O13 N1 P1	-1.10	0.003	1.90	DR
5	DG(36:3e)+NH4	DG(36:3e)+NH4	DG	(36:3e)	(36:3e)			622.576885	622.576374	11.7945	C39 H76 O4 N1	4.92	0.00	1.99	UR
6	PE(36:2)+H	PE(36:2)+H	PE	(36:2)	(36:2)			744.553783	744.55378	7.986	C41 H79 O8 N1 P1	2.80	0.02	1.88	UR
7	PS(36:1)+H	PS(36:1)+H	PS	(36:1)	(36:1)			790.559263	790.55926	8.074	C42 H81 O10 N1 P1	-2.17	0.01	1.89	DR
8	TG(16:0_16:1_20:1)+NH4	TG(52:2)+NH4	TG	(16:0_16:1_20:1)	(16:0)	(16:1)		876.801465	876.79872	7.941	C55 H106 O6 N1	3.36	0.00	1.94	UR
9	MG(16:0)+H	MG(16:0)+H	MG	(16:0)	(16:0)			331.284286	331.28429	5.8685	C19 H39 O4	2.19	0.01	1.51	UR
10	PE(18:0_20:1)+Na	PE(38:1)+Na	PE	(18:0_20:1)	(18:0)	(20:1)		796.582678	796.58268	9.016	C43 H84 O8 N1 P1 Na1	3.24	0.00	2.08	UR

MGDG, Monogalactosyldiacylglycerol; PA, phosphatidic acid; PE, phosphatidylethanolamine; Pet, Phosphatidylethanol; PS, phosphatidylserine; DG, Diacylglycerol; PE, phosphatidylethanolamine; TG, triglyceride; MG, monoacylglycerol; DR, downregulated; UP, upregulated.

Under heat stress condition of the significantly detected lipids, 326 showed significant differential expression under heat stress. Of these, 131 were upregulated in PI518255, and while 195 were downregulated in PI518255. Notable upregulated lipids included MGDG (34:5), PA(38:1), PC(20:0), PE (20:0), PI(18:1), PS, and dimethylphosphatidylethanolamine (dMePE), whereas notable downregulated lipids included BiotinylPE, MGDG(45:14e),OAHFA (12:0), PA(49:5), PC (29:2CHO), PG(29:1), PIP (18:2), PMe (48:5), and PS (18:1) (**Table 4**; **Supplementary Table S5**).

**Table 4:** List of selected differentially expressed lipids in seeds of PI518255 compared to PI598080 under heat tress condition.

Sl.no.	LipidIon	Lipidgroup	Class	FattyAcid	FA1	FA2	FA3	CalcMz	ObsMz	Rt	IonFormula	Log2(FC)	T-Test	VIP	Regulation
1	MGDG(45:14e)-H	MGDG(45:14e)-H	MGDG	(45:14e)	(45:14e)			869.557	869.56	11.076	C54 H77 O9	-4.18	0.05	1.81	DR
2	BiotinylPE(14:2_16:0)-H	BiotinylPE(30:2)-H	BiotinylPE	(14:2_16:0)	(14:2)	(16:0)		884.523	884.52	8.481	C45 H79 O10 N3 S1 P1	-2.09	0.03	1.83	DR
3	PMe(48:5)-H	PMe(48:5)-H	PMe	(48:5)	(48:5)			875.654	875.65	11.902	C52 H92 O8 N0 P1	-2.97	0	2.06	DR
4	PIP(18:2_11:4)-H	PIP(29:6)-H	PIP	(18:2_11:4)	(18:2)	(11:4)		835.344	835.34	0.7015	C38 H61 O16 N0 P2	-2.44	0.02	1.88	DR
5	PC(29:2CHO)+HCOO	PC(29:2CHO)+HCOO	PC	(29:2CHO)	(29:2CHO)			746.461	746.46	5.1525	C38 H69 O11 N1 P1	-3.7	0.01	1.92	DR
6	MGDG(45:6e)-H	MGDG(45:6e)-H	MGDG	(45:6e)	(45:6e)			885.683	885.68	11.865	C54 H93 O9	3.19	0.04	2.19	UR
7	PI(18:1e)-H	PI(18:1e)-H	PI	(18:1e)	(18:1e)			597.305	597.3	2.177	C27 H50 O12 N0 P1	2.51	0.02	2.04	UR
8	LPG(18:1)-H	LPG(18:1)-H	LPG	(18:1)	(18:1)			509.288	509.29	2.4095	C24 H46 O9 N0 P1	2.02	0.01	2.09	UR
9	DG(32:2e)+H	DG(32:2e)+H	DG	(32:2e)	(32:2e)			551.5	551.5	10.82	C35 H67 O4	4.12	0	2.15	UR
10	PS(36:4+4O)-H	PS(36:4+4O)-H	PS	(36:4+4O)	(36:4+4O)			846.477	846.48	9.864	C42 H73 O14 N1 P1	2.36	0.01	1.93	UR

MGDG, Monogalactosyldiacylglycerol; BiotinylPE, Biotinyl Phosphatidylethanolamine; PC, phosphatidylcholine; PMe, phosphatidylmonomethylethanolamine; PIP phosphatidylinositol phosphates; PI, phosphatidylinositol PI; LPG, lysophosphatidylglycerol; DG, Diacylglycerol; PS, phosphatidylserine; DR, downregulated; UP, upregulated.

**Figure 10:**
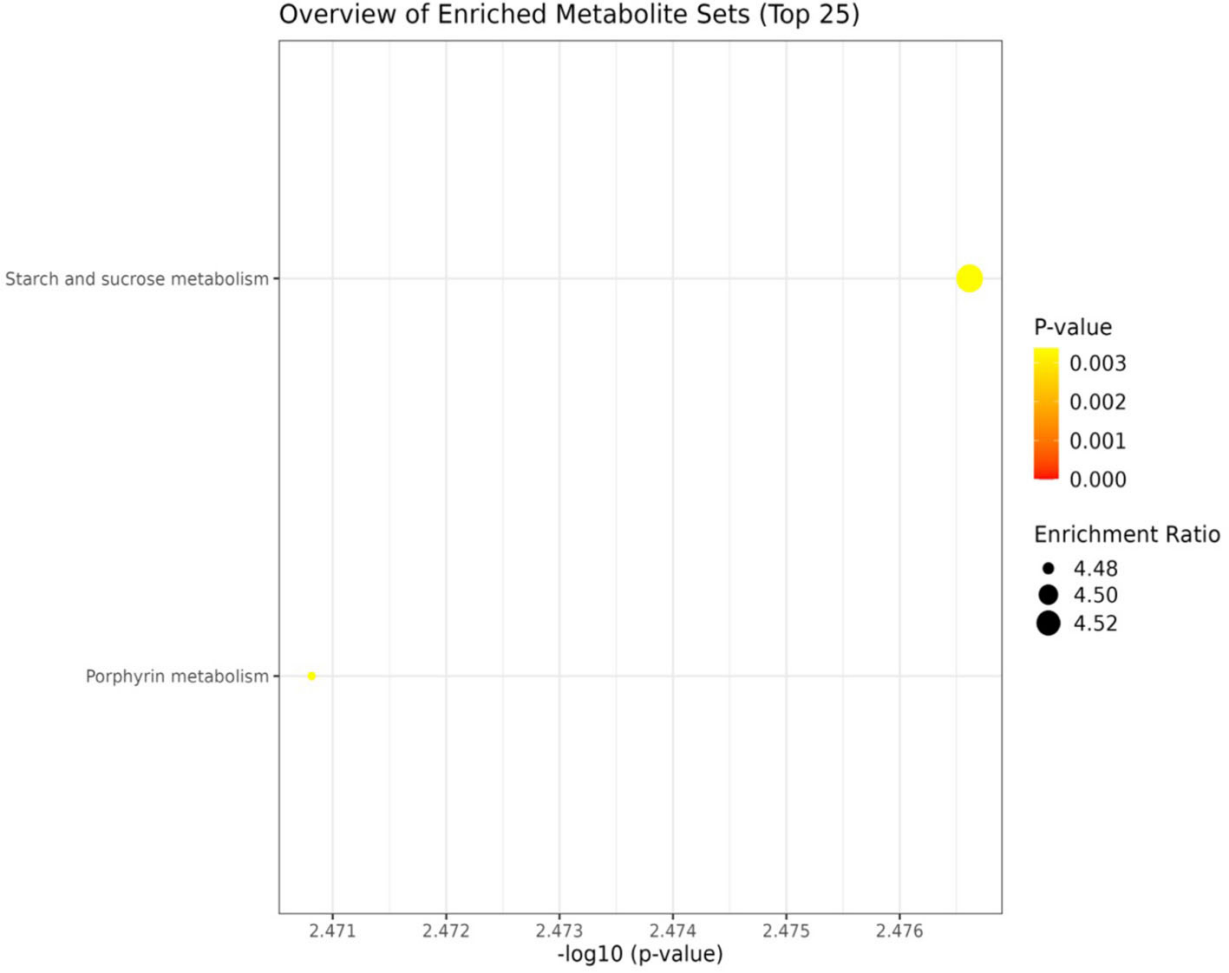
Network analysis plots of perturbed metabolites showed distinct patterns under non-stress condition. Metabolite pathway enrichment analysis further indicated enrichment in key metabolites in the starch and sucrose metabolism.

**Figure 11:**
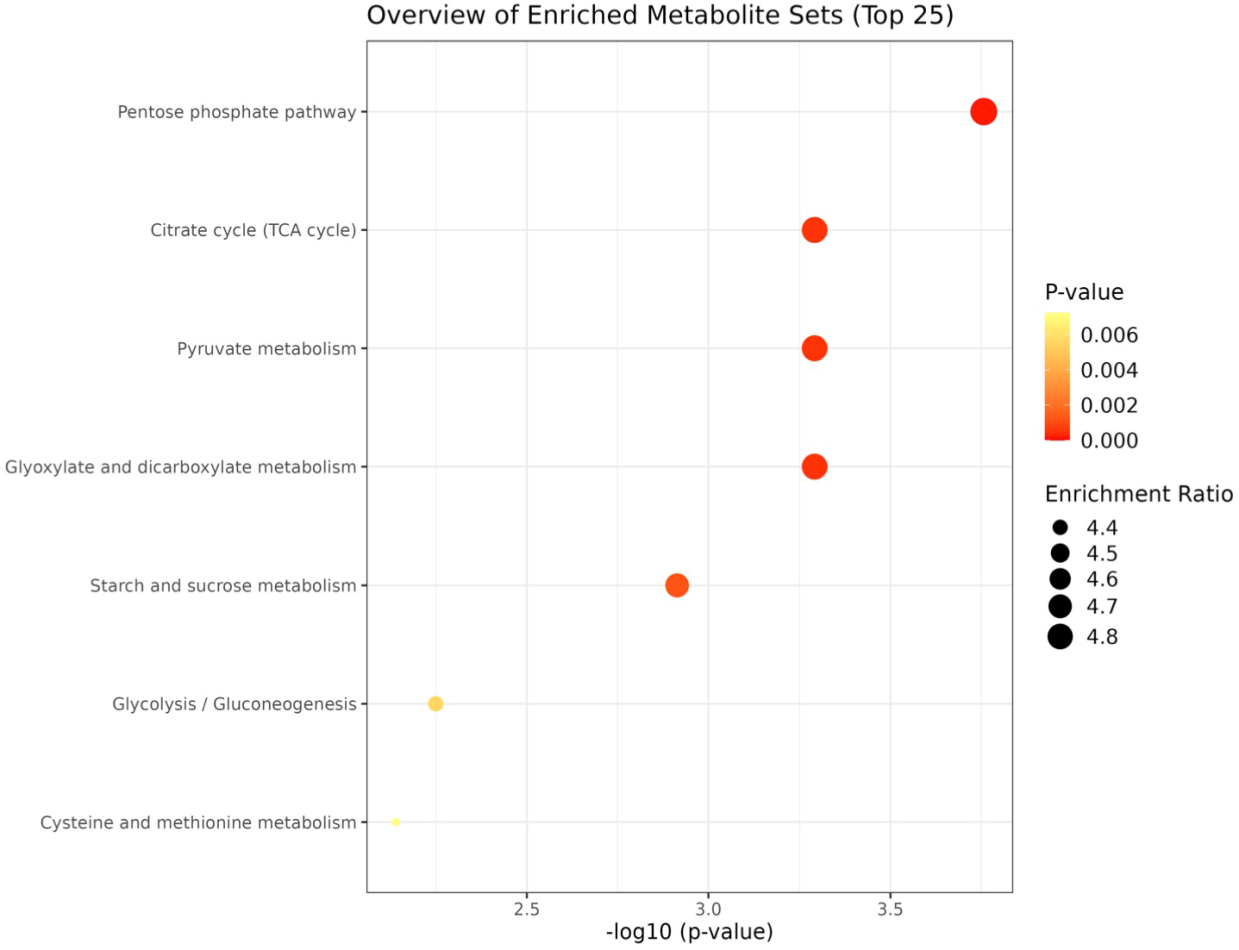
Network analysis plots of perturbed metabolites showed distinct patterns under heat stress condition. Metabolite pathway enrichment analysis further indicated enrichment in key metabolites in the TCA cycle, glycolysis/ gluconeogenesis, pentose phosphate pathway, glyoxylate, pyruvate and starch and sucrose metabolism.

KEGG based pathway enrichment analysis of significantly altered metabolites revealed major heat stress-responsive pathways in the heat-tolerant genotype. These included the pentose phosphate pathway, TCA cycle, pyruvate metabolism, starch and sucrose metabolism, glyoxylate and dicarboxylate metabolism, glycolysis and gluconeogenesis, as well as cysteine and methionine metabolism (**Figures 10**, **11**).

**Figure 12:**
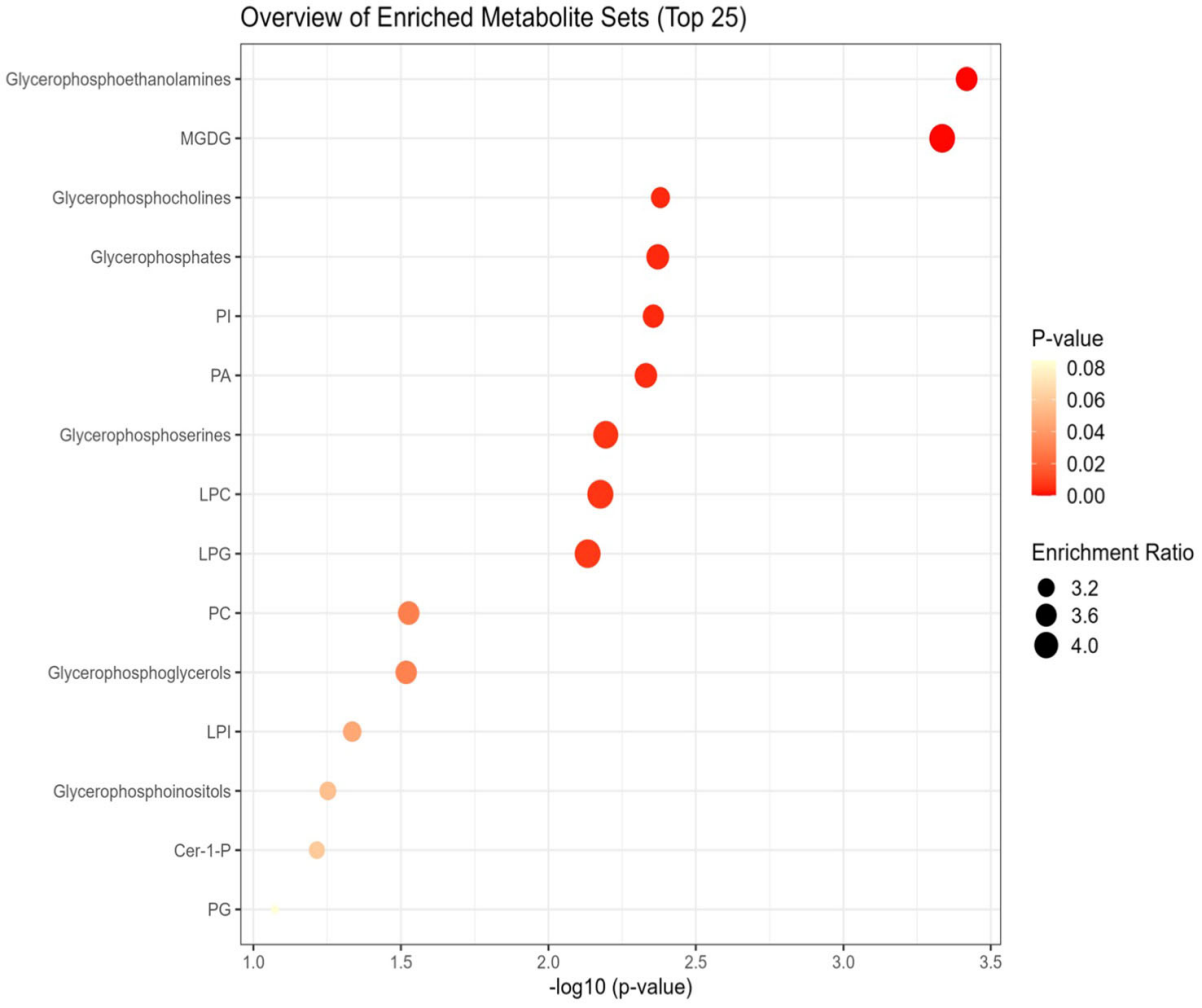
Network analysis plots of perturbed lipids revealed distinct patterns under non-stress. Lipid pathway analysis further indicated enrichment in key lipid classes, including Monogalactosyldiacylglycerol (MGDG), glycerophosphoethanolamine, glycerophosphocholine, glycerophosphoserine, glycerophosphates, phosphatidylinositol (PI), phosphatidic acid (PA), phosphatidylcholines (PC), lysophosphatidylinositol (LPI), lysophosphatidylcholine (LPC), lysophosphatidylglycerol (LPG), glycerophosphoinositol, and phosphoglyceric acid.

**Figure 13:**
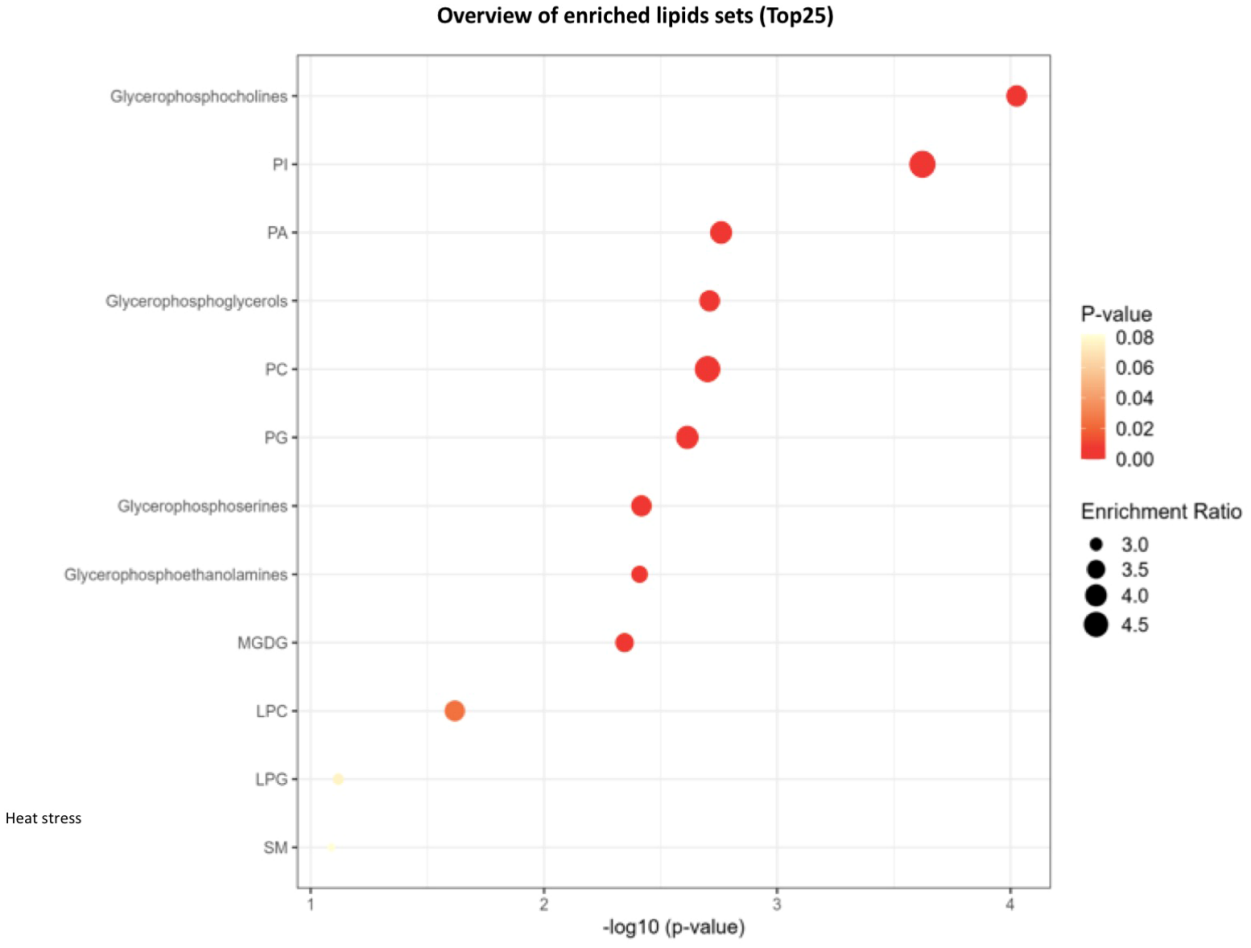
Network analysis plots of perturbed lipids revealed distinct patterns under heat stress conditions. Lipid pathway analysis further indicated enrichment in key lipid classes, including glycerophosphocholine, phosphatidylinositol (PI), phosphatidic acid (PA), phosphatidylcholines (PC), lysophosphatidylinositol (LPI), lysophosphatidylcholine (LPC), lysophosphatidylglycerol (LPG), Monogalactosyldiacylglycerol (MGDG), glycerophosphoserine, glycerophosphoethanolamine.

Lipid pathway analysis indicated enrichment in several key lipid classes, including MGDG, glycerophosphocholine, PI, PA, PC, LPI, LPC, LPG, glycerophosphoinositol, and phosphoglyceric acid under non stress and heat stress (**Figures 12**, **13**).

### Clustering analysis

**Figure 14:**
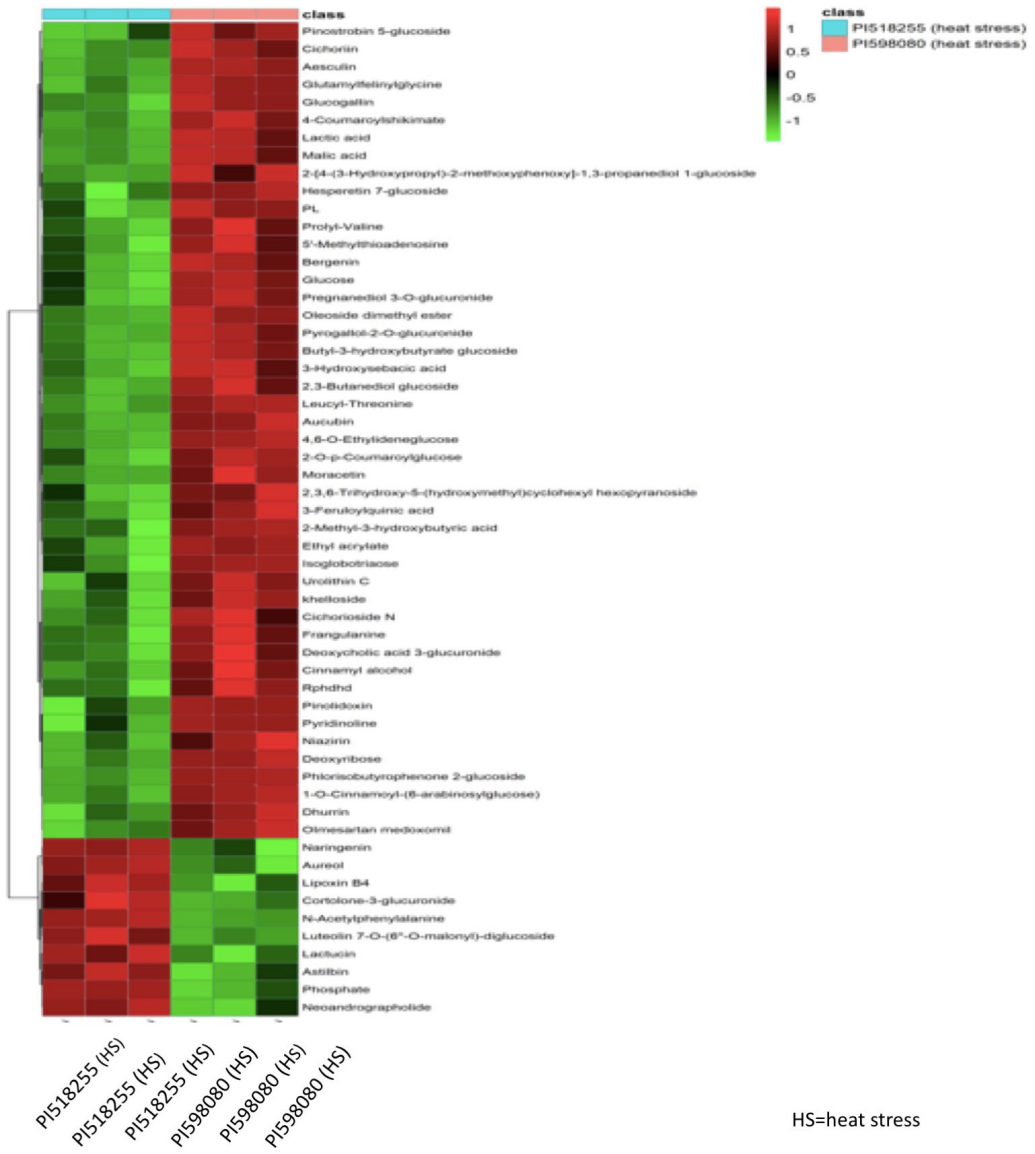
A hierarchical cluster analysis of metabolome data revealed that metabolites from two genotypes under heat stress were distinctly separated. The analysis showed that the PI518255 genotype had a higher accumulation of potential biomarker metabolites, including 3-Feruloylquinic acid, Hesperetin 7-glucoside, Leucyl-Threonine, Moracetin, Luteolin, Astilbin, 1-O-Cinnamoyl-(6-arabinosylglucose), and neoandrographolide.

**Figure 15:**
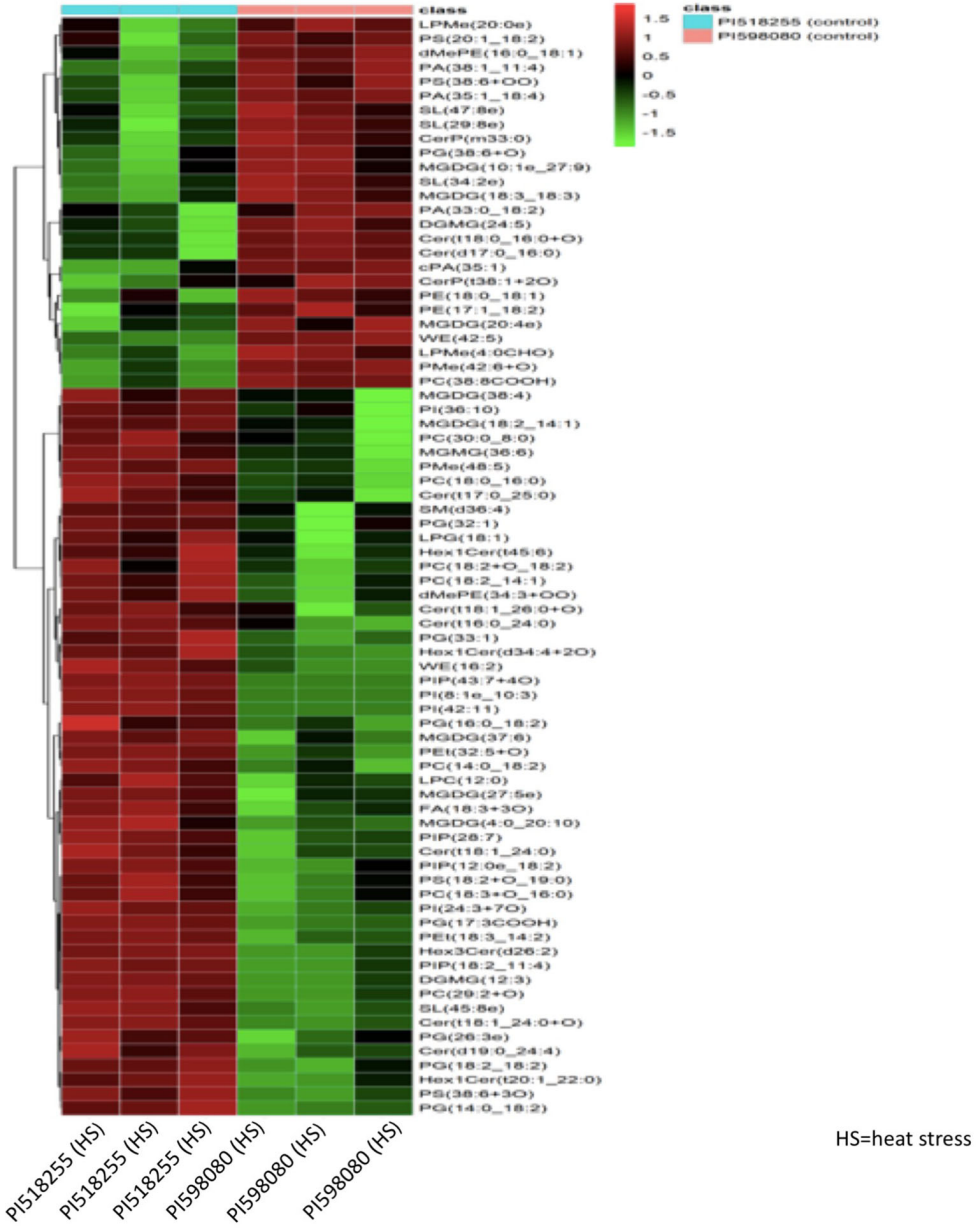
A hierarchical cluster analysis of lipidome data revealed a distinct separation of genotypes in response to heat stress. The analysis demonstrated that the PI518255 genotype accumulated elevated levels of several key lipids, specifically dimethylphosphatidylethanolamine (dMePE), phosphatidylinositol phosphates (PIP), phosphatidylethanolamine (PE), phosphatidylcholines (PC), phosphatidylglycerol (PG), phosphatidylinositol (PI), diacylglycerol monogalactoside (DGMG) (33:4), monogalactosyldiacylglycerol (MGDG) (45:6e), PC (49:5), phosphatidylserine (PS) (36:4), and PI (18:1e).

Hierarchical clustering analysis of differential metabolites (**Figure 14**) distinctly separated the two genotypes under heat stress, with PI518255 showing higher accumulation of potential biomarker metabolites such as 3-Feruloylquinic acid, Hesperetin 7-glucoside, Leucyl-Threonine, Moracetin, Luteolin, Astilbin1-O-Cinnamoyl-(6-arabinosylglucose), and neoandrographolide. Similarly, hierarchical clustering analysis of differential lipids (**Figure 15**) also separated the genotypes, with PI518255 displaying elevated levels of key lipids, including dMePE, PIP, PE, PC, PG, PI, DGMG (33:4), MGDG (45:6e), PC (49:5), PS (36:4), and PI (18:1e).

## Discussion

The increasing frequency and intensity of heat stress events threaten global food security, particularly for temperature-sensitive crops like chickpea. In addition to reducing overall yield, heat stress can severely impair seed biochemical composition and quality (Kumar et al., 2023a; Jha et al., 2024b). In this context, metabolomic and lipidomic profiling offers a valuable tool for identifying biochemical markers and mechanisms associated with heat stress tolerance. In the present study, the heat-tolerant genotype PI518255 exhibited higher effective pods per plant, seed number per plant, and seed yield per plant than heat-sensitive PI598080. These findings are consistent with previous reports of significant yield losses and compromised reproductive traits in chickpea exposed to heat stress (Devi et al., 2022; Jha et al., 2024c).

Among the various heat stress-responsive metabolites, flavonoids were key compounds associated with tolerance. These phenolic compounds play diverse roles in plant development and abiotic stress responses (Agati et al., 2012; Ma et al., 2014; Cui et al., 2023). In particular, naringenin is widely recognized for enhancing heat tolerance by increasing antioxidant activity, as demonstrated in tomato (Šamec et al., 2021). Likewise, naringenin ameliorated salinity and osmotic stress in *Phaseolus vulgaris* by scavenging reactive oxygen species (ROS), increasing glycine betaine, proline, and choline accumulation, and regulating nitrogen metabolism (Ozfidan-Konakci et al., 2020). Similarly, Guo et al. (2023) identified increased naringenin and its derivative chalcone in *P. ternate* under heat stress, further supporting its protective role.

Luteolin, another flavonoid elevated in PI518255 under heat stress and downregulated under non-stress, has been associated with enhanced antioxidant responses in pigeonpea exposed to heat, drought, and salinity (Song et al., 2022). Although astilbin is less studied in the context of heat stress, it has been implicated in aluminum stress tolerance in *Camellia drupifera* (Wang et al., 2023) and light-induced responses in grape cell cultures (Ayenew et al., 2015), suggesting a broader role in stress adaptation.

1-O-Cinnamoyl-(6-arabinosylglucose), an o-cinnamoyl glycoside and cinnamic acid derivative, belongs to the extensive class of phenolic compounds (Araniti et al., 2018; Hu et al., 2022). These compounds are synthesized through the phenylpropanoid pathway, a primary metabolic route for various secondary metabolites in plants (Hu et al., 2022). It is hypothesized to improve plant heat tolerance by enhancing antioxidant defenses, protecting cellular structures via reduced oxidative damage, and contributing to overall cellular homeostasis during high temperatures (Dai et al., 2012; Xue et al., 2025). For instance, cinnamic acid pretreatment has been demonstrated to alleviate heat stress in cucumber leaves by modulating antioxidant enzyme activity and decreasing lipid peroxidation (Dai et al., 2012). Likewise, hydroxycinnamic acid amides (HCAAs), also derived from the phenylpropanoid pathway, are crucial in abiotic stress responses due to their significant antioxidant properties (Xue et al., 2025). Consequently, the elevated levels of phenolic acid derivatives in the heat-tolerant PI518255 suggest their potential role in mitigating reactive oxygen species (ROS) damage under heat stress.

3-Feruloylquinic acid (3-FQA) is a phenolic acid that acts as a powerful antioxidant in a plant’s defense against abiotic stress, such as high light intensity, ultraviolet (UV) radiation, and drought (Šamec et al., 2021; Khan et al., 2024). Its accumulation is a key part of the plant’s metabolic response to these challenging environmental conditions, as 3-FQA and other phenolic compounds are synthesized through the phenylpropanoid pathway in response to the stress (Šamec et al., 2021).

The chemical structure of 3-FQA allows it to effectively scavenge free radicals, neutralizing their harmful effects and helping to restore cellular homeostasis. Research on *Rhododendron chrysanthum* has demonstrated that the application of abscisic acid (ABA), a key stress hormone, leads to an increased accumulation of 3-O-feruloylquinic acid derivatives, highlighting its integral role within a coordinated defense system (Guo et al., 2024). Ultimately, by neutralizing harmful ROS and modulating metabolic pathways, 3-FQA helps plants tolerate environmental stressors, allowing them to maintain growth and productivity.

The low accumulation of 5-hydroxyferulic acid under non-stress conditions, coupled with the enhanced accumulation of 3-FQA and related phenolic compounds in PI518255 under heat stress, suggests a promising strategy to mitigate the adverse effects of heat stress and enhance heat tolerance in chickpea.

Astilbin, a dihydroflavonol glycoside type of flavonoid present in plants such as *Smilax glabra Hypericum perforatum* (Xin et al., 2020; Jan et al., 2021) and reported in chickpea seed (Xiao et al., 2023), has garnered attention for its biological activities, particularly its antioxidant properties. Although specific research on astilbin’s role in plant heat tolerance is still developing, its chemical structure and established properties indicate a substantial contribution to plant resilience under high-temperature stress. This contribution likely stems from its proven ability to act as an antioxidant, thereby mitigating oxidative damage and supporting cellular stability. The increased abundance of astilbin in heat-tolerant PI518255 suggests they may help mitigate ROS damage under heat stress.

Moracetin, a flavonoid glycoside (quercetin-3-O-gentiotrioside), plays a key role in a plant’s defense against abiotic stress by acting as a powerful antioxidant (National Center for Biotechnology Information, 2025). Identified in plants like mulberry and *Tribulus terrestris*, moracetin’s protective capabilities are attributed to its quercetin core, which effectively scavenges harmful free radicals (Aslam et al., 2022). Its multiple hydroxyl groups allow it to neutralize reactive oxygen species (ROS), thereby protecting cellular components from damage (Akram et al., 2024). While specific research on moracetin is limited, its function as a quercetin derivative indicates that its primary role is to protect the plant from oxidative damage (Akram et al., 2024). The increased synthesis of such compounds in PI518255 genotype under non stress and in response to heat stress is a vital survival strategy for plants in harsh conditions.

Andrographolide, a diterpene lactone, is another compound of interest. Although its function in heat stress is not well documented, studies in *Andrographis paniculata* have linked it to drought and flooding stress responses (Chen et al., 2020; Singh et al., 2025). Its accumulation in the heat-tolerant chickpea genotype suggests a potential regulatory role in stress adaptation, meriting further research.

Lipids, essential structural and signaling molecules in plants (Narayanan et al., 2016, 2018), are particularly vulnerable to stress-induced peroxidation of their unsaturated fatty acids and other compositional changes, leading to membrane damage and potentially cell death (Liu and Huang, 2000). Profiling lipidomic changes under stress can thus offer valuable insights into the molecular basis of heat tolerance. Notably, elevated levels of saturated fatty acids may contribute to heat tolerance by reducing membrane fluidity (Escandón et al., 2018). However, crucial chloroplast galactolipids—such as digalactosyl diacylglycerol (DGDG) and monogalactosyl diacylglycerol (MGDG) are prone to ROS-induced peroxidation under heat stress (Farmer and Mueller, 2013). Under high temperatures, a significant decline in MGDG, phosphatidylglycerol (PG), phosphatidylcholine (PC), phosphatidic acid (PA), and lysophospholipids (LPG, LPC, and LPE) has been reported (Djanaguiraman et al., 2018), accompanied by a shift from highly unsaturated lipid species to less unsaturated ones. In wheat, the heat-tolerant cultivar showed an increased proportion of DGDG and phosphatidylinositol (PI), with the opposite trend in the heat-sensitive line (Hu et al., 2023). Additionally, the tolerant cultivar exhibited a smaller increase in the unsaturation levels of MGDG and phosphatidylethanolamine (PE) under heat stress than the sensitive cultivar (Hu et al., 2023). In line with these findings, our current study revealed enhanced expression of BiotinylPE, MGDG (45:6e), PC (49:5), PS (36:4), and PI (18:1e) in the heat-tolerant genotype PI518255, suggesting their possible contribution to overall heat tolerance.

Heat stress reprograms plant metabolic networks to mitigate damage by enhancing antioxidant defense systems and promoting the synthesis of protective metabolites (Janni et al., 2020; Xu et al., 2024). This reprogramming involves the activation of several key metabolic pathways associated with stress-responsive metabolite production (Janni et al., 2020). Among these, the TCA cycle, central to carbohydrate and lipid metabolism is particularly susceptible to heat stress (Xu et al., 2024). Heat-induced ROS accumulation inhibits the TCA cycle, reducing NADPH production, a crucial cofactor in redox homeostasis (Dumont and Rivoal, 2019 see **Supplementary Figure S2**). Additionally, heat stress has been shown to suppress glycolysis and gluconeogenesis, further disrupting central metabolism. However, it simultaneously enhances the synthesis of stress-associated amino acids such as proline and arginine, as observed in celery (*Apium graveolens* L.) (Li et al., 2022).

Heat stress can significantly alter lipid metabolism pathways, including MGDG, PI, PA, PC, glycerophosphocholines, and glycerophosphoserines. MGDG lipids primarily enrich the thylakoid membrane remodeling pathway in response to heat stress. By altering the saturation of fatty acids, MGDG makes the membrane more rigid, which protects and stabilizes the photosynthetic machinery (photosystems I and II) (Narayanan et al., 2016, 2018; Hölzl and Dörmann, 2007). This ensures the continued function of the photosynthetic electron transport chain, which is critical for producing energy and antioxidants.

Under heat stress, glycerophosphates are central to glycerophospholipid metabolism and membrane remodeling, which helps maintain membrane fluidity. They are actively synthesized, degraded, and modified to adjust the ratio of saturated to unsaturated fatty acids, thereby stabilizing membranes and protecting cellular components (Higashi and Saito, 2019).

Phosphatidylinositol lipids are not just structural components but are at the very beginning of a complex signaling cascade (Mishkind et al., 2009; Laxalt et al., 2025). Their metabolism is a primary event that allows the plant to perceive heat stress and quickly activate a coordinated defense response, from calcium signaling to the expression of protective heat shock proteins (Mishkind et al., 2009; Laxalt et al., 2025). These findings underscore the importance of lipid composition in maintaining membrane stability under heat stress. Consequently, future targeted lipidome investigations in chickpea could offer valuable insights into the lipid-mediated mechanisms underlying heat tolerance.

## Conclusions

The increasing frequency and intensity of heat stress events and the growing demand for nutritious food threaten global food security. In chickpea, heat stress significantly reduces grain yield and impairs seed quality. However, the biochemical basis of these effects, particularly at the metabolomic and lipidomic levels, remains insufficiently explored. Our results indicate that the plant’s strategy involves not only the production of protective metabolites like phenolic acids, flavonoids and terpenoids but also the dynamic remodeling of cellular membranes via lipids like MGDG, glycerophosphates, glycerophosphoserines, phosphatidylinositol and lysophosphatidylcholine. Upregulation of some unsaturated fatty acids help in maintaining membrane stability. This coordinated metabolic and lipid-signaling response allows the plant to perceive the stress signal, protect its cellular components, and survive under heat stress. Targeted metabolomics and lipidomics approaches have the potential to uncover novel heat-responsive biomarkers, which could be instrumental in distinguishing heat-tolerant from heat-sensitive chickpea genotypes and accelerating the development of climate-resilient cultivars through marker-assisted breeding and trait selection.

## Data Availability

The original contributions presented in the study are publicly available. This data can be found here: https://doi.org/10.6084/m9.figshare.30231736.
