# Supplementary material for: Metabolomic and lipidomic changes in heat-stressed chickpea seeds
Source: Front Plant Sci. 2025 Oct 13;16:1668751. doi: 10.3389/fpls.2025.1668751 (PMC12554600; doi:10.3389/fpls.2025.1668751)
Supplement: Supplementary file 1 [file DataSheet1.pdf]

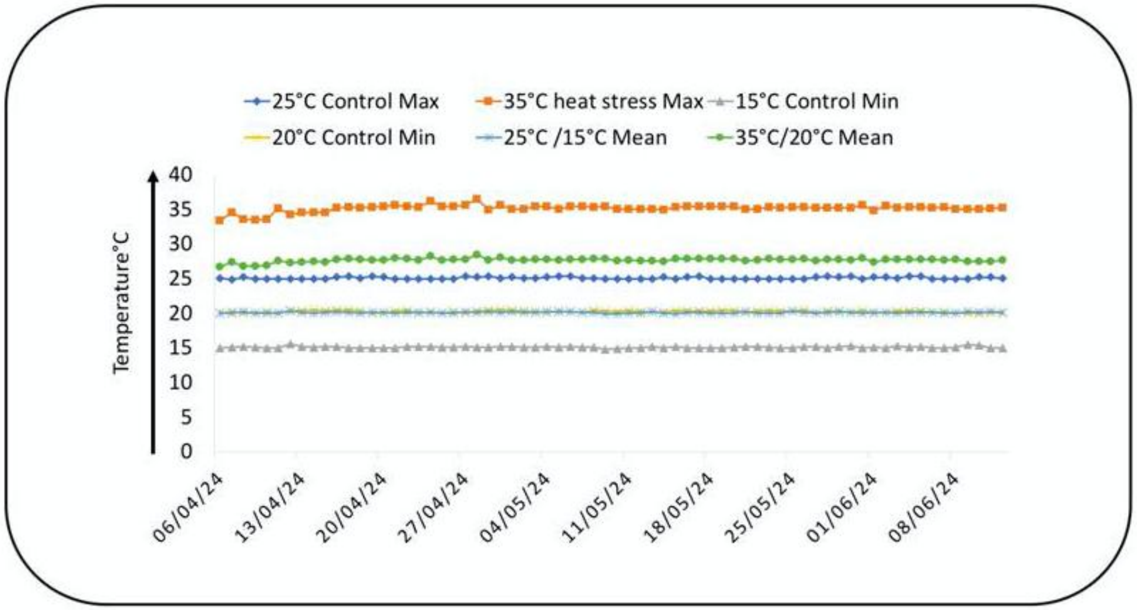

FigS1. Mean weekly temperature recorded during experiment in growth chamber

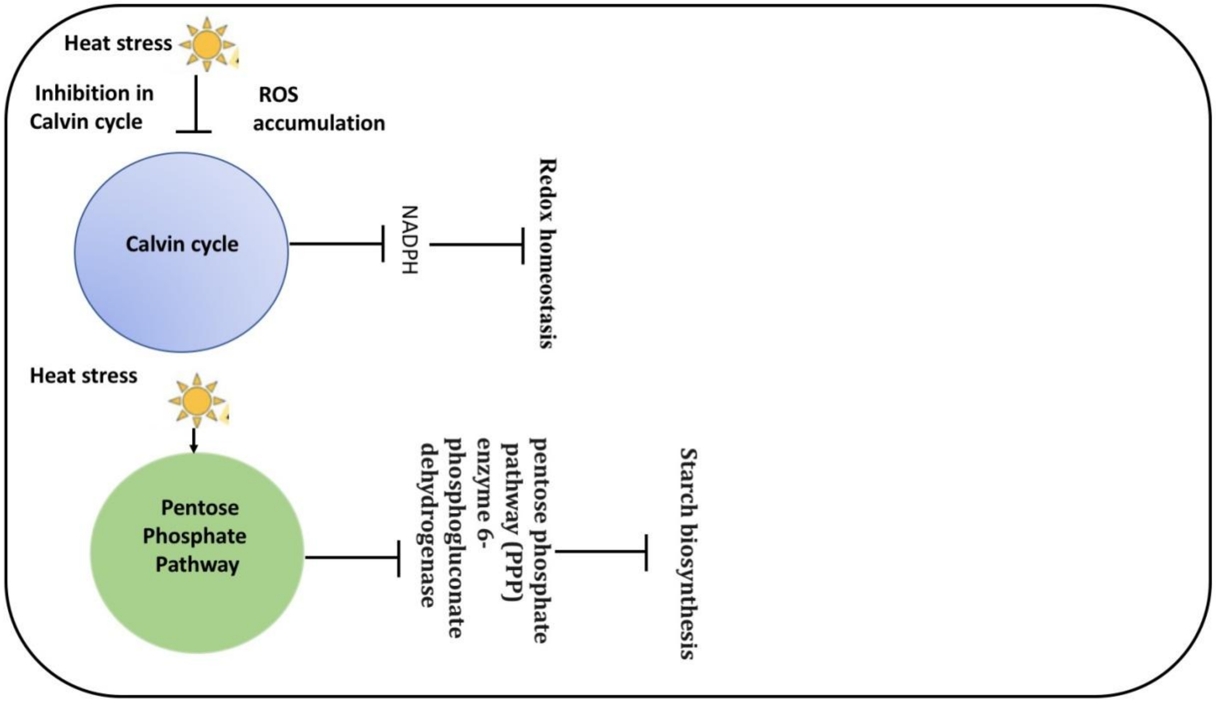

FigS2. Heat-induced reactive oxygen species accumulation inhibits the TCA cycle, reducing NADPH production, a crucial cofactor in redox homeostasis (Dumont and Rivoal, 2019). Likewise, pentose phosphate pathway (PPP) enzyme 6-phosphogluconate dehydrogenase (6PGDH) in plastid, is critical for endosperm starch accumulation (Ribeiro et al., 2020). Under heat stress, this enzyme is negatively affected and seed development is challenged.

Dumont, S., and Rivoal, J. (2019). Consequences of oxidative stress on plant glycolytic and 589 respiratory metabolism. Front. Plant Sci . 10, 166. doi: 10.3389/fpls.2019.00166.

Ribeiro, C., Hennen - Bierwagen, T.A., Myers, A.M., Cline, K., and Settles, A.M. (2020) 844 Engineering 6 - phosphogluconate dehydrogenase improves grain yield in heat - stressed 845 maize. Proc. Natl. Acad. Sci. U S A. 117(52), 33177 - 33185. doi: 846 10.1073/pnas.2010179117

Table S1. Mean value of morpho-physiological traits of contrasting genotypes under non stress and heat stress condition

Genotype	Days to first flowering	Days to first podding	Plant height(cm)	Days to maturity	Total effective pods/plant	Total seeds/plant	Seed yield/plant(g)
**Morph physiological traits of contrasting genotypes under non stress condition**							
PI518255	45	68	55	116	57	60	11.6
PI598080	48	72	51	119	50	52	9.6
**Morph physiological traits of contrasting genotypes under heat stress condition**							
Genotype	**Days to first flowering**	**Days to first podding**	**Plant height(cm)**	**Days to maturity**	**Total effective pods/plant**	**Total seeds/plant**	**Seed yield/plant(g)**
PI518255	40	58	49	98	42	49	5.2
PI598080	46	54	47	95	31	33	3.5

Table S2. Differentially expressed metabolites in seeds of PI518255 compared to PI598080 chickpea genotypes under non-stress

SL. NO.	RT [min]	Molecular Weight	m/z	HMDB_ID	Compound name	Chemical Formula	Log2(FC)	T-Test	VIP	Regulation
1	12.875	226.23025	271.22845	HMDB0031078	Pentadecanal	C15H30O	-2.078	0.011	1.58	DR
2	7.118	274.13257	273.12529	HMDB0247904	2-(p-Acetamidophenyl)-2-ethylglutarimide	C15H18N2O3	3.937	0.039	1.65	UR
3	5.681	286.04854	285.04127	HMDB0005801	Kaempferol	C15H10O6	-2.593	0.044	1.61	DR
4	6.006	286.0487	285.04142	HMDB0005800	Luteolin	C15H10O6	-2.783	0.039	1.59	DR
5	4.987	/	285.13507	CSID28698149	Methyl 9,12-dioxo-10-dodecenoate	C13H20O4	-1.170	0.007	1.63	DR
6	4.846	288.06415	287.05687	HMDB0005810	Eriodictyol	C15H12O6	-3.027	0.036	1.60	DR
7	7.628	316.05936	315.05208	HMDB0030547	3,4',5,7-Tetrahydroxy-6-methoxyflavone	C16H12O7	-2.678	0.034	1.66	DR
8	10.126	/	325.2031	HMDB0012535	12-HHT	C17H28O3	2.638	0.004	1.68	UR
9	6.737	332.16365	331.15638	HMDB0303452	Gibberellin A40	C19H24O5	-1.357	0.036	1.51	DR
10	4.663	/	335.20874	HMDB0040168	Glycerol 1-(5-hydroxydodecanoate)	C15H30O5	-2.201	0.025	1.54	DR
11	6.661	/	371.1263	HMDB0244495	N-(N-Acetylmethionyl)dopamine	C15H22N2O4S	3.438	0.031	1.67	UR
12	4.806	388.17458	387.1673	HMDB0040706	7-Epi-12-hydroxyjasmonic acid glucoside	C18H28O9	-2.098	0.008	1.66	DR
13	6.208	/	391.27177	HMDB0242055	N-Myristoyl Lysine	C20H40N2O3	2.048	0.008	1.64	UR
14	6.076	/	403.11601	HMDB0255634	Nitrendipine M (dehydro)	C18H18N2O6	-1.726	0.001	1.64	DR
15	4.9	/	419.09924	HMDB0035484	5-Hydroxyferulic acid	C10H10O5	-2.670	0.011	1.66	DR
16	6.006	/	429.08409	HMDB0033751	Quercitrin	C21H20O11	-2.978	0.030	1.60	DR
17	6.007	470.08376	469.07657	HMDB0037073	Resveratrol 3-glucoside 4'-sulfate	C20H22O11S	-3.041	0.042	1.59	DR
18	6.647	474.11758	473.11028	HMDB0039747	3''-O-Acetylafzelin	C23H22O11	-2.909	0.005	1.55	DR
19	6.921	/	477.14178	HMDB0040503	Pinostrobin 5-glucoside	C22H24O9	-3.696	0.041	1.63	DR
20	5.758	506.10766	505.10038	HMDB0029271	Quercetin 3-O-(6''-acetyl-glucoside)	C23H22O13	-3.644	0.015	1.67	DR
21	6.142	522.34223	521.3346	HMDB0037182	Polysorbate 20	C26H50O10	2.981	0.003	1.70	UR
22	5.675	/	535.11131	HMDB0301688	Quercetin 3-O-acetyl-rhamnoside	C23H22O12	-1.893	0.002	1.59	DR
23	1.617	539.13702	538.12975	CSID28184670	γ-Glutamylcysteinylγ-glutamylcysteinylglycine	C18H29N5O10S2	3.815	0.027	1.64	UR
24	7.383	/	547.3504	HMDB0000394	3-Hydroxytetradecanedioic acid	C14H26O5	2.824	0.023	1.67	UR
25	6.056	578.16542	577.15814	HMDB0304455	pelargonidin-3-O-rutinoside	C27H30O14	-3.686	0.000	1.71	DR
26	7.782	584.26576	583.25848	HMDB0000054	Bilirubin	C33H36N4O6	3.377	0.000	1.57	UR
27	5.517	594.16055	593.15328	HMDB0303597	Multiflorin B	C27H30O15	-2.380	0.015	1.67	DR
28	4.497	596.1763	595.16902	HMDB0038478	Rubrofusarin 6-gentiobioside	C27H32O15	-2.136	0.017	1.62	DR
29	3.907	612.17288	611.1656	HMDB0040677	Hydroxysafflor yellow A	C27H32O16	-2.284	0.000	1.65	DR
30	5.035	612.17297	611.1657	HMDB0030558	Safflomin A	C27H32O16	-2.836	0.011	1.64	DR

31	7.783	/	627.24954	HMDB0001008	Biliverdin	C33H34N4O6	3.698	0.000	1.67	UR
32	6.861	/	629.31955	HMDB0002577	Cholic acid glucuronide	C30H48O11	3.988	0.000	1.64	UR
33	5.726	/	634.18112	HMDB0255554	NeuAc(>6)GalNAc(alpha1->O)Ser	C22H37N3O16	-2.502	0.016	1.69	DR
34	5.442	638.18815	637.18087	HMDB0037462	Tricin 7-neohesperidoside	C29H34O16	3.718	0.034	1.60	UR
35	7.39	/	641.26481	HMDB0059948	Chlorin E6	C34H36N4O6	3.020	0.002	1.64	UR
36	5.728	644.13861	643.13133	CSID26331658	coenzyme F420-1	C24H29N4O15P	-2.474	0.036	1.56	DR
37	7.727	/	643.28075	HMDB0000668	Hematoporphyrin IX	C34H38N4O6	3.964	0.029	1.67	UR
38	5.628	/	656.14986	HMDB0037996	Cyanidin 3-laminaribioside	C27H31O16+	-2.479	0.001	1.70	DR
39	5.316	/	672.14609	HMDB0038007	Delphinidin 3-sophoroside	C27H31O17+	-2.031	0.044	1.66	DR
40	6.556	/	675.27217	HMDB0040917	Brassica napus non-fluorescent chlorophyll catabolite 3	C34H38N4O8	2.172	0.001	1.68	UR
41	5.673	/	677.21053	HMDB0029299	Sesamolinol 4'-O-glucosyl (1->6)-O-glucoside	C32H40O17	3.471	0.001	1.56	UR
42	5.937	/	677.25137	HMDB0251334	Dihydrotestosterone diglucuronide	C31H46O14	2.745	0.001	1.67	UR
43	6.102	/	683.23799	HMDB0035305	Kanokoside C	C27H42O17	2.093	0.000	1.56	UR
44	8.297	/	683.27704	HMDB0250580	Cucurbitacin B 2-sulfate	C32H46O11S	3.677	0.000	1.61	UR
45	6.392	700.2767	699.26943	HMDB0001957	Pentacarboxyl porphyrinogen III	C37H40N4O10	3.654	0.019	1.64	UR
46	5.311	726.20436	725.19708	HMDB0038767	Camelliaside B	C32H38O19	-1.725	0.020	1.68	DR
47	5.066	742.199	741.19173	HMDB0041412	Quercetin 3-(2Gal-apiosylrobinobioside)	C32H38O20	-1.672	0.001	1.69	DR
48	6.643	/	741.27895	HMDB0010332	Bilirubin glucuronide	C39H44N4O12	3.315	0.006	1.67	UR
49	5.024	756.21663	755.20936	HMDB0302425	Kaempferol 3-O-rhamnodiglucoside	C33H40O20	-2.498	0.027	1.60	DR
50	4.945	/	785.22035	HMDB0040562	Mauritianin	C33H40O19	-3.484	0.012	1.70	DR
51	4.684	788.20683	787.19955	HMDB0040487	Moracetin	C33H40O22	5.112	0.000	1.70	UR
52	5.312	/	788.19415	HMDB0301881	Cyanidin 3-O-(2-xylosyl-6-glucosyl-galactoside)	C32H39O20+	-1.955	0.022	1.68	DR
53	5.067	/	804.18886	HMDB0037090	Delphinidin 3-lathyroside 5-glucoside	C32H39O21+	-2.170	0.001	1.69	DR
54	5.402	/	818.20459	HMDB0303637	Delphinidin 3-rutinoside 5-glucoside	C33H41O21+	-2.136	0.001	1.69	DR
55	5.025	/	818.2047	HMDB0302726	Cyanidin 3-triglucoside	C33H41O21+	-2.806	0.030	1.61	DR
56	3.999	/	897.21622	HMDB0030684	Cyanidin 3-glucoside	C21H21O11+	-2.324	0.007	1.65	DR
57	4.098	212.06578	213.07307	HMDB0034493	Valtrate	C10H12O5	-1.06	0.010	1.52	DR
58	4.876	226.12021	227.12749	HMDB0059729	3,4-Methylenesebacic acid	C12H18O4	-2.19	0.021	1.64	DR
59	5.378	260.08932	261.09659	HMDB0032924	3-Furanmethanol glucoside	C11H16O7	-2.32	0.027	1.75	DR
60	0.989	/	266.15942	HMDB0031876	Prenyl glucoside	C11H20O6	-1.17	0.018	1.64	DR
61	6.903	/	287.05459	HMDB0002708	Cyanidin	C15H11O6	-1.84	0.047	1.65	DR
62	7.603	300.06291	301.07018	HMDB0029676	Diosmetin	C16H12O6	-4.97	0.035	1.66	DR
63	5.131	302.04207	303.04935	HMDB0005794	Quercetin	C15H10O7	-1.69	0.001	1.72	DR
64	5.891	316.05787	317.06515	HMDB0002655	Isorhamnetin	C16H12O7	-3.72	0.017	1.71	DR
65	5.359	326.13369	327.14097	HMDB0038708	Citrusin C	C16H22O7	-3.69	0.000	1.73	DR

66	8.07	348.22953	349.2368	HMDB0248693	Ataprost	C21H32O4	-1.10	0.011	1.57	DR
67	5.935	/	393.18776	HMDB0259793	Vernolide A	C21H28O7	-1.24	0.014	1.60	DR
68	5.692	448.09998	449.10725	HMDB0030864	Trifolin	C21H20O11	-2.32	0.001	1.76	DR
69	5.466	462.11559	463.12287	HMDB0030569	Swertiajaponin	C22H22O11	-1.87	0.002	1.74	DR
70	4.449	/	463.14162	HMDB0039860	Propylene glycol alginate	C17H28O13	2.49	0.035	1.69	UR
71	5.132	464.09497	465.10225	HMDB0030775	Quercetin 3-galactoside	C21H20O12	-1.36	0.004	1.70	DR
72	7.298	502.11086	503.11815	HMDB0041263	6''-O-Malonyldaidzin	C24H22O12	-2.32	0.003	1.71	DR
73	5.511	514.20174	515.20899	HMDB0032761	Nb-Feruloylserotonin glucoside	C26H30N2O9	-1.03	0.005	1.62	DR
74	7.118	/	529.33434	HMDB0242372	Cholylcysteine	C27H45NO6S	2.70	0.004	1.74	UR
75	5.501	550.0954	551.10267	HMDB0037368	Quercetin 3-(6''-malonyl-glucoside)	C24H22O15	-2.89	0.021	1.67	DR
76	5.185	/	561.19397	HMDB0039230	Citrusin A	C26H34O12	3.21	0.025	1.69	UR
77	6.16	564.11096	565.11825	HMDB0038820	Isorhamnetin 3-(6''-malonylglucoside)	C25H24O15	-3.49	0.007	1.67	DR
78	5.583	594.15752	595.16511	HMDB0037574	Biorobin	C27H30O15	-2.47	0.024	1.74	DR
79	6.125	620.28383	621.29113	CSID390447	Scilliroside	C32H44O12	3.12	0.008	1.72	UR
80	5.758	624.16811	647.1573	HMDB0037746	Keioside	C28H32O16	-2.18	0.000	1.74	DR
81	6.922	/	629.31357	HMDB0266631	PA(8:0/5-iso PGF2VI)	C29H51O11P	3.66	0.003	1.77	UR
82	4.86	/	635.15736	HMDB0030558	Safflomin A	C27H32O16	-3.59	0.007	1.74	DR
83	6.62	658.26228	659.26956	HMDB0059795	Gluten exorphin B5	C30H38N6O11	2.37	0.001	1.76	UR
84	5.089	756.2098	779.19897	HMDB0301689	Kaempferol 3-O-glucosyl-rhamnosyl-glucoside	C33H40O20	-2.00	0.012	1.65	DR
85	5.183	786.22043	809.20955	HMDB0038254	Isorhamnetin 3-O-[glucopyranosyl-(1->2)-[arhamnopyranosyl-(1->6)]-glucopyranoside]	C34H42O21	-2.02	0.031	1.58	DR
86	4.743	/	811.18964	HMDB0040487	Moracetin	C33H40O22	4.19	0.020	1.75	UR

DR=down regulation, UR=upregulation, VIP=Variable importance projection

Table S3. **Differentially expressed metabolites in seeds of PI518255 compared to PI598080 under heat stress**

	RT [min]	Molecular weight	m/z	HMDB_ID	Compound name	Chemical Formula	Log2(FC)	T-Test	Log10(P_value)	VIP	Regulation
1	1.038	/	71.01411	HMDB0000190	Lactic acid	C3H6O3	1.88	0.0232	1.63	1.57	UR
2	0.939	97.97717	96.9699	HMDB0001429	Phosphate	H3O4P	1.51	0.0002	3.65	1.56	UR
3	1.522	100.05286	99.04557	HMDB0033978	Ethyl acrylate	C5H8O2	-1.50	0.0006	3.26	1.55	DR
4	3.747	/	99.04556	HMDB0000354	2-Methyl-3-hydroxybutyric acid	C5H10O3	2.44	0.0004	3.40	1.55	UR
5	1.036	/	115.00407	HMDB0000156	Malic acid	C4H6O5	2.20	0.0242	1.62	1.58	UR
6	1.514	/	161.0462	HMDB0000122	Glucose	C6H12O6	1.31	0.0026	2.59	1.52	UR
7	5.042	134.07371	133.06644	HMDB0029697	Cinnamyl alcohol	C9H10O	1.62	0.0437	1.36	1.54	UR
8	1.526	/	249.09878	HMDB0029030	Prolyl-Valine	C10H18N2O3	1.28	0.0056	2.25	1.54	UR
9	5.109	207.09016	206.08289	HMDB0000512	N-Acetylphenylalanine	C11H13NO3	-1.65	0.0000	4.47	1.61	DR
10	5.462	218.11615	217.10888	HMDB0000350	3-Hydroxysebacic acid	C10H18O5	1.36	0.0050	2.30	1.54	UR
11	7.216	/	271.06192	HMDB0002670	Naringenin	C15H12O5	1.41	0.0009	3.03	1.53	UR
12	4.149	/	233.10371	HMDB0040822	2,3-Butanediol glucoside	C10H20O7	-2.73	0.0114	1.94	1.56	DR
13	5.039	244.03544	243.02817	HMDB0029218	Urolithin C	C13H8O5	1.67	0.0048	2.32	1.53	UR
14	7.842	/	257.08275	HMDB0035814	Lactucin	C15H16O5	-1.26	0.0028	2.56	1.53	DR
15	1.048	/	267.10905	HMDB0003224	Deoxyribose	C5H10O4	3.45	0.0113	1.95	1.60	UR
16	1.369	/	267.1092	HMDB0028939	Leucyl-Threonine	C10H20N2O4	2.47	0.0041	2.38	1.59	UR
17	4.39	/	278.06782	HMDB0001173	5'-Methylthioadenosine	C11H15N5O3S	2.98	0.0263	1.58	1.50	UR
18	3.659	/	292.08354	HMDB0060471	Dhurrin	C14H17NO7	1.17	0.0023	2.63	1.54	UR
19	1.501	302.06241	301.05513	HMDB0060017	Pyrogallol-2-O-glucuronide	C12H14O9	2.93	0.0047	2.33	1.58	UR
20	4.008	/	303.14602	HMDB0031694	Butyl-3-hydroxybutyrate glucoside	C14H26O8	1.11	0.0010	3.00	1.57	UR
21	3.824	/	313.05762	HMDB0038728	Glucogallin	C13H16O10	1.33	0.0003	3.52	1.59	UR
22	4.276	326.10469	325.09742	HMDB0039167	2-O-p-Coumaroylglucose	C15H18O8	2.59	0.0026	2.59	1.56	UR
23	3.883	/	329.02913	HMDB0037921	Aureol	C15H8O6	-3.85	0.0025	2.60	1.57	DR
24	7.841	/	333.20832	HMDB0005082	Lipoxin B4	C20H32O5	1.48	0.0035	2.46	1.52	UR
25	6.901	340.08072	339.07344	HMDB0030821	Cichoriin	C15H16O9	-1.54	0.0018	2.74	1.57	DR

26	6.703	340.08075	339.07347	HMDB0030820	Aesculin	C15H16O9	1.39	0.0000	4.34	1.60	UR
27	4.751	/	339.11319	HMDB0029651	Phlorisobutyrophenone 2-glucoside	C16H22O9	5.55	0.0005	3.30	1.60	UR
28	1.602	340.13823	339.13095	CSID112595	2,3,6-Trihydroxy-5-(hydroxymethyl) cyclohexyl hexopyranoside	C13H24O10	2.34	0.0162	1.79	1.50	UR
29	2.089	346.12759	345.12031	HMDB0036562	Aucubin	C15H22O9	1.55	0.0013	2.88	1.59	UR
30	5.717	/	365.08918	HMDB0304174	4-Coumaroylshikimate	C16H16O7	1.05	0.0007	3.13	1.58	UR
31	4.764	368.11543	367.10815	HMDB0030669	3-Feruloylquinic acid	C17H20O9	2.99	0.0212	1.67	1.54	UR
32	3.833	/	373.07918	HMDB0249087	Bergenin	C14H16O9	1.68	0.0039	2.41	1.53	UR
33	5.654	/	373.14192	HMDB0301836	Pinolidoxin	C18H26O6	1.40	0.0005	3.29	1.54	UR
34	3.736	393.15843	392.15115	HMDB0252619	Glutamylfelinylglycine	C15H27N3O7S	1.68	0.0002	3.64	1.59	UR
35	6.202	408.10729	407.10002	CSID390535	khelloside	C19H20O10	1.51	0.0028	2.56	1.55	UR
36	4.738	/	409.17289	HMDB0000851	Pyridinoline	C18H28N4O8	1.98	0.0005	3.27	1.51	UR
37	2.111	/	411.15225	HMDB0246615	4,6-O-Ethylideneglucose	C8H14O6	3.04	0.0001	4.04	1.60	UR
38	5.62	418.18519	417.17791	HMDB0040353	2-[4-(3-Hydroxypropyl)-2-methoxyphenoxy]-1,3-propanediol 1-glucoside	C19H30O10	1.52	0.0398	1.40	1.55	UR
39	5.016	/	431.09985	HMDB0033850	Astilbin	C21H22O11	3.04	0.0061	2.21	1.51	UR
40	4.755	442.15229	441.14501	HMDB0030294	1-O-Cinnamoyl-(6-arabinosylglucose)	C20H26O11	4.87	0.0019	2.72	1.60	UR
41	4.152	/	453.11588	HMDB0031350	Oleoside dimethyl ester	C18H26O11	3.05	0.0015	2.82	1.59	UR
42	5.802	/	471.22498	HMDB0302058	Cichorioside N	C22H34O8	-1.22	0.0102	1.99	1.51	DR
43	6.921	/	477.14178	HMDB0040503	Pinostrobin 5-glucoside	C22H24O9	2.50	0.0057	2.24	1.51	UR
44	4.264	/	483.17332	HMDB0006598	Isoglobotriaose	C19H34O15	3.35	0.0003	3.53	1.52	UR
45	6.844	/	541.26699	HMDB0010320	Cortolone-3-glucuronide	C27H42O11	-2.70	0.0327	1.49	1.51	DR
46	8.05	504.33127	503.32399	HMDB0257327	Rphdhd	C26H48O9	1.16	0.0077	2.12	1.52	UR
47	4.747	/	509.13198	HMDB0030747	Hesperetin 7-glucoside	C22H24O11	4.42	0.0227	1.64	1.53	UR
48	7.945	/	525.27195	HMDB0255516	Neoandrographolide	C26H40O8	3.15	0.0016	2.80	1.51	UR
49	9.865	/	531.27406	HMDB0010318	Pregnanediol 3-O-glucuronide	C27H44O8	2.93	0.0041	2.39	1.52	UR
50	8.586	542.31012	541.30285	HMDB0302594	PL	C28H46O10	2.51	0.0016	2.80	1.54	UR
51	8.251	/	545.33419	HMDB0030199	Frangulanine	C28H44N4O4	-1.52	0.0088	2.06	1.53	DR
52	4.115	558.21803	557.21076	HMDB0255956	Olmesartan medoxomil	C29H30N6O6	-3.56	0.0105	1.98	1.56	DR
53	3.963	/	557.21122	HMDB0032807	Niazirin	C14H17NO5	3.11	0.0318	1.50	1.53	UR

8.253	568.32475	567.31746	HMDB0002596	Deoxycholic acid 3-glucuronide	C30H48O10	1.52	0.0103	1.99	1.53	UR
4.724	696.1584	695.15112	HMDB0303154	Luteolin 7-O-(6''-O-malonyl)-diglucoside	C30H32O19	3.00	0.0457	1.34	1.57	UR
4.684	788.20683	787.19955	HMDB0040487	Moracetin	C33H40O22	2.46	0.0112	1.95	1.56	UR
1.142	78.01385	79.02113	HMDB0002151	Dimethyl sulfoxide	C2H6OS	3.619563	1.000000E-03	4.906	1.63	UR
3.767	/	118.06493	HMDB0000738	Indole	C8H7N	-1.1477748	1.813914E-02	1.741	1.51	DR
1.051	/	104.10678	HMDB0000097	Choline	C5H14NO	1.0465464	1.964875E-05	4.707	1.64	DR
4.65	143.07346	144.08074	HMDB0243964	1-Naphthylamine	C10H9N	-1.4152292	7.760124E-03	2.110	1.56	DR
5.111	158.03667	159.04394	HMDB0244101	1,2-Naphthoquinone	C10H6O2	1.4546305	4.666224E-02	1.331	1.59	UR
1.483	197.06622	198.07349	HMDB0000181	Dopa	C9H11NO4	2.6584174	5.138255E-03	2.289	1.63	UR
4.361	198.08902	199.09629	HMDB0242172	Decadienedioic acid	C10H14O4	3.2238663	1.949283E-02	1.710	1.60	UR
6.005	270.05249	271.05976	HMDB0002124	Apigenin	C15H10O5	-2.0414075	2.817212E-02	1.550	1.61	DR
8.643	302.22409	303.23136	HMDB0001999	Eicosapentaenoic acid	C20H30O2	1.7725291	6.675389E-04	3.176	1.64	UR
1.817	343.08761	344.09489	HMDB0037261	DHBOA-Glc	C14H17NO9	1.4257889	2.129413E-03	2.672	1.58	UR
4.335	/	349.09231	HMDB0039167	2-O-p-Coumaroylglucose	C15H18O8	2.1929617	1.298253E-02	1.887	1.53	UR
4.363	/	361.14869	HMDB0028764	Aspartyl-Tryptophan	C15H17N3O5	4.1853149	1.266876E-02	1.897	1.58	UR
8.59	362.20861	363.21589	HMDB0000063	Cortisol	C21H30O5	-1.5627076	1.728679E-03	2.762	1.51	DR
4.88	402.11391	403.12119	HMDB0059971	4-Hydroxy-5-(3',4'-dihydroxyphenyl) valeric acid-O-glucuronide	C17H22O11	2.6069444	2.983310E-02	1.525	1.53	UR
5.496	/	407.09435	HMDB0033344	Mollicellin H	C21H20O6	2.9697775	5.165216E-03	2.287	1.55	UR
4.101	/	413.14106	HMDB0037260	Todatriol glucoside	C17H26O10	2.7588136	2.825450E-05	4.549	1.65	UR
5.064	/	415.12062	HMDB0034249	Caryoptosidic acid	C16H24O11	1.8300015	1.089496E-02	1.963	1.52	UR
4.295	432.12492	433.13164	HMDB0036523	Licoagroside B	C18H24O12	1.0633404	1.805325E-02	1.743	1.53	UR
4.803	432.16025	433.16752	HMDB0041515	Benzyl gentiobioside	C19H28O11	2.081033	1.925016E-03	2.716	1.59	UR
5.929	460.19182	461.1991	HMDB0255115	N-Desethyl Vardenafil	C21H28N6O4S	3.3659833	2.466690E-02	1.608	1.64	UR
6.362	488.16544	489.17271	HMDB0034280	Egonol glucoside	C25H28O10	2.4478484	7.577608E-04	3.120	1.61	UR
8.001	502.25371	503.26099	HMDB0247277	7-O-Succinyl macrolactin A	C28H38O8	-3.1055776	1.250292E-03	2.903	1.50	DR
4.231	/	665.28828	CSID10128138	3’-Deoxydihydrostreptomycin 3”-phosphate	C21H42N7O14P	2.7401898	1.930335E-02	1.714	1.64	UR
5.082	680.15765	681.16497	HMDB0301684	Chrysoeriol 7-O-(6''-malonyl-apiosyl-glucoside)	C30H32O18	-3.1126203	1.790069E-02	1.747	1.59	DR

Table S4. Differentially accumulated lipids in seeds of PI518255 compared to PI598080 under non-stress

SL.NO	LipidIon	LipidGroup	Class	FattyAcid	FA1	FA2	FA3	CalcMz	ObsMz	Rt	IonFormula	Log2(FC)	T-Test	VIP	Regulation
1	Cer(d17:0_16:0)+HCOO	Cer(d33:0)+HCOO	Cer	(d17:0_16:0)	(d17:0)	(16:0)		570.51	570.51	9.085	C34 H68 O5 N1	3.64	0.002	1.74	UR
2	Cer(t34:0+O)-H	Cer(t34:0+O)-H	Cer	(t18:0_16:0+O)	(t18:0)	(16:0+O)		570.51	570.51	9.084	C34 H68 O5 N1	3.64	0.002	1.74	UR
3	Cer(t16:0_24:0)+HCOO	Cer(t40:0)+HCOO	Cer	(t16:0_24:0)	(t16:0)	(24:0)		684.61	684.61	11.17	C41 H82 O6 N1	-2.88	0.006	1.76	DR
4	Cer(t18:1_24:0)-H	Cer(t42:1)-H	Cer	(t18:1_24:0)	(t18:1)	(24:0)		664.62	664.62	11.42	C42 H82 O4 N1	1.92	0.010	1.65	UR
5	DGMG(12:3)-H	DGMG(12:3)-H	DGMG	(12:3)	(12:3)			591.27	591.27	3.823	C27 H43 O14	-9.41	0.008	1.92	DR
6	Hex1Cer(d34:4+2O)+HCOO	Hex1Cer(d34:4+2O)+HCOO	Hex1Cer	(d34:4+2O)	(d34:4+2O)			770.51	770.51	7.524	C41 H72 O12 N1	-1.01	0.003	1.93	DR
7	Hex1Cer(t20:1_22:0)+HCOO	Hex1Cer(t42:1)+HCOO	Hex1Cer	(t20:1_22:0)	(t20:1)	(22:0)		872.68	872.68	10.72	C49 H94 O11 N1	-2.74	0.001	1.95	DR
8	Hex1Cer(t20:1_22:0)+HCOO	Hex1Cer(t42:1)+HCOO	Hex1Cer	(t20:1_22:0)	(t20:1)	(22:0)		872.68	872.68	10.99	C49 H94 O11 N1	-1.17	0.005	1.82	DR
9	Hex3Cer(d26:2)+HCOO	Hex3Cer(d26:2)+HCOO	Hex3Cer	(d26:2)	(d26:2)			954.53	954.53	10.35	C45 H80 O20 N1	-8.11	0.007	1.92	DR
10	MGDG(27:5e)-H	MGDG(27:5e)-H	MGDG	(27:5e)	(27:5e)			635.42	635.42	3.32	C36 H59 O9	-3.20	0.008	1.67	DR
11	MGDG(37:6)-H	MGDG(37:6)-H	MGDG	(37:6)	(37:6)			787.54	787.54	8.366	C46 H75 O10	-1.73	0.003	1.78	DR
12	PA(38:1_11:4)-H	PA(49:5)-H	PA	(38:1_11:4)	(38:1)	(11:4)		875.65	875.65	11.69	C52 H92 O8 N0 P1	1.57	0.002	1.92	UR
13	PA(38:1_11:4)-H	PA(49:5)-H	PA	(38:1_11:4)	(38:1)	(11:4)		875.65	875.65	11.88	C52 H92 O8 N0 P1	-2.43	0.001	1.72	DR
14	PA(35:1_18:4)-H	PA(53:5)-H	PA	(35:1_18:4)	(35:1)	(18:4)		931.72	931.72	11.85	C56 H100 O8 N0 P1	2.87	0.001	1.83	UR
15	PC(14:0_18:2)+HCOO	PC(32:2)+HCOO	PC	(14:0_18:2)	(14:0)	(18:2)		774.53	774.53	8.284	C41 H77 O10 N1 P1	-1.04	0.010	1.80	DR
17	PE(18:0_18:1)-H	PE(36:1)-H	PE	(18:0_18:1)	(18:0)	(18:1)		744.55	744.55	9.789	C41 H79 O8 N1 P1	2.72	0.008	1.64	UR
18	PEt(18:3_14:2)-H	PEt(32:5)-H	PEt	(18:3_14:2)	(18:3)	(14:2)		665.42	665.42	7.179	C37 H62 O8 N0 P1	-8.49	0.007	1.91	DR
19	PEt(32:5+O)-H	PEt(32:5+O)-H	PEt	(32:5+O)	(32:5+O)			681.41	681.41	6.928	C37 H62 O9 N0 P1	-7.01	0.003	1.91	DR
20	PG(14:0_18:2)-H	PG(32:2)-H	PG	(14:0_18:2)	(14:0)	(18:2)		717.47	717.47	7.274	C38 H70 O10 N0 P1	-1.13	0.002	1.93	DR
21	PG(33:1)-H	PG(33:1)-H	PG	(33:1)	(33:1)			733.5	733.5	8.153	C39 H74 O10 N0 P1	-1.36	0.005	1.90	DR
22	PG(18:2_18:2)-H	PG(36:4)-H	PG	(18:2_18:2)	(18:2)	(18:2)		769.5	769.5	7.524	C42 H74 O10 N0 P1	-1.67	0.004	1.81	DR
23	PIP(12:0e_18:2)-H	PIP(30:2e)-H	PIP	(12:0e_18:2)	(12:0e)	(18:2)		843.44	843.44	7.324	C39 H73 O15 N0 P2	-1.59	0.008	1.76	DR
24	PIP(43:7+4O)-H	PIP(43:7+4O)-H	PIP	(43:7+4O)	(43:7+4O)			1093.5	1093.5	1.167	C52 H87 O20 N0 P2	-6.16	0.000	1.99	DR
25	PMe(42:6+O)-H	PMe(42:6+O)-H	PMe	(42:6+O)	(42:6+O)			805.54	805.54	9.906	C46 H78 O9 N0 P1	1.03	0.001	1.91	UR
26	PMe(48:5)-H	PMe(48:5)-H	PMe	(48:5)	(48:5)			875.65	875.65	11.9	C52 H92 O8 N0 P1	-1.46	0.002	1.80	DR
27	PS(38:6+3O)-H	PS(38:6+3O)-H	PS	(38:6+3O)	(38:6+3O)			854.48	854.48	7.526	C44 H73 O13 N1 P1	-1.10	0.003	1.90	DR
28	SL(45:8e)-H	SL(45:8e)-H	SL	(45:8e)	(45:8e)			961.61	961.61	9.056	C54 H89 O12 S1	-1.84	0.004	1.92	DR

29	SM(d36:4)+HCOO	SM(d36:4)+HCOO	SM	(d36:4)	(d36:4)		769.55	769.55	9.198	C42 H78 O8 N2 P1	-1.90	0.006	1.51	DR
30	WE(16:2)-H	WE(16:2)-H	WE	(16:2)	(16:2)		251.2	251.2	3.432	H27 C16 O2	-1.11	0.004	1.93	DR
32	cPA(35:1)-H	cPA(35:1)-H	cPA	(35:1)	(35:1)		655.51	655.51	10.87	C38 H72 O6 N0 P1	3.18	0.002	1.81	UR
33	dMePE(16:0_18:1)-H	dMePE(34:1)-H	dMePE	(16:0_18:1)	(16:0)	(18:1)	744.55	744.55	9.754	C41 H79 O8 N1 P1	2.56	0.008	1.77	UR
34	AEA(20:0)+H	AEA(20:0)+H	AEA	(20:0)	(20:0)		356.35	356.35	1.272	C22 H46 O2 N1	-1.58	0.02	1.94	DR
35	AcHexCmE(21:1)+H	AcHexCmE(21:1)+H	AcHexCmE	(21:1)	(21:1)		869.72	869.72	11.57	C55 H97 O7	2.49	0.01	1.94	UR
36	BisMePA(14:0_21:1)+NH4	BisMePA(35:1)+NH4	BisMePA	(14:0_21:1)	(14:0)	(21:1)	734.57	734.57	9.605	C40 H81 O8 N1 P1	-3.09	0.00	2.03	DR
37	BisMePA(36:3)+NH4	BisMePA(36:3)+NH4	BisMePA	(36:3)	(36:3)		744.55	744.55	7.986	C41 H79 O8 N1 P1	2.80	0.02	1.88	UR
38	BisMePA(40:3)+Na	BisMePA(40:3)+Na	BisMePA	(40:3)	(40:3)		805.57	805.57	8.42	C45 H83 O8 N0 P1 Na1	-1.44	0.02	1.82	DR
39	Cer(d26:0+O)+H	Cer(d26:0+O)+H	Cer	(d26:0+O)	(d26:0+O)		444.4	444.4	11.17	C26 H54 O4 N1	3.95	0.00	1.89	UR
40	Cer(d13:0_15:0+O)+H	Cer(d28:0+O)+H	Cer	(d13:0_15:0+O)	(d13:0)	(15:0+O)	472.44	472.44	8.902	C28 H58 O4 N1	-2.66	0.02	1.90	DR
41	Cer(d13:0_17:0+O)+H	Cer(d30:0+O)+H	Cer	(d13:0_17:0+O)	(d13:0)	(17:0+O)	500.47	500.47	8.899	C30 H62 O4 N1	-1.54	0.03	1.62	DR
42	Cer(d32:0+O)+H	Cer(d32:0+O)+H	Cer	(d32:0+O)	(d32:0+O)		528.5	528.5	4.192	C32 H66 O4 N1	-1.11	0.05	1.67	DR
43	Cer(d23:2_16:0+O)+H	Cer(d39:2+O)+H	Cer	(d23:2_16:0+O)	(d23:2)	(16:0+O)	622.58	622.58	11.79	C39 H76 O4 N1	4.71	0.00	2.07	UR
44	Cer(d18:1_24:0+O)+H	Cer(d42:1+O)+H	Cer	(d18:1_24:0+O)	(d18:1)	(24:0+O)	666.64	666.64	11.24	C42 H84 O4 N1	-1.05	0.02	1.87	DR
45	Cer(m36:2)+NH4	Cer(m36:2)+NH4	Cer	(m36:2)	(m36:2)		565.57	565.57	10.76	C36 H73 O2 N2	-3.37	0.00	2.10	DR
46	Cer(m39:2+2O)+H	Cer(m39:2+2O)+H	Cer	(m39:2+2O)	(m39:2+2O)		622.58	622.58	11.79	C39 H76 O4 N1	4.11	0.00	2.10	UR
47	Cer(t18:1_21:1)+H	Cer(t39:2)+H	Cer	(t18:1_21:1)	(t18:1)	(21:1)	622.58	622.58	11.79	C39 H76 O4 N1	4.92	0.00	1.99	UR
48	Cer(t42:1)+H	Cer(t42:1)+H	Cer	(t42:1)	(t42:1)		666.64	666.64	11.24	C42 H84 O4 N1	-1.05	0.02	1.87	DR
49	DG(10:0)+Na	DG(10:0)+Na	DG	(10:0)	(10:0)		283.15	283.15	0.432	C13 H24 O5 Na1	-1.23	0.02	1.87	DR
50	DG(10:0)+Na	DG(10:0)+Na	DG	(10:0)	(10:0)		283.15	283.15	1.104	C13 H24 O5 Na1	2.29	0.00	2.06	UR
51	DG(10:0)+Na	DG(10:0)+Na	DG	(10:0)	(10:0)		283.15	283.15	10.17	C13 H24 O5 Na1	2.24	0.02	1.94	UR
52	DG(10:0)+Na	DG(10:0)+Na	DG	(10:0)	(10:0)		283.15	283.15	10.7	C13 H24 O5 Na1	1.59	0.02	1.87	UR
53	DG(15:1COOH)+H	DG(15:1COOH)+H	DG	(15:1COOH)	(15:1COOH)		359.21	359.21	6.641	C18 H31 O7	-1.68	0.04	1.69	DR
54	DG(18:0e)+NH4	DG(18:0e)+NH4	DG	(18:0e)	(18:0e)		376.34	376.34	6.182	C21 H46 O4 N1	-1.29	0.04	1.67	DR
55	DG(18:1e)+H	DG(18:1e)+H	DG	(18:1e)	(18:1e)		357.3	357.3	8.641	C21 H41 O4	2.41	0.03	1.85	UR
56	DG(18:5e)+H	DG(18:5e)+H	DG	(18:5e)	(18:5e)		349.24	349.24	2.981	C21 H33 O4	2.93	0.00	1.89	UR
57	DG(21:7CHO)+H	DG(21:7CHO)+H	DG	(21:7CHO)	(21:7CHO)		415.21	415.21	8.889	C24 H31 O6	2.27	0.00	1.99	UR
58	DG(22:1CHO)+NH4	DG(22:1CHO)+NH4	DG	(22:1CHO)	(22:1CHO)		458.35	458.35	12.31	C25 H48 O6 N1	-1.54	0.01	1.87	DR
59	DG(22:1e)+H	DG(22:1e)+H	DG	(22:1e)	(22:1e)		413.36	413.36	7.253	C25 H49 O4	2.22	0.00	2.05	UR
60	DG(23:0COOH)+NH4	DG(23:0COOH)+NH4	DG	(23:0COOH)	(23:0COOH)		490.37	490.37	10.77	C26 H52 O7 N1	1.58	0.03	1.88	UR
61	DG(11:0_12:1)+NH4	DG(23:1)+NH4	DG	(11:0_12:1)	(11:0)	(12:1)	458.38	458.38	8.424	C26 H52 O5 N1	2.07	0.01	1.80	UR
62	DG(23:1)+NH4	DG(23:1)+NH4	DG	(23:1)	(23:1)		458.38	458.38	9.413	C26 H52 O5 N1	-1.47	0.02	1.75	DR
63	DG(23:1COOCH3)+NH4	DG(23:1COOCH3)+NH4	DG	(23:1COOCH3)	(23:1COOCH3)		502.37	502.37	11.97	C27 H52 O7 N1	2.09	0.02	1.83	UR
64	DG(23:1e)+Na	DG(23:1e)+Na	DG	(12:1e_11:0)	(12:1e)	(11:0)	449.36	449.36	8.394	C26 H50 O4 Na1	-4.49	0.00	2.09	DR
65	DG(23:1e)+NH4	DG(23:1e)+NH4	DG	(23:1e)	(23:1e)		444.4	444.4	11.17	C26 H54 O4 N1	3.95	0.00	1.89	UR

66	DG(23:1e)+NH4	DG(23:1e)+NH4	DG	(23:1e)	(23:1e)		444.4	444.4	12.78	C26 H54 O4 N1	-2.79	0.01	1.79	DR
67	DG(24:2+OO)+NH4	DG(24:2+OO)+NH4	DG	(24:2+OO)	(24:2+OO)		502.37	502.37	12.9	C27 H52 O7 N1	4.11	0.01	1.98	UR
68	DG(25:1e)+H	DG(25:1e)+H	DG	(25:1e)	(25:1e)		455.41	455.41	9.388	C28 H55 O4	2.29	0.00	2.04	UR
69	DG(25:3COOH)+H	DG(25:3COOH)+H	DG	(25:3COOH)	(25:3COOH)		495.33	495.33	6.544	C28 H47 O7	-2.52	0.03	1.92	DR
70	DG(25:4e)+H	DG(25:4e)+H	DG	(25:4e)	(25:4e)		449.36	449.36	4.872	C28 H49 O4	2.24	0.03	1.75	UR
71	DG(26:2+3O)+NH4	DG(26:2+3O)+NH4	DG	(26:2+3O)	(26:2+3O)		546.4	546.4	3.439	C29 H56 O8 N1	1.69	0.04	1.79	UR
72	DG(28:1e)+NH4	DG(28:1e)+NH4	DG	(28:1e)	(28:1e)		514.48	514.48	11.82	C31 H64 O4 N1	-1.60	0.03	1.75	DR
73	DG(18:1e_10:0)+NH4	DG(28:1e)+NH4	DG	(18:1e_10:0)	(18:1e)	(10:0)	514.48	514.48	14.94	C31 H64 O4 N1	-1.84	0.03	1.88	DR
74	DG(28:2+4O)+NH4	DG(28:2+4O)+NH4	DG	(28:2+4O)	(28:2+4O)		590.43	590.43	1.949	C31 H60 O9 N1	-1.23	0.03	1.60	DR
75	DG(30:2+5O)+NH4	DG(30:2+5O)+NH4	DG	(30:2+5O)	(30:2+5O)		634.45	634.45	7.272	C33 H64 O10 N1	-2.39	0.05	1.83	DR
76	DG(30:2e)+NH4	DG(30:2e)+NH4	DG	(30:2e)	(30:2e)		540.5	540.5	8.388	C33 H66 O4 N1	-3.38	0.01	2.01	DR
77	DG(31:4e)+H	DG(31:4e)+H	DG	(31:4e)	(31:4e)		533.46	533.45	5.898	C34 H61 O4	4.95	0.00	1.90	UR
78	DG(32:1e)+Na	DG(32:1e)+Na	DG	(32:1e)	(32:1e)		575.5	575.5	8.402	C35 H68 O4 Na1	2.20	0.03	1.80	UR
79	DG(32:4CHO)+NH4	DG(32:4CHO)+NH4	DG	(32:4CHO)	(32:4CHO)		592.46	592.45	7.612	C35 H62 O6 N1	-1.82	0.04	1.55	DR
80	DG(34:2e)+Na	DG(34:2e)+Na	DG	(34:2e)	(34:2e)		601.52	601.52	10.5	C37 H70 O4 Na1	-3.81	0.02	2.04	DR
81	DG(12:1e_22:2)+Na	DG(34:3e)+Na	DG	(12:1e_22:2)	(12:1e)	(22:2)	599.5	599.5	11.31	C37 H68 O4 Na1	2.85	0.01	1.78	UR
82	DG(36:3e)+NH4	DG(36:3e)+NH4	DG	(36:3e)	(36:3e)		622.58	622.58	11.79	C39 H76 O4 N1	4.92	0.00	1.99	UR
83	DG(36:5e)+H	DG(36:5e)+H	DG	(36:5e)	(36:5e)		601.52	601.52	10.49	C39 H69 O4	-3.52	0.02	2.00	DR
84	DG(36:7e)+H	DG(36:7e)+H	DG	(36:7e)	(36:7e)		597.49	597.49	11.14	C39 H65 O4	1.40	0.01	1.84	UR
85	DG(39:2e)+NH4	DG(39:2e)+NH4	DG	(39:2e)	(39:2e)		666.64	666.64	11.24	C42 H84 O4 N1	-1.05	0.02	1.87	DR
86	DLCL(26:5)+Na	DLCL(26:5)+Na	DLCL	(26:5)	(26:5)		805.33	805.33	0.701	C35 H60 O15 P2 Na1	-3.52	0.04	2.01	DR
87	Hex1Cer(t42:1+O)+H	Hex1Cer(t42:1+O)+H	Hex1Cer	(t42:1+O)	(t42:1+O)		844.69	844.69	10.84	C48 H94 O10 N1	-3.89	0.00	1.94	DR
88	Hex1SPH(m17:1)+NH4	Hex1SPH(m17:1)+NH4	Hex1SPH	(m17:1)	(m17:1)		449.36	449.36	8.408	C23 H49 O6 N2	-2.86	0.00	2.00	DR
89	Hex1SPH(m17:1)+NH4	Hex1SPH(m17:1)+NH4	Hex1SPH	(m17:1)	(m17:1)		449.36	449.36	15.42	C23 H49 O6 N2	1.29	0.01	1.99	UR
90	Hex2Cer(d30:2)+H	Hex2Cer(d30:2)+H	Hex2Cer	(d30:2)	(d30:2)		804.55	804.55	8.798	C42 H78 O13 N1	-1.81	0.01	1.78	DR
91	LPC(8:2COOH)+Na	LPC(8:2COOH)+Na	LPC	(8:2COOH)	(8:2COOH)		432.14	432.14	0.816	C16 H28 O9 N1 P1 Na1	3.59	0.04	2.01	UR
92	LPMe(4:0COOH)+Na	LPMe(4:0COOH)+Na	LPMe	(4:0COOH)	(4:0COOH)		309.03	309.03	1.01	C8 H15 O9 N0 P1 Na1	2.29	0.03	1.97	UR
93	LPMe(8:1CHO)+H	LPMe(8:1CHO)+H	LPMe	(8:1CHO)	(8:1CHO)		325.1	325.1	1.13	C12 H22 O8 N0 P1	2.42	0.01	1.90	UR
94	LdMePE(9:2COOH)+Na	LdMePE(9:2COOH)+Na	LdMePE	(9:2COOH)	(9:2COOH)		432.14	432.14	0.816	C16 H28 O9 N1 P1 Na1	3.59	0.04	2.01	UR
95	MG(16:0)+H	MG(16:0)+H	MG	(16:0)	(16:0)		331.28	331.28	1.06	C19 H39 O4	1.24	0.02	1.78	UR
96	MG(16:0)+H	MG(16:0)+H	MG	(16:0)	(16:0)		331.28	331.28	5.869	C19 H39 O4	2.19	0.01	1.51	UR
97	MG(16:0)+H	MG(16:0)+H	MG	(16:0)	(16:0)		331.28	331.28	14.71	C19 H39 O4	2.36	0.00	1.65	UR
98	MG(16:2e)+H	MG(16:2e)+H	MG	(16:2e)	(16:2e)		313.27	313.27	5.433	C19 H37 O3	-1.18	0.02	1.86	DR
99	MG(16:2e)+H	MG(16:2e)+H	MG	(16:2e)	(16:2e)		313.27	313.27	12.02	C19 H37 O3	2.20	0.00	1.97	UR
100	MG(16:2e)+H	MG(16:2e)+H	MG	(16:2e)	(16:2e)		313.27	313.27	14.74	C19 H37 O3	-1.09	0.02	1.85	DR

101	MG(18:2+4O)+H	MG(18:2+4O)+H	MG	(18:2+4O)	(18:2+4O)		419.26	419.26	5.778	C21 H39 O8	-1.02	0.04	1.72	DR
102	MG(18:2+4O)+H	MG(18:2+4O)+H	MG	(18:2+4O)	(18:2+4O)		419.26	419.26	9.157	C21 H39 O8	2.37	0.01	1.91	UR
103	MG(19:1)+H	MG(19:1)+H	MG	(19:1)	(19:1)		371.32	371.32	9.938	C22 H43 O4	2.27	0.04	1.58	UR
104	MG(23:1)+NH4	MG(23:1)+NH4	MG	(23:1)	(23:1)		444.4	444.4	1.104	C26 H54 O4 N1	2.21	0.00	1.91	UR
105	MG(23:1)+Na	MG(23:1)+Na	MG	(23:1)	(23:1)		449.36	449.36	8.394	C26 H50 O4 Na1	-4.27	0.00	2.10	DR
106	MG(25:1)+H	MG(25:1)+H	MG	(25:1)	(25:1)		455.41	455.41	11.88	C28 H55 O4	-1.12	0.02	1.86	DR
107	MG(29:1)+NH4	MG(29:1)+NH4	MG	(29:1)	(29:1)		528.5	528.5	4.194	C32 H66 O4 N1	-1.12	0.04	1.68	DR
108	MG(29:1)+Na	MG(29:1)+Na	MG	(29:1)	(29:1)		533.45	533.45	5.896	C32 H62 O4 Na1	4.80	0.00	1.90	UR
109	MG(29:1)+H	MG(29:1)+H	MG	(29:1)	(29:1)		511.47	511.47	9.609	C32 H63 O4	2.42	0.03	1.84	UR
110	PA(14:2COOH)+Na	PA(14:2COOH)+Na	PA	(14:2COOH)	(14:2COOH)		445.12	445.12	13.59	C17 H27 O10 N0 P1 Na1	-1.56	0.04	1.56	DR
111	PA(15:3)+Na	PA(15:3)+Na	PA	(15:3)	(15:3)		427.15	427.15	8.408	C18 H29 O8 N0 P1 Na1	1.07	0.01	1.81	UR
112	PA(30:9+6O)+Na	PA(30:9+6O)+Na	PA	(30:9+6O)	(30:9+6O)		721.26	721.26	1.173	C33 H47 O14 N0 P1 Na1	2.16	0.05	2.02	UR
113	PA(37:1)+NH4	PA(37:1)+NH4	PA	(37:1)	(37:1)		734.57	734.57	9.608	C40 H81 O8 N1 P1	-2.42	0.00	2.01	DR
114	PA(40:2)+NH4	PA(40:2)+NH4	PA	(40:2)	(40:2)		774.6	774.6	9.956	C43 H85 O8 N1 P1	-3.60	0.00	2.00	DR
115	PA(41:6+7O)+Na	PA(41:6+7O)+Na	PA	(41:6+7O)	(41:6+7O)		897.47	897.47	12.43	C44 H75 O15 N0 P1 Na1	2.97	0.03	1.98	UR
116	PC(11:0_21:0)+H	PC(32:0)+H	PC	(11:0_21:0)	(11:0)	(21:0)	734.57	734.57	9.608	C40 H81 O8 N1 P1	-2.58	0.00	1.88	DR
117	PC(17:1_17:1)+Na	PC(34:2)+Na	PC	(17:1_17:1)	(17:1)	(17:1)	780.55	780.55	9.147	C42 H80 O8 N1 P1 Na1	-1.26	0.04	1.55	DR
118	PC(22:2_14:3)+H	PC(36:5)+H	PC	(22:2_14:3)	(22:2)	(14:3)	780.55	780.55	9.145	C44 H79 O8 N1 P1	-1.26	0.04	1.55	DR
119	PC(25:1_12:2)+H	PC(37:3)+H	PC	(25:1_12:2)	(25:1)	(12:2)	798.6	798.6	9.382	C45 H85 O8 N1 P1	-3.73	0.00	1.99	DR
120	PE(27:0_8:0)+H	PE(35:0)+H	PE	(27:0_8:0)	(27:0)	(8:0)	734.57	734.57	9.601	C40 H81 O8 N1 P1	-3.77	0.00	2.05	DR
121	PE(36:2)+H	PE(36:2)+H	PE	(36:2)	(36:2)		744.55	744.55	7.986	C41 H79 O8 N1 P1	2.80	0.02	1.88	UR
122	PE(37:3+OO)+H	PE(37:3+OO)+H	PE	(37:3+OO)	(37:3+OO)		788.54	788.54	7.55	C42 H79 O10 N1 P1	-1.12	0.00	1.98	DR
123	PE(18:0_20:1)+Na	PE(38:1)+Na	PE	(18:0_20:1)	(18:0)	(20:1)	796.58	796.58	9.016	C43 H84 O8 N1 P1 Na1	3.24	0.00	2.08	UR
124	PE(40:4)+H	PE(40:4)+H	PE	(40:4)	(40:4)		796.59	796.58	9.017	C45 H83 O8 N1 P1	3.24	0.00	2.08	UR
125	PEt(34:3)+H	PEt(34:3)+H	PEt	(34:3)	(34:3)		699.5	699.5	8.351	C39 H72 O8 N0 P1	-1.06	0.05	1.84	DR
126	PEt(42:4)+Na	PEt(42:4)+Na	PEt	(42:4)	(42:4)		831.59	831.58	8.58	C47 H85 O8 N0 P1 Na1	-1.30	0.02	1.94	DR
127	PG(5:0COOH_11:4)+Na	PG(16:4COOH)+Na	PG	(5:0COOH_11:4)	(5:0COOH)	(11:4)	543.16	543.16	1.015	C22 H33 O12 N0 P1 Na1	3.00	0.02	1.65	UR
128	PG(22:1_14:3)+NH4	PG(36:4)+NH4	PG	(22:1_14:3)	(22:1)	(14:3)	788.54	788.54	7.547	C42 H79 O10 N1 P1	-1.12	0.00	1.99	DR
129	PG(23:0_23:0)+H	PG(46:0)+H	PG	(23:0_23:0)	(23:0)	(23:0)	919.74	919.73	11.43	C52 H104 O10 N0 P1	-3.14	0.00	1.96	DR
130	PI(24:7CHO)+Na	PI(24:7CHO)+Na	PI	(24:7CHO)	(24:7CHO)		721.26	721.26	1.172	C33 H47 O14 N0 P1 Na1	2.16	0.05	2.02	UR
131	PI(18:0_13:0)+H	PI(31:0)+H	PI	(18:0_13:0)	(18:0)	(13:0)	797.52	797.52	8.091	C40 H78 O13 N0 P1	2.12	0.03	1.82	UR

132	PIP(26:5e)+Na	PIP(26:5e)+Na	PIP	(26:5e)	(26:5e)		805.33	805.33	0.699	C35 H60 O15 N0 P2 Na1	-3.52	0.04	2.01	DR
133	PIP(32:2e)+Na	PIP(32:2e)+Na	PIP	(32:2e)	(32:2e)		895.47	895.47	12.17	C41 H78 O15 N0 P2 Na1	-1.14	0.05	1.62	DR
134	PMe(29:3e)+H	PMe(29:3e)+H	PMe	(29:3e)	(29:3e)		601.42	601.42	5.015	C33 H62 O7 N0 P1	-2.57	0.04	1.80	DR
135	PS(36:1)+H	PS(36:1)+H	PS	(36:1)	(36:1)		790.56	790.56	8.074	C42 H81 O10 N1 P1	-2.17	0.01	1.89	DR
136	SPH(d12:0)+H	SPH(d12:0)+H	SPH	(d12:0)	(d12:0)		218.21	218.21	3.067	C12 H28 O2 N1	-1.78	0.01	1.94	DR
137	SPH(d12:0)+H	SPH(d12:0)+H	SPH	(d12:0)	(d12:0)		218.21	218.21	14.19	C12 H28 O2 N1	1.63	0.00	1.96	UR
138	SPH(d14:0)+H	SPH(d14:0)+H	SPH	(d14:0)	(d14:0)		246.24	246.24	0.666	C14 H32 O2 N1	-1.94	0.01	1.63	DR
139	SPH(t16:0)+H	SPH(t16:0)+H	SPH	(t16:0)	(t16:0)		290.27	290.27	15.32	C16 H36 O3 N1	-1.06	0.02	1.88	DR
140	SPH(t18:0)+H	SPH(t18:0)+H	SPH	(t18:0)	(t18:0)		318.3	318.3	6.382	C18 H40 O3 N1	1.76	0.05	1.79	UR
141	SPH(t18:0)+H	SPH(t18:0)+H	SPH	(t18:0)	(t18:0)		318.3	318.3	8.156	C18 H40 O3 N1	-1.13	0.03	1.90	DR
142	ST(d33:2+O)+NH4	ST(d33:2+O)+NH4	ST	(d33:2+O)	(d33:2+O)		797.52	797.52	8.091	C39 H77 O12 N2 S1	2.12	0.03	1.82	UR
143	ST(t33:2)+NH4	ST(t33:2)+NH4	ST	(t33:2)	(t33:2)		797.52	797.52	8.091	C39 H77 O12 N2 S1	2.12	0.03	1.82	UR
144	TG(6:0_6:0_10:1)+NH4	TG(22:1)+NH4	TG	(6:0_6:0_10:1)	(6:0)	(6:0)	458.35	458.35	3.509	C25 H48 O6 N1	2.01	0.00	1.87	UR
145	TG(6:0_12:2_18:3)+NH4	TG(36:5)+NH4	TG	(6:0_12:2_18:3)	(6:0)	(12:2)	646.5	646.5	7.68	C39 H68 O6 N1	-2.51	0.01	1.72	DR
146	TG(14:1e_11:2_11:2)+NH4	TG(36:5e)+NH4	TG	(14:1e_11:2_11:2)	(14:1e)	(11:2)	632.52	632.52	9.426	C39 H70 O5 N1	1.21	0.03	1.88	UR
147	TG(42:3COOCH3)+Na	TG(42:3COOCH3)+Na	TG	(42:3COOCH3)	(42:3COOCH3)		783.57	783.57	8.546	C46 H80 O8 Na1	-1.48	0.02	1.93	DR
148	TG(44:10e)+NH4	TG(44:10e)+NH4	TG	(44:10e)	(44:10e)		734.57	734.57	9.599	C47 H76 O5 N1	-4.73	0.00	2.05	DR
149	TG(46:11CHO)+NH4	TG(46:11CHO)+NH4	TG	(46:11CHO)	(46:11CHO)		788.55	788.54	7.55	C49 H74 O7 N1	-1.12	0.00	1.98	DR
150	TG(20:5_12:4_14:3)+NH4	TG(46:12)+NH4	TG	(20:5_12:4_14:3)	(20:5)	(12:4)	772.55	772.55	6.602	C49 H74 O6 N1	-1.65	0.01	1.95	DR
151	TG(16:0_16:1_20:1)+NH4	TG(52:2)+NH4	TG	(16:0_16:1_20:1)	(16:0)	(16:1)	876.8	876.8	7.941	C55 H106 O6 N1	3.36	0.00	1.94	UR
152	TG(52:3)+NH4	TG(52:3)+NH4	TG	(52:3)	(52:3)		874.79	874.79	9.361	C55 H104 O6 N1	2.38	0.05	1.94	UR
153	TG(10:0_18:1_24:2)+Na	TG(52:3)+Na	TG	(10:0_18:1_24:2)	(10:0)	(18:1)	879.74	879.74	11.31	C55 H100 O6 Na1	2.03	0.03	1.58	UR
154	TG(30:0_10:0_14:3)+NH4	TG(54:3)+NH4	TG	(30:0_10:0_14:3)	(30:0)	(10:0)	902.82	902.82	8.09	C57 H108 O6 N1	-1.20	0.05	1.67	DR
155	TG(30:1_10:3_14:0)+NH4	TG(54:4)+NH4	TG	(30:1_10:3_14:0)	(30:1)	(10:3)	900.8	900.8	6.948	C57 H106 O6 N1	-1.59	0.04	1.80	DR
156	TG(54:4+Ox)+Na	TG(54:4+Ox)+Na	TG	(54:4+Ox)	(54:4+Ox)		919.74	919.74	11.3	C57 H100 O7 Na1	3.42	0.04	2.06	UR
157	TG(54:4CHO)+Na	TG(54:4CHO)+Na	TG	(13:2CHO_19:1_22:1)	(13:2CHO)	(19:1)	919.74	919.74	11.44	C57 H100 O7 Na1	-3.13	0.00	2.08	DR
158	TG(54:5+O)+Na	TG(54:5+O)+Na	TG	(18:3+O_18:1_18:1)	(18:3+O)	(18:1)	919.74	919.73	11.43	C57 H100 O7 Na1	-3.14	0.00	1.96	DR
159	TG(18:3+O_18:2_18:2)+Na	TG(54:7+O)+Na	TG	(18:3+O_18:2_18:2)	(18:3+O)	(18:2)	915.7	915.7	11.06	C57 H96 O7 Na1	2.18	0.01	1.78	UR
160	TG(10:2CHO_22:0_22:6)+NH4	TG(54:8CHO)+NH4	TG	(10:2CHO_22:0_22:6)	(10:2CHO)	(22:0)	906.72	906.72	10.76	C57 H96 O7 N1	1.82	0.03	1.62	UR
161	TG(54:8CHO)+NH4	TG(54:8CHO)+NH4	TG	(54:8CHO)	(54:8CHO)		906.72	906.72	10.94	C57 H96 O7 N1	3.01	0.00	1.71	UR
162	TG(20:1_14:1_22:5)+H	TG(56:7)+H	TG	(20:1_14:1_22:5)	(20:1)	(14:1)	905.76	905.76	13.32	C59 H101 O6	2.24	0.04	1.74	UR
163	TG(58:12e)+H	TG(58:12e)+H	TG	(58:12e)	(58:12e)		909.73	909.73	11.06	C61 H97 O5	2.07	0.00	2.07	UR
164	WE(14:0)+NH4	WE(14:0)+NH4	WE	(14:0)	(14:0)		246.24	246.24	0.666	H32 C14 O2 N1	-1.94	0.01	1.63	DR
165	WE(18:3)+H	WE(18:3)+H	WE	(18:3)	(18:3)		279.23	279.23	3.644	H31 C18 O2	-1.74	0.04	1.88	DR
166	WE(20:0)+NH4	WE(20:0)+NH4	WE	(20:0)	(20:0)		330.34	330.34	4.747	H44 C20 O2 N1	-1.14	0.01	1.95	DR

167	WE(5:0_16:2)+H	WE(21:2)+H	WE	(5:0_16:2)	(5:0)	(16:2)	323.29	323.29	11.8	H39 C21 O2	2.22	0.04	1.62	UR
168	WE(47:8)+NH4	WE(47:8)+NH4	WE	(47:8)	(47:8)		692.63	692.63	11.73	H82 C47 O2 N1	2.67	0.01	2.00	UR
169	dMePE(35:3+OO)+H	dMePE(35:3+OO)+H	dMePE	(35:3+OO)	(35:3+OO)		788.54	788.54	7.547	C42 H79 O10 N1 P1	-1.12	0.00	1.99	DR
170	dMePE(38:3)+H	dMePE(38:3)+H	dMePE	(38:3)	(38:3)		798.6	798.6	9.365	C45 H85 O8 N1 P1	-4.09	0.00	2.02	DR

DR=down regulation, UR=upregulation, VIP=Variable importance projection

Table S5.Differentially accumulated lipids in seeds of PI518255 compared to PI598080 under heat stress

Lipid Ion	Lipid Group	Class	Fatty Acid	FA1	FA2	FA3	CalcMz	ObsMz	Rt	IonFormula	Log2(FC)	T-Test	VIP	Regulation
BiotinylPE(14:2_16:0)-H	BiotinylPE(30:2)-H	BiotinylPE	(14:2_16:0)	(14:2)	(16:0)		884.52	884.52	8.481	C45 H79 O10 N3 S1 P1	-2.1	0.03	1.83	DR
Cer(t18:1_27:0+O)+HCOO	Cer(t45:1+O)+HCOO	Cer	(t18:1_27:0+O)	(t18:1)	(27:0+O)		768.67	768.67	11.64	C46 H90 O7 N1	-1.3	0.01	1.85	DR
CerP(d32:3)-H	CerP(d32:3)-H	CerP	(d32:3)	(d32:3)			584.41	584.41	6.224	C32 H59 O6 N1 P1	1.61	0.02	1.74	UR
DGMG(33:4)+HCOO	DGMG(33:4)+HCOO	DGMG	(33:4)	(33:4)			929.58	929.58	6.179	C49 H85 O16	1.28	0.04	1.66	UR
DGMG(36:5)+HCOO	DGMG(36:5)+HCOO	DGMG	(36:5)	(36:5)			969.62	969.61	8.436	C52 H89 O16	-1.4	0.03	2.07	DR
Hex1SPH(m17:0)-H	Hex1SPH(m17:0)-H	Hex1SPH	(m17:0)	(m17:0)			432.33	432.33	6.798	C23 H46 O6 N1	-2.1	0.02	1.53	DR
Hex2Cer(m34:0)-H	Hex2Cer(m34:0)-H	Hex2Cer	(m34:0)	(m34:0)			846.63	846.63	10.02	C46 H88 O12 N1	-1.7	0.04	1.96	DR
LPA(18:1)-H	LPA(18:1)-H	LPA	(18:1)	(18:1)			435.25	435.25	2.748	C21 H40 O7 N0 P1	1.6	0.03	1.99	UR
LPC(12:0)+HCOO	LPC(12:0)+HCOO	LPC	(12:0)	(12:0)			484.27	484.27	14.34	C21 H43 O9 N1 P1	2.66	0.05	2.09	UR
LPG(18:1)-H	LPG(18:1)-H	LPG	(18:1)	(18:1)			509.29	509.29	2.41	C24 H46 O9 N0 P1	2.02	0.01	2.09	UR
LPI(16:0)-H	LPI(16:0)-H	LPI	(16:0)	(16:0)			571.29	571.29	2.159	C25 H48 O12 N0 P1	1.19	0.03	1.83	UR
LPI(8:2CHO)-H	LPI(8:2CHO)-H	LPI	(8:2CHO)	(8:2CHO)			469.11	469.11	0.74	C17 H26 O13 N0 P1	-4.3	0.01	2.04	DR
MGDG(19:1_12:0)-H	MGDG(31:1)-H	MGDG	(19:1_12:0)	(19:1)	(12:0)		713.52	713.52	6.512	C40 H73 O10	1.78	0.00	2.23	UR
MGDG(34:5)+HCOO	MGDG(34:5)+HCOO	MGDG	(34:5)	(34:5)			793.51	793.51	8.106	C44 H73 O12	1.89	0.00	2.13	UR
MGDG(38:3e)-H	MGDG(38:3e)-H	MGDG	(38:3e)	(38:3e)			793.62	793.62	11.86	C47 H85 O9	-1.6	0.00	2.08	DR
MGDG(45:14e)-H	MGDG(45:14e)-H	MGDG	(45:14e)	(45:14e)			869.56	869.56	11.08	C54 H77 O9	-4.2	0.05	1.81	DR
MGDG(45:6e)-H	MGDG(45:6e)-H	MGDG	(45:6e)	(45:6e)			885.68	885.68	11.86	C54 H93 O9	3.19	0.04	2.19	UR
MGDG(48:6e)+HCOO	MGDG(48:6e)+HCOO	MGDG	(48:6e)	(48:6e)			973.73	973.73	10.03	C58 H101 O11	1.95	0.05	1.87	UR

MGDG(49:4e)-H	MGDG(49:4e)-H	MGDG	(49:4e)	(49:4e)		945.78	945.78	12.48	C58 H105 O9	1.69	0.02	2.02	UR
MGDG(23:0_26:6)-H	MGDG(49:6)-H	MGDG	(23:0_26:6)	(23:0)	(26:6)	955.72	955.72	10.19	C58 H99 O10	-1.7	0.02	2.02	DR
MGDG(49:7e)-H	MGDG(49:7e)-H	MGDG	(49:7e)	(49:7e)		939.73	939.73	11.14	C58 H99 O9	-1.3	0.00	2.14	DR
MGMG(36:6)-H	MGMG(36:6)-H	MGMG	(36:6)	(36:6)		759.54	759.54	8.829	C45 H75 O9	-1.7	0.05	1.58	DR
OAHFA(12:0_12:0)-H	OAHFA(24:0)-H	OAHFA	(12:0_12:0)	(12:0)	(12:0)	397.33	397.33	3.835	C24 H45 O4	-3.7	0.00	2.21	DR
OAHFA(26:0)-H	OAHFA(26:0)-H	OAHFA	(26:0)	(26:0)		425.36	425.36	4.702	C26 H49 O4	-7.7	0.03	2.23	DR
OAHFA(12:0_14:0)-H	OAHFA(26:0)-H	OAHFA	(12:0_14:0)	(12:0)	(14:0)	425.36	425.36	5.12	C26 H49 O4	-3.3	0.00	2.22	DR
OAHFA(48:6)-H	OAHFA(48:6)-H	OAHFA	(48:6)	(48:6)		721.61	721.61	6.257	C48 H81 O4	-1	0.02	1.93	DR
OAHFA(61:12)-H	OAHFA(61:12)-H	OAHFA	(61:12)	(61:12)		891.72	891.72	13.07	C61 H95 O4	-1.3	0.01	1.88	DR
PA(8:1e_10:0)-H	PA(18:1e)-H	PA	(8:1e_10:0)	(8:1e)	(10:0)	435.25	435.25	2.604	C21 H40 O7 N0 P1	1.51	0.03	1.97	UR
PA(18:3e_13:0)-H	PA(31:3e)-H	PA	(18:3e_13:0)	(18:3e)	(13:0)	613.42	613.42	8.167	C34 H62 O7 N0 P1	-1.2	0.02	1.81	DR
PA(34:3+OO)-H	PA(34:3+OO)-H	PA	(34:3+OO)	(34:3+OO)		701.44	701.44	4.14	C37 H66 O10 N0 P1	-1.7	0.00	1.80	DR
PA(20:4e_24:0)-H	PA(44:4e)-H	PA	(20:4e_24:0)	(20:4e)	(24:0)	793.61	793.61	11.86	C47 H86 O7 N0 P1	-1.6	0.00	2.10	DR
PA(38:1_11:4)-H	PA(49:5)-H	PA	(38:1_11:4)	(38:1)	(11:4)	875.65	875.65	11.69	C52 H92 O8 N0 P1	1.74	0.02	2.02	UR
PA(38:1_11:4)-H	PA(49:5)-H	PA	(38:1_11:4)	(38:1)	(11:4)	875.65	875.65	11.88	C52 H92 O8 N0 P1	-2.2	0.00	1.94	DR
PA(27:0_22:6)-H	PA(49:6)-H	PA	(27:0_22:6)	(27:0)	(22:6)	873.64	873.64	11.56	C52 H90 O8 N0 P1	-1.4	0.01	2.00	DR
PC(29:2CHO)+HCOO	PC(29:2CHO)+HCOO	PC	(29:2CHO)	(29:2CHO)		746.46	746.46	5.153	C38 H69 O11 N1 P1	-3.7	0.01	1.92	DR
PC(16:0_18:2)+HCOO	PC(34:2)+HCOO	PC	(16:0_18:2)	(16:0)	(18:2)	802.56	802.56	9.105	C43 H81 O10 N1 P1	-1.1	0.03	1.93	DR
PC(20:0_18:2)+HCOO	PC(38:2)+HCOO	PC	(20:0_18:2)	(20:0)	(18:2)	858.62	858.62	10.35	C47 H89 O10 N1 P1	1.11	0.05	1.85	UR
PC(39:1+2O)+HCOO	PC(39:1+2O)+HCOO	PC	(39:1+2O)	(39:1+2O)		906.64	906.64	9.527	C48 H93 O12 N1 P1	2.21	0.01	1.73	UR
PE(8:2CHO_12:4)-H	PE(20:6CHO)-H	PE	(8:2CHO_12:4)	(8:2CHO)	(12:4)	524.21	524.21	0.688	C25 H35 O9 N1 P1	-2.8	0.05	1.88	DR
PE(17:1_18:2)-H	PE(35:3)-H	PE	(17:1_18:2)	(17:1)	(18:2)	726.51	726.51	9.074	C40 H73 O8 N1 P1	-2	0.01	1.91	DR
PE(19:1_18:1)-H	PE(37:2)-H	PE	(19:1_18:1)	(19:1)	(18:1)	756.55	756.55	10.22	C42 H79 O8 N1 P1	-3.5	0.01	2.06	DR
PE(19:1_18:3)-H	PE(37:4)-H	PE	(19:1_18:3)	(19:1)	(18:3)	752.52	752.52	8.857	C42 H75 O8 N1 P1	-1.2	0.04	1.54	DR
PE(20:0_18:1)-H	PE(38:1)-H	PE	(20:0_18:1)	(20:0)	(18:1)	772.59	772.59	10.68	C43 H83 O8 N1 P1	1.53	0.05	1.88	UR
PE(32:1_8:0)-H	PE(40:1)-H	PE	(32:1_8:0)	(32:1)	(8:0)	800.62	800.62	11.11	C45 H87 O8 N1 P1	1.58	0.00	2.16	UR
PG(29:1)-H	PG(29:1)-H	PG	(29:1)	(29:1)		677.44	677.44	10.28	C35 H66 O10 N0 P1	-1	0.03	1.70	DR
PG(40:6+7O)-H	PG(40:6+7O)-H	PG	(40:6+7O)	(40:6+7O)		933.5	933.5	8.329	C46 H78 O17 N0 P1	1.65	0.02	1.92	UR
PI(18:1e)-H	PI(18:1e)-H	PI	(18:1e)	(18:1e)		597.3	597.3	2.177	C27 H50 O12 N0	2.51	0.02	2.04	UR

									P1				
PI(24:3+7O)-H	PI(24:3+7O)-H	PI	(24:3+7O)	(24:3+7O)		803.31	803.31	0.707	C33 H56 O20 N0 P1	-1.3	0.04	1.60	DR
PI(34:1)-H	PI(34:1)-H	PI	(34:1)	(34:1)		835.53	835.53	8.547	C43 H80 O13 N0 P1	1.06	0.05	1.80	UR
PI(18:0_18:1)-H	PI(36:1)-H	PI	(18:0_18:1)	(18:0)	(18:1)	863.57	863.57	8.923	C45 H84 O13 N0 P1	1.97	0.01	2.09	UR
PI(18:3+OO_19:1)-H	PI(37:4+OO)-H	PI	(18:3+OO_19:1)	(18:3+OO)	(19:1)	903.52	903.52	8.325	C46 H80 O15 N0 P1	1.29	0.05	1.85	UR
PIP(28:7)-H	PIP(28:7)-H	PIP	(28:7)	(28:7)		819.31	819.31	0.693	C37 H57 O16 N0 P2	-1.7	0.04	1.66	DR
PIP(18:2_11:4)-H	PIP(29:6)-H	PIP	(18:2_11:4)	(18:2)	(11:4)	835.34	835.34	0.702	C38 H61 O16 N0 P2	-2.4	0.02	1.88	DR
PIP2(20:2COOH)-H	PIP2(20:2COOH)-H	PIP2	(20:2COOH)	(20:2COOH)		827.21	827.21	8.514	C29 H50 O21 N0 P3	-1.3	0.01	1.91	DR
PMe(20:1)-H	PMe(20:1)-H	PMe	(20:1)	(20:1)		491.28	491.28	1.646	C24 H44 O8 N0 P1	-2.8	0.01	1.68	DR
PMe(29:0COOH)-H	PMe(29:0COOH)-H	PMe	(29:0COOH)	(29:0COOH)		649.41	649.41	9.733	C33 H62 O10 N0 P1	-1.3	0.03	1.53	DR
PMe(48:5)-H	PMe(48:5)-H	PMe	(48:5)	(48:5)		875.65	875.65	11.9	C52 H92 O8 N0 P1	-3	0.00	2.06	DR
PS(18:1_18:2)-H	PS(36:3)-H	PS	(18:1_18:2)	(18:1)	(18:2)	784.51	784.51	9.643	C42 H75 O10 N1 P1	-1.6	0.00	2.19	DR
PS(36:4+4O)-H	PS(36:4+4O)-H	PS	(36:4+4O)	(36:4+4O)		846.48	846.48	9.864	C42 H73 O14 N1 P1	2.36	0.01	1.93	UR
SL(40:10e)+HCOO	SL(40:10e)+HCOO	SL	(40:10e)	(40:10e)		933.5	933.5	8.328	C50 H77 O14 S1	1.74	0.01	1.93	UR
WE(23:0)-H	WE(23:0)-H	WE	(23:0)	(23:0)		353.34	353.34	9.433	H45 C23 O2	-1.5	0.02	1.77	DR
cPA(33:1)-H	cPA(33:1)-H	cPA	(33:1)	(33:1)		627.48	627.48	10.42	C36 H68 O6 N0 P1	-2.6	0.01	2.04	DR
dMePE(16:0_18:2)-H	dMePE(34:2)-H	dMePE	(16:0_18:2)	(16:0)	(18:2)	742.54	742.54	8.958	C41 H77 O8 N1 P1	-1	0.02	1.86	DR
dMePE(34:2+7O)-H	dMePE(34:2+7O)-H	dMePE	(34:2+7O)	(34:2+7O)		854.5	854.5	9.391	C41 H77 O15 N1 P1	1.7	0.04	1.95	UR
dMePE(44:12)-H	dMePE(44:12)-H	dMePE	(44:12)	(44:12)		862.54	862.54	9.455	C51 H77 O8 N1 P1	-1.9	0.02	1.84	DR
phSM(t41:7)+HCOO	phSM(t41:7)+HCOO	phSM	(t41:7)	(t41:7)		865.57	865.57	8.917	C47 H82 O10 N2 P1	1.95	0.00	2.05	UR
AcHexSiE()+NH4	AcHexSiE()+NH4	AcHexSiE	()			594.47	594.47	7.89	C35 H64 O6 N1	1.10	0.04	1.99	UR
AcHexZyE(20:0)+NH4	AcHexZyE(20:0)+NH4	AcHexZyE	(20:0)	(20:0)		858.72	858.72	11.69	C53 H96 O7 N1	1.74	0.02	2.07	UR
BiotinylPE(29:0)+H	BiotinylPE(29:0)+H	BiotinylPE	(29:0)	(29:0)		876.55	876.55	7.38	C44 H83 O10 N3 S1 P1	1.21	0.04	1.95	UR
BisMePA(27:1)+NH4	BisMePA(27:1)+NH4	BisMePA	(27:1)	(27:1)		622.44	622.44	13.61	C32 H65 O8 N1 P1	-2.00	0.00	2.23	DR
BisMePA(23:1_11:2)+NH4	BisMePA(34:3)+NH4	BisMePA	(23:1_11:2)	(23:1)	(11:2)	716.52	716.52	8.33	C39 H75 O8 N1 P1	1.01	0.03	1.83	UR
BisMePA(37:3)+NH4	BisMePA(37:3)+NH4	BisMePA	(37:3)	(37:3)		758.57	758.57	9.74	C42 H81 O8 N1 P1	-5.00	0.03	2.21	DR
BisMePA(37:3)+NH4	BisMePA(37:3)+NH4	BisMePA	(37:3)	(37:3)		758.57	758.57	12.40	C42 H81 O8 N1 P1	-3.67	0.00	1.95	DR

BisMePA(39:5)+NH4	BisMePA(39:5)+NH4	BisMePA	(39:5)	(39:5)		782.57	782.57	13.60	C44 H81 O8 N1 P1	-2.02	0.00	2.17	DR
BisMePA(39:6)+NH4	BisMePA(39:6)+NH4	BisMePA	(39:6)	(39:6)		780.55	780.55	9.28	C44 H79 O8 N1 P1	-1.48	0.01	1.96	DR
BisMePA(34:0_14:0)+Na	BisMePA(48:0)+Na	BisMePA	(34:0_14:0)	(34:0)	(14:0)	923.74	923.74	13.40	C53 H105 O8 N0 P1 Na1	1.23	0.01	2.06	UR
BisMePE(32:2)+H	BisMePE(32:2)+H	BisMePE	(32:2)	(32:2)		716.52	716.52	8.33	C39 H75 O8 N1 P1	1.01	0.03	1.83	UR
BisMePE(34:1)+H	BisMePE(34:1)+H	BisMePE	(34:1)	(34:1)		746.57	746.57	10.21	C41 H81 O8 N1 P1	1.50	0.05	1.80	UR
BisMePS(42:1)+NH4	BisMePS(42:1)+NH4	BisMePS	(42:1)	(42:1)		919.71	919.71	13.72	C50 H100 O10 N2 P1	4.84	0.03	2.09	UR
Cer(d26:0+O)+H	Cer(d26:0+O)+H	Cer	(d26:0+O)	(d26:0+O)		444.40	444.41	2.74	C26 H54 O4 N1	1.25	0.00	1.97	UR
Cer(d26:0+O)+H	Cer(d26:0+O)+H	Cer	(d26:0+O)	(d26:0+O)		444.40	444.40	9.78	C26 H54 O4 N1	-1.59	0.01	2.16	DR
Cer(d26:0+O)+H	Cer(d26:0+O)+H	Cer	(d26:0+O)	(d26:0+O)		444.40	444.40	14.12	C26 H54 O4 N1	-2.19	0.03	1.77	DR
Cer(d14:0_14:0)+H	Cer(d28:0)+H	Cer	(d14:0_14:0)	(d14:0)	(14:0)	456.44	456.44	9.12	C28 H58 O3 N1	2.76	0.04	2.20	UR
Cer(d28:2+O)+NH4	Cer(d28:2+O)+NH4	Cer	(d28:2+O)	(d28:2+O)		485.43	485.43	11.61	C28 H57 O4 N2	-1.58	0.00	2.14	DR
Cer(d17:0_15:0)+H	Cer(d32:0)+H	Cer	(d17:0_15:0)	(d17:0)	(15:0)	512.50	512.50	10.25	C32 H66 O3 N1	1.12	0.01	2.14	UR
Cer(d32:0+O)+H	Cer(d32:0+O)+H	Cer	(d32:0+O)	(d32:0+O)		528.50	528.50	8.63	C32 H66 O4 N1	1.96	0.05	1.83	UR
Cer(d32:0+O)+H	Cer(d32:0+O)+H	Cer	(d32:0+O)	(d32:0+O)		528.50	528.50	11.15	C32 H66 O4 N1	-2.33	0.02	2.04	DR
Cer(d37:0+2O)+Na	Cer(d37:0+2O)+Na	Cer	(d37:0+2O)	(d37:0+2O)		636.55	636.55	10.48	C37 H75 O5 N1 Na1	1.87	0.02	1.88	UR
Cer(d21:1_18:1+O)+H	Cer(d39:2+O)+H	Cer	(d21:1_18:1+O)	(d21:1)	(18:1+O)	622.58	622.58	11.59	C39 H76 O4 N1	2.75	0.02	2.22	UR
Cer(d23:2_16:0+O)+H	Cer(d39:2+O)+H	Cer	(d23:2_16:0+O)	(d23:2)	(16:0+O)	622.58	622.58	11.79	C39 H76 O4 N1	-4.50	0.00	2.24	DR
Cer(d21:1_18:2+2O)+H	Cer(d39:3+2O)+H	Cer	(d21:1_18:2+2O)	(d21:1)	(18:2+2O)	636.56	636.55	10.48	C39 H74 O5 N1	1.87	0.02	1.88	UR
Cer(m32:0+O)+H	Cer(m32:0+O)+H	Cer	(m32:0+O)	(m32:0+O)		512.50	512.50	10.25	C32 H66 O3 N1	1.12	0.01	2.14	UR
Cer(m32:0+O)+H	Cer(m32:0+O)+H	Cer	(m32:0+O)	(m32:0+O)		512.50	512.50	12.08	C32 H66 O3 N1	-3.17	0.02	2.07	DR
Cer(m34:0+O)+H	Cer(m34:0+O)+H	Cer	(m34:0+O)	(m34:0+O)		540.54	540.54	12.77	C34 H70 O3 N1	1.51	0.03	1.92	UR
Cer(m39:2+2O)+H	Cer(m39:2+2O)+H	Cer	(m39:2+2O)	(m39:2+2O)		622.58	622.58	11.79	C39 H76 O4 N1	-4.50	0.00	2.24	DR
Cer(t32:0)+H	Cer(t32:0)+H	Cer	(t32:0)	(t32:0)		528.50	528.50	11.15	C32 H66 O4 N1	-2.33	0.02	2.04	DR
Cer(t18:0_19:0+O)+Na	Cer(t37:0+O)+Na	Cer	(t18:0_19:0+O)	(t18:0)	(19:0+O)	636.55	636.55	10.48	C37 H75 O5 N1 Na1	1.87	0.02	1.88	UR
Cer(t16:1_23:1)+H	Cer(t39:2)+H	Cer	(t16:1_23:1)	(t16:1)	(23:1)	622.58	622.58	11.59	C39 H76 O4 N1	2.75	0.02	2.22	UR
Cer(t18:1_21:1)+H	Cer(t39:2)+H	Cer	(t18:1_21:1)	(t18:1)	(21:1)	622.58	622.58	11.79	C39 H76 O4 N1	-4.52	0.00	2.24	DR
Cer(t60:0EO)+Na	Cer(t60:0EO)+Na	Cer	(t60:0EO)	(t60:0EO)		972.89	972.89	13.23	C60 H119 O6 N1 Na1	1.24	0.01	2.14	UR
CerP(d21:0_16:0+2O)+Na	CerP(d37:0+2O)+Na	CerP	(d21:0_16:0+2O)	(d21:0)	(16:0+2O)	716.52	716.52	8.33	C37 H76 O8 N1 P1 Na1	1.01	0.03	1.83	UR
CerP(d39:3+2O)+H	CerP(d39:3+2O)+H	CerP	(d39:3+2O)	(d39:3+2O)		716.52	716.52	8.33	C39 H75 O8 N1 P1	1.01	0.03	1.83	UR

CerP(d42:3+2O)+Na	CerP(d42:3+2O)+Na	CerP	(d42:3+2O)	(d42:3+2O)		780.55	780.55	8.97	C42 H80 O8 N1 P1 Na1	-1.08	0.03	1.64	DR
CerP(d22:2_25:0+O)+NH4	CerP(d47:2+O)+NH4	CerP	(d22:2_25:0+O)	(d22:2)	(25:0+O)	831.69	831.69	11.55	C47 H96 O7 N2 P1	-2.20	0.04	1.71	DR
CerP(t37:0+O)+Na	CerP(t37:0+O)+Na	CerP	(t37:0+O)	(t37:0+O)		716.52	716.52	8.33	C37 H76 O8 N1 P1 Na1	1.01	0.03	1.83	UR
DG(10:0)+Na	DG(10:0)+Na	DG	(10:0)	(10:0)		283.15	283.15	5.99	C13 H24 O5 Na1	-2.22	0.03	1.96	DR
DG(10:0)+Na	DG(10:0)+Na	DG	(10:0)	(10:0)		283.15	283.15	6.60	C13 H24 O5 Na1	2.17	0.03	1.79	DR
DG(10:0)+Na	DG(10:0)+Na	DG	(10:0)	(10:0)		283.15	283.15	9.14	C13 H24 O5 Na1	-2.40	0.05	1.84	DR
DG(10:0)+Na	DG(10:0)+Na	DG	(10:0)	(10:0)		283.15	283.15	9.97	C13 H24 O5 Na1	-1.05	0.02	1.97	DR
DG(10:0)+Na	DG(10:0)+Na	DG	(10:0)	(10:0)		283.15	283.15	11.80	C13 H24 O5 Na1	2.03	0.02	1.87	DR
DG(10:0)+Na	DG(10:0)+Na	DG	(10:0)	(10:0)		283.15	283.15	11.95	C13 H24 O5 Na1	-1.47	0.03	2.07	DR
DG(13:0CHO)+H	DG(13:0CHO)+H	DG	(13:0CHO)	(13:0CHO)		317.20	317.20	4.79	C16 H29 O6	-1.02	0.00	2.18	DR
DG(16:0e)+H	DG(16:0e)+H	DG	(16:0e)	(16:0e)		331.28	331.28	3.97	C19 H39 O4	-1.06	0.05	1.85	DR
DG(16:0e)+NH4	DG(16:0e)+NH4	DG	(16:0e)	(16:0e)		348.31	348.31	4.53	C19 H42 O4 N1	-1.54	0.04	1.75	DR
DG(16:0e)+H	DG(16:0e)+H	DG	(16:0e)	(16:0e)		331.28	331.28	13.28	C19 H39 O4	-1.19	0.03	1.84	DR
DG(18:1e)+H	DG(18:1e)+H	DG	(18:1e)	(18:1e)		357.30	357.30	10.73	C21 H41 O4	-1.44	0.02	1.83	DR
DG(19:1)+NH4	DG(19:1)+NH4	DG	(19:1)	(19:1)		402.32	402.32	4.49	C22 H44 O5 N1	-1.37	0.00	2.14	DR
DG(19:1e)+H	DG(19:1e)+H	DG	(19:1e)	(19:1e)		371.32	371.32	7.51	C22 H43 O4	-1.50	0.03	1.81	DR
DG(19:1e)+H	DG(19:1e)+H	DG	(19:1e)	(19:1e)		371.32	371.32	9.94	C22 H43 O4	-1.65	0.04	2.03	DR
DG(19:1e)+H	DG(19:1e)+H	DG	(19:1e)	(19:1e)		371.32	371.32	12.07	C22 H43 O4	-2.29	0.00	2.12	DR
DG(19:1e)+H	DG(19:1e)+H	DG	(19:1e)	(19:1e)		371.32	371.32	14.61	C22 H43 O4	-3.30	0.01	1.81	DR
DG(21:1)+NH4	DG(21:1)+NH4	DG	(21:1)	(21:1)		430.35	430.35	7.79	C24 H48 O5 N1	-2.04	0.02	2.04	DR
DG(21:1)+NH4	DG(21:1)+NH4	DG	(21:1)	(21:1)		430.35	430.35	8.93	C24 H48 O5 N1	-2.77	0.00	1.67	DR
DG(21:1e)+NH4	DG(21:1e)+NH4	DG	(21:1e)	(21:1e)		416.37	416.37	8.07	C24 H50 O4 N1	2.11	0.00	2.08	UR
DG(21:5e)+H	DG(21:5e)+H	DG	(21:5e)	(21:5e)		391.28	391.28	6.79	C24 H39 O4	-1.29	0.01	2.05	DR
DG(21:5e)+H	DG(21:5e)+H	DG	(21:5e)	(21:5e)		391.28	391.28	8.08	C24 H39 O4	-2.08	0.04	1.88	DR
DG(21:5e)+H	DG(21:5e)+H	DG	(21:5e)	(21:5e)		391.28	391.28	13.49	C24 H39 O4	-1.51	0.05	1.75	DR
DG(21:5e)+H	DG(21:5e)+H	DG	(21:5e)	(21:5e)		391.28	391.28	14.90	C24 H39 O4	1.67	0.03	1.88	DR
DG(21:5e)+H	DG(21:5e)+H	DG	(21:5e)	(21:5e)		391.28	391.28	15.58	C24 H39 O4	-1.38	0.04	1.85	DR
DG(21:7CHO)+H	DG(21:7CHO)+H	DG	(21:7CHO)	(21:7CHO)		415.21	415.21	1.95	C24 H31 O6	-1.01	0.00	2.20	DR
DG(21:7CHO)+H	DG(21:7CHO)+H	DG	(21:7CHO)	(21:7CHO)		415.21	415.21	4.88	C24 H31 O6	-2.07	0.02	1.89	DR
DG(22:1CHO)+NH4	DG(22:1CHO)+NH4	DG	(22:1CHO)	(22:1CHO)		458.35	458.35	4.18	C25 H48 O6 N1	-1.95	0.03	1.97	DR

DG(23:0COOH)+NH4	DG(23:0COOH)+NH4	DG	(23:0COOH)	(23:0COOH)		490.37	490.37	10.77	C26 H52 O7 N1	-2.03	0.00	1.90	DR
DG(23:1)+Na	DG(23:1)+Na	DG	(23:1)	(23:1)		463.34	463.34	5.46	C26 H48 O5 Na1	-2.90	0.00	1.81	DR
DG(23:1COOCH3)+NH4	DG(23:1COOCH3)+NH4	DG	(23:1COOCH3)	(23:1COOCH3)		502.37	502.37	6.89	C27 H52 O7 N1	2.16	0.01	2.11	DR
DG(23:1COOCH3)+NH4	DG(23:1COOCH3)+NH4	DG	(23:1COOCH3)	(23:1COOCH3)		502.37	502.37	11.97	C27 H52 O7 N1	-1.47	0.01	2.00	DR
DG(12:0e_11:1)+NH4	DG(23:1e)+NH4	DG	(12:0e_11:1)	(12:0e)	(11:1)	444.40	444.40	2.73	C26 H54 O4 N1	-1.33	0.00	2.09	DR
DG(23:1e)+H	DG(23:1e)+H	DG	(23:1e)	(23:1e)		427.38	427.38	4.40	C26 H51 O4	-1.22	0.01	2.02	DR
DG(12:1e_11:0)+NH4	DG(23:1e)+NH4	DG	(12:1e_11:0)	(12:1e)	(11:0)	444.40	444.40	8.36	C26 H54 O4 N1	-1.85	0.00	2.22	DR
DG(23:1e)+NH4	DG(23:1e)+NH4	DG	(23:1e)	(23:1e)		444.40	444.40	14.02	C26 H54 O4 N1	1.38	0.04	1.68	UR
DG(23:1e)+H	DG(23:1e)+H	DG	(23:1e)	(23:1e)		427.38	427.38	15.15	C26 H51 O4	-1.34	0.01	1.98	DR
DG(23:2)+H	DG(23:2)+H	DG	(23:2)	(23:2)		439.34	439.34	7.50	C26 H47 O5	-1.97	0.01	1.63	DR
DG(24:1COOH)+NH4	DG(24:1COOH)+NH4	DG	(24:1COOH)	(24:1COOH)		502.37	502.37	11.94	C27 H52 O7 N1	-1.95	0.01	2.03	DR
DG(24:1COOH)+NH4	DG(24:1COOH)+NH4	DG	(24:1COOH)	(24:1COOH)		502.37	502.37	12.86	C27 H52 O7 N1	-1.60	0.02	2.10	DR
DG(24:1COOH)+NH4	DG(24:1COOH)+NH4	DG	(24:1COOH)	(24:1COOH)		502.37	502.37	13.07	C27 H52 O7 N1	-2.00	0.03	1.84	DR
DG(24:2+OO)+NH4	DG(24:2+OO)+NH4	DG	(24:2+OO)	(24:2+OO)		502.37	502.37	11.97	C27 H52 O7 N1	-1.25	0.03	1.90	DR
DG(24:2+OO)+NH4	DG(24:2+OO)+NH4	DG	(24:2+OO)	(24:2+OO)		502.37	502.37	12.90	C27 H52 O7 N1	-1.20	0.01	1.98	DR
DG(25:1e)+H	DG(25:1e)+H	DG	(25:1e)	(25:1e)		455.41	455.41	9.39	C28 H55 O4	-2.56	0.02	1.81	DR
DG(25:4)+H	DG(25:4)+H	DG	(25:4)	(25:4)		463.34	463.34	5.48	C28 H47 O5	-2.18	0.00	1.88	DR
DG(25:4e)+H	DG(25:4e)+H	DG	(25:4e)	(25:4e)		449.36	449.36	2.49	C28 H49 O4	-1.72	0.02	1.94	DR
DG(25:4e)+H	DG(25:4e)+H	DG	(25:4e)	(25:4e)		449.36	449.36	6.41	C28 H49 O4	-3.38	0.03	1.92	DR
DG(25:4e)+H	DG(25:4e)+H	DG	(25:4e)	(25:4e)		449.36	449.36	15.32	C28 H49 O4	-1.28	0.02	1.88	DR
DG(25:5e)+H	DG(25:5e)+H	DG	(25:5e)	(25:5e)		447.35	447.35	2.85	C28 H47 O4	-1.97	0.01	2.08	DR
DG(26:2+3O)+NH4	DG(26:2+3O)+NH4	DG	(26:2+3O)	(26:2+3O)		546.40	546.40	1.96	C29 H56 O8 N1	-1.34	0.04	1.87	DR
DG(26:2+3O)+NH4	DG(26:2+3O)+NH4	DG	(26:2+3O)	(26:2+3O)		546.40	546.40	8.63	C29 H56 O8 N1	1.25	0.04	1.67	UR
DG(10:0e_18:1)+NH4	DG(28:1e)+NH4	DG	(10:0e_18:1)	(10:0e)	(18:1)	514.48	514.48	4.62	C31 H64 O4 N1	-1.22	0.05	1.90	DR
DG(28:1e)+NH4	DG(28:1e)+NH4	DG	(28:1e)	(28:1e)		514.48	514.48	5.45	C31 H64 O4 N1	1.36	0.04	1.95	UR
DG(28:1e)+NH4	DG(28:1e)+NH4	DG	(28:1e)	(28:1e)		514.48	514.48	8.86	C31 H64 O4 N1	2.01	0.01	1.88	UR
DG(28:1e)+NH4	DG(28:1e)+NH4	DG	(28:1e)	(28:1e)		514.48	514.48	11.19	C31 H64 O4 N1	-1.15	0.03	1.88	DR
DG(28:1e)+H	DG(28:1e)+H	DG	(28:1e)	(28:1e)		497.46	497.46	14.41	C31 H61 O4	-1.99	0.00	2.17	DR
DG(28:2+4O)+NH4	DG(28:2+4O)+NH4	DG	(28:2+4O)	(28:2+4O)		590.43	590.43	9.49	C31 H60 O9 N1	-1.66	0.01	2.16	DR
DG(28:2+4O)+NH4	DG(28:2+4O)+NH4	DG	(28:2+4O)	(28:2+4O)		590.43	590.43	10.50	C31 H60 O9 N1	-2.07	0.01	1.99	DR
DG(12:1e_17:0)+NH4	DG(29:1e)+NH4	DG	(12:1e_17:0)	(12:1e)	(17:0)	528.50	528.50	1.75	C32 H66 O4 N1	-1.59	0.04	1.96	DR

DG(29:1e)+Na	DG(29:1e)+Na	DG	(29:1e)	(29:1e)		533.45	533.45	5.45	C32 H62 O4 Na1	1.89	0.00	1.97	UR
DG(29:1e)+H	DG(29:1e)+H	DG	(12:0e_17:1)	(12:0e)	(17:1)	511.47	511.47	6.15	C32 H63 O4	1.86	0.04	1.97	UR
DG(29:1e)+H	DG(29:1e)+H	DG	(29:1e)	(29:1e)		511.47	511.47	10.00	C32 H63 O4	1.43	0.00	2.08	UR
DG(29:1e)+NH4	DG(29:1e)+NH4	DG	(29:1e)	(29:1e)		528.50	528.50	10.69	C32 H66 O4 N1	-1.45	0.02	2.09	DR
DG(29:2+8O)+Na	DG(29:2+8O)+Na	DG	(29:2+8O)	(29:2+8O)		673.38	673.38	15.52	C32 H58 O13 Na1	1.35	0.04	1.82	UR
DG(30:2+5O)+NH4	DG(30:2+5O)+NH4	DG	(30:2+5O)	(30:2+5O)		634.45	634.45	6.56	C33 H64 O10 N1	1.04	0.03	1.82	UR
DG(30:2+5O)+NH4	DG(30:2+5O)+NH4	DG	(30:2+5O)	(30:2+5O)		634.45	634.45	12.47	C33 H64 O10 N1	-1.46	0.01	1.70	DR
DG(31:4e)+H	DG(31:4e)+H	DG	(31:4e)	(31:4e)		533.46	533.45	4.07	C34 H61 O4	-1.44	0.00	2.03	DR
DG(32:2+6O)+NH4	DG(32:2+6O)+NH4	DG	(32:2+6O)	(32:2+6O)		678.48	678.48	2.95	C35 H68 O11 N1	-1.58	0.02	1.96	DR
DG(32:2+6O)+NH4	DG(32:2+6O)+NH4	DG	(32:2+6O)	(32:2+6O)		678.48	678.48	4.68	C35 H68 O11 N1	1.81	0.04	1.81	UR
DG(32:2e)+H	DG(32:2e)+H	DG	(32:2e)	(32:2e)		551.50	551.50	10.82	C35 H67 O4	4.12	0.00	2.15	UR
DG(32:3CHO)+NH4	DG(32:3CHO)+NH4	DG	(32:3CHO)	(32:3CHO)		594.47	594.47	7.89	C35 H64 O6 N1	1.57	0.05	1.53	UR
DG(34:0)+NH4	DG(34:0)+NH4	DG	(34:0)	(34:0)		614.57	614.57	11.29	C37 H76 O5 N1	1.60	0.03	1.80	UR
DG(34:1)+Na	DG(34:1)+Na	DG	(34:1)	(34:1)		617.51	617.51	10.86	C37 H70 O5 Na1	1.13	0.05	1.51	UR
DG(34:2+3O)+Na	DG(34:2+3O)+Na	DG	(34:2+3O)	(34:2+3O)		663.48	663.48	6.33	C37 H68 O8 Na1	-2.55	0.04	1.67	DR
DG(34:2e)+NH4	DG(34:2e)+NH4	DG	(34:2e)	(34:2e)		596.56	596.56	12.94	C37 H74 O4 N1	-1.41	0.03	1.87	DR
DG(34:3e)+Na	DG(34:3e)+Na	DG	(34:3e)	(34:3e)		599.50	599.50	13.21	C37 H68 O4 Na1	1.75	0.00	2.13	UR
DG(34:4e)+H	DG(34:4e)+H	DG	(34:4e)	(34:4e)		575.50	575.50	12.96	C37 H67 O4	1.61	0.03	1.93	UR
DG(36:1)+NH4	DG(36:1)+NH4	DG	(36:1)	(36:1)		640.59	640.59	11.10	C39 H78 O5 N1	1.06	0.01	2.02	UR
DG(36:2)+NH4	DG(36:2)+NH4	DG	(36:2)	(36:2)		638.57	638.57	11.74	C39 H76 O5 N1	-1.08	0.03	2.05	DR
DG(18:1_18:1)+NH4	DG(36:2)+NH4	DG	(18:1_18:1)	(18:1)	(18:1)	638.57	638.57	12.79	C39 H76 O5 N1	2.11	0.05	1.98	UR
DG(36:3)+NH4	DG(36:3)+NH4	DG	(22:1_14:2)	(22:1)	(14:2)	636.56	636.55	10.48	C39 H74 O5 N1	1.87	0.02	1.88	UR
DG(36:3e)+NH4	DG(36:3e)+NH4	DG	(36:3e)	(36:3e)		622.58	622.58	11.59	C39 H76 O4 N1	2.75	0.02	2.22	UR
DG(36:3e)+NH4	DG(36:3e)+NH4	DG	(36:3e)	(36:3e)		622.58	622.58	11.79	C39 H76 O4 N1	-4.52	0.00	2.24	DR
DG(36:4e)+H	DG(36:4e)+H	DG	(36:4e)	(36:4e)		603.53	603.53	13.33	C39 H71 O4	1.38	0.00	2.21	UR
Hex1Cer(d18:2_16:0+O)+H	Hex1Cer(d34:2+O)+H	Hex1Cer	(d18:2_16:0+O)	(d18:2)	(16:0+O)	714.55	714.55	8.65	C40 H76 O9 N1	2.06	0.05	1.89	UR
Hex1Cer(d46:0)+H	Hex1Cer(d46:0)+H	Hex1Cer	(d46:0)	(d46:0)		870.78	870.78	11.93	C52 H104 O8 N1	1.56	0.05	1.77	UR
Hex1Cer(d48:3)+NH4	Hex1Cer(d48:3)+NH4	Hex1Cer	(d48:3)	(d48:3)		909.79	909.79	10.75	C54 H105 O8 N2	-2.33	0.03	1.87	DR
Hex1Cer(m45:2)+Na	Hex1Cer(m45:2)+Na	Hex1Cer	(m45:2)	(m45:2)		858.72	858.72	11.69	C51 H97 O7 N1 Na1	1.83	0.02	2.09	UR
Hex1Cer(m46:0+O)+H	Hex1Cer(m46:0+O)+H	Hex1Cer	(m46:0+O)	(m46:0+O)		870.78	870.77	9.42	C52 H104 O8 N1	2.75	0.04	2.06	UR

Hex1Cer(m20:0_26:0+O)+H	Hex1Cer(m46:0+O)+H	Hex1Cer	(m20:0_26:0+O)	(m20:0)	(26:0+O)	870.78	870.78	10.40	C52 H104 O8 N1	1.87	0.00	1.95	UR
Hex1SPH(d21:2)+H	Hex1SPH(d21:2)+H	Hex1SPH	(d21:2)	(d21:2)		502.37	502.37	5.36	C27 H52 O7 N1	-1.12	0.03	1.91	DR
Hex1SPH(d21:2)+H	Hex1SPH(d21:2)+H	Hex1SPH	(d21:2)	(d21:2)		502.37	502.37	13.08	C27 H52 O7 N1	-1.85	0.01	1.93	DR
Hex1SPH(m17:1)+NH4	Hex1SPH(m17:1)+NH4	Hex1SPH	(m17:1)	(m17:1)		449.36	449.36	2.49	C23 H49 O6 N2	-1.95	0.03	2.04	DR
Hex1SPH(m17:1)+NH4	Hex1SPH(m17:1)+NH4	Hex1SPH	(m17:1)	(m17:1)		449.36	449.36	3.95	C23 H49 O6 N2	-1.71	0.01	2.03	DR
Hex2Cer(d30:2)+H	Hex2Cer(d30:2)+H	Hex2Cer	(d30:2)	(d30:2)		804.55	804.55	8.80	C42 H78 O13 N1	-1.03	0.00	2.12	DR
LPE(15:0)+H	LPE(15:0)+H	LPE	(15:0)	(15:0)		440.28	440.28	15.63	C20 H43 O7 N1 P1	1.35	0.02	2.01	UR
LPI(13:2COOH)+H	LPI(13:2COOH)+H	LPI	(13:2COOH)	(13:2COOH)		557.20	557.20	0.97	C22 H38 O14 N0 P1	-3.44	0.04	1.65	DR
LPMe(20:1e)+H	LPMe(20:1e)+H	LPMe	(20:1e)	(20:1e)		465.33	465.33	9.14	C24 H50 O6 N0 P1	-1.26	0.04	1.95	DR
MG(16:0)+H	MG(16:0)+H	MG	(16:0)	(16:0)		331.28	331.28	0.28	C19 H39 O4	-1.72	0.04	1.94	DR
MG(16:0)+H	MG(16:0)+H	MG	(16:0)	(16:0)		331.28	331.28	2.41	C19 H39 O4	1.09	0.03	1.79	UR
MG(16:0)+NH4	MG(16:0)+NH4	MG	(16:0)	(16:0)		348.31	348.31	3.98	C19 H42 O4 N1	-2.04	0.01	1.84	DR
MG(16:0)+H	MG(16:0)+H	MG	(16:0)	(16:0)		331.28	331.28	5.40	C19 H39 O4	-1.09	0.03	1.71	DR
MG(16:0)+NH4	MG(16:0)+NH4	MG	(16:0)	(16:0)		348.31	348.31	5.87	C19 H42 O4 N1	-1.32	0.04	1.87	DR
MG(16:0)+NH4	MG(16:0)+NH4	MG	(16:0)	(16:0)		348.31	348.31	6.54	C19 H42 O4 N1	1.50	0.03	2.03	UR
MG(16:0)+H	MG(16:0)+H	MG	(16:0)	(16:0)		331.28	331.28	10.21	C19 H39 O4	1.01	0.02	2.07	UR
MG(16:2e)+H	MG(16:2e)+H	MG	(16:2e)	(16:2e)		313.27	313.27	12.02	C19 H37 O3	-1.98	0.02	2.13	DR
MG(16:2e)+H	MG(16:2e)+H	MG	(16:2e)	(16:2e)		313.27	313.27	15.22	C19 H37 O3	1.30	0.03	2.02	UR
MG(17:0)+NH4	MG(17:0)+NH4	MG	(17:0)	(17:0)		362.33	362.33	3.41	C20 H44 O4 N1	-1.60	0.03	2.08	DR
MG(18:0)+H	MG(18:0)+H	MG	(18:0)	(18:0)		359.32	359.32	0.13	C21 H43 O4	-1.64	0.01	1.98	DR
MG(18:0)+H	MG(18:0)+H	MG	(18:0)	(18:0)		359.32	359.32	1.28	C21 H43 O4	1.55	0.01	2.18	UR
MG(18:2+4O)+H	MG(18:2+4O)+H	MG	(18:2+4O)	(18:2+4O)		419.26	419.26	10.59	C21 H39 O8	1.25	0.00	2.18	UR
MG(18:2+4O)+H	MG(18:2+4O)+H	MG	(18:2+4O)	(18:2+4O)		419.26	419.26	14.68	C21 H39 O8	-2.29	0.01	1.83	DR
MG(18:3e)+H	MG(18:3e)+H	MG	(18:3e)	(18:3e)		339.29	339.29	10.89	C21 H39 O3	1.61	0.00	2.17	DR
MG(19:1)+H	MG(19:1)+H	MG	(19:1)	(19:1)		371.32	371.32	9.39	C22 H43 O4	-1.68	0.01	1.97	DR
MG(19:1)+H	MG(19:1)+H	MG	(19:1)	(19:1)		371.32	371.32	12.05	C22 H43 O4	-1.08	0.05	1.76	DR
MG(23:1)+NH4	MG(23:1)+NH4	MG	(23:1)	(23:1)		444.40	444.41	2.73	C26 H54 O4 N1	-1.33	0.00	2.09	DR
MG(23:1)+NH4	MG(23:1)+NH4	MG	(23:1)	(23:1)		444.40	444.40	3.55	C26 H54 O4 N1	-1.48	0.01	2.13	DR
MG(23:1)+Na	MG(23:1)+Na	MG	(23:1)	(23:1)		449.36	449.36	5.32	C26 H50 O4 Na1	-1.37	0.03	1.81	DR
MG(23:1)+NH4	MG(23:1)+NH4	MG	(23:1)	(23:1)		444.40	444.40	9.78	C26 H54 O4 N1	-1.59	0.01	2.16	DR

MG(23:1)+NH4	MG(23:1)+NH4	MG	(23:1)	(23:1)		444.40	444.40	14.11	C26 H54 O4 N1	-1.52	0.02	2.07	DR
MG(23:1)+Na	MG(23:1)+Na	MG	(23:1)	(23:1)		449.36	449.36	15.32	C26 H50 O4 Na1	-1.28	0.02	1.88	DR
MG(28:1)+NH4	MG(28:1)+NH4	MG	(28:1)	(28:1)		514.48	514.48	3.59	C31 H64 O4 N1	1.84	0.02	1.94	UR
MG(28:1)+H	MG(28:1)+H	MG	(28:1)	(28:1)		497.46	497.46	3.72	C31 H61 O4	1.02	0.01	2.03	UR
MG(28:1)+NH4	MG(28:1)+NH4	MG	(28:1)	(28:1)		514.48	514.48	11.81	C31 H64 O4 N1	1.20	0.03	1.96	UR
MG(28:1)+NH4	MG(28:1)+NH4	MG	(28:1)	(28:1)		514.48	514.48	11.99	C31 H64 O4 N1	-2.02	0.00	2.03	DR
MG(29:1)+H	MG(29:1)+H	MG	(29:1)	(29:1)		511.47	511.47	4.07	C32 H63 O4	1.97	0.04	1.53	UR
MG(29:1)+H	MG(29:1)+H	MG	(29:1)	(29:1)		511.47	511.47	9.43	C32 H63 O4	-2.04	0.03	1.88	DR
MG(29:1)+NH4	MG(29:1)+NH4	MG	(29:1)	(29:1)		528.50	528.50	15.49	C32 H66 O4 N1	-2.13	0.03	1.74	DR
MG(32:1)+Na	MG(32:1)+Na	MG	(32:1)	(32:1)		575.50	575.50	12.96	C35 H68 O4 Na1	1.61	0.03	1.93	UR
PA(36:3)+NH4	PA(36:3)+NH4	PA	(36:3)	(36:3)		716.52	716.52	8.33	C39 H75 O8 N1 P1	1.01	0.03	1.83	UR
PA(38:2)+NH4	PA(38:2)+NH4	PA	(38:2)	(38:2)		746.57	746.57	10.21	C41 H81 O8 N1 P1	1.50	0.05	1.80	UR
PA(41:3)+NH4	PA(41:3)+NH4	PA	(41:3)	(41:3)		786.60	786.60	9.73	C44 H85 O8 N1 P1	1.11	0.04	1.95	UR
PA(42:3)+NH4	PA(42:3)+NH4	PA	(42:3)	(42:3)		800.62	800.62	9.93	C45 H87 O8 N1 P1	1.01	0.04	1.77	UR
PC(15:0_16:1)+H	PC(31:1)+H	PC	(15:0_16:1)	(15:0)	(16:1)	718.54	718.54	9.20	C39 H77 O8 N1 P1	-1.37	0.01	2.17	DR
PC(31:2)+H	PC(31:2)+H	PC	(17:0_14:2)	(17:0)	(14:2)	716.52	716.52	8.33	C39 H75 O8 N1 P1	1.02	0.03	1.83	UR
PC(34:2)+Na	PC(34:2)+Na	PC	(34:2)	(34:2)		780.55	780.55	8.37	C42 H80 O8 N1 P1 Na1	-1.58	0.01	2.12	DR
PC(34:2)+Na	PC(34:2)+Na	PC	(34:2)	(34:2)		780.55	780.55	9.28	C42 H80 O8 N1 P1 Na1	-1.92	0.01	1.87	DR
PC(34:2)+H	PC(34:2)+H	PC	(34:2)	(34:2)		758.57	758.57	13.61	C42 H81 O8 N1 P1	-1.84	0.00	2.18	DR
PC(18:2_18:2)+Na	PC(36:4)+Na	PC	(18:2_18:2)	(18:2)	(18:2)	804.55	804.55	8.80	C44 H80 O8 N1 P1 Na1	-1.23	0.01	2.08	DR
PC(16:1_20:3)+H	PC(36:4)+H	PC	(16:1_20:3)	(16:1)	(20:3)	782.57	782.57	13.61	C44 H81 O8 N1 P1	-1.51	0.03	1.94	DR
PC(20:4_22:6)+H	PC(42:10)+H	PC	(20:4_22:6)	(20:4)	(22:6)	854.57	854.57	8.35	C50 H81 O8 N1 P1	2.00	0.05	1.92	UR
PC(42:1e)+Na	PC(42:1e)+Na	PC	(42:1e)	(42:1e)		880.71	880.71	12.55	C50 H100 O7 N1 P1 Na1	2.07	0.00	2.10	UR
PC(28:0_14:2)+H	PC(42:2)+H	PC	(28:0_14:2)	(28:0)	(14:2)	870.69	870.69	11.45	C50 H97 O8 N1 P1	-1.50	0.01	2.00	DR
PE(34:1)+H	PE(34:1)+H	PE	(34:1)	(34:1)		718.54	718.54	9.20	C39 H77 O8 N1 P1	-1.37	0.01	2.17	DR
PE(24:0_10:2)+H	PE(34:2)+H	PE	(24:0_10:2)	(24:0)	(10:2)	716.52	716.52	8.33	C39 H75 O8 N1 P1	1.02	0.03	1.83	UR
PE(14:1_23:0)+Na	PE(37:1)+Na	PE	(14:1_23:0)	(14:1)	(23:0)	782.57	782.57	13.60	C42 H82 O8 N1 P1 Na1	-2.02	0.00	2.17	DR
PE(37:2)+Na	PE(37:2)+Na	PE	(37:2)	(37:2)		780.55	780.55	8.37	C42 H80 O8 N1 P1 Na1	-1.58	0.01	2.12	DR
PE(14:1_23:1)+H	PE(37:2)+H	PE	(14:1_23:1)	(14:1)	(23:1)	758.57	758.57	13.62	C42 H81 O8 N1 P1	-1.62	0.03	1.89	DR

PE(27:0_12:2)+H	PE(39:2)+H	PE	(27:0_12:2)	(27:0)	(12:2)	786.60	786.60	9.73	C44 H85 O8 N1 P1	1.11	0.04	1.95	UR
PE(39:4)+H	PE(39:4)+H	PE	(39:4)	(39:4)		782.57	782.57	12.40	C44 H81 O8 N1 P1	-3.23	0.02	1.85	DR
PE(29:1_10:3)+H	PE(39:4)+H	PE	(29:1_10:3)	(29:1)	(10:3)	782.57	782.57	13.63	C44 H81 O8 N1 P1	-3.24	0.00	2.15	DR
PE(20:1_20:1)+H	PE(40:2)+H	PE	(20:1_20:1)	(20:1)	(20:1)	800.62	800.62	9.93	C45 H87 O8 N1 P1	1.01	0.04	1.77	UR
PE(31:0_12:2)+H	PE(43:2)+H	PE	(31:0_12:2)	(31:0)	(12:2)	842.66	842.66	10.90	C48 H93 O8 N1 P1	-2.14	0.03	2.01	DR
PEt(19:5CHO)+Na	PEt(19:5CHO)+Na	PEt	(19:5CHO)	(19:5CHO)		521.19	521.19	0.65	C24 H35 O9 N0 P1 Na1	-2.81	0.03	1.90	DR
PEt(28:3e)+H	PEt(28:3e)+H	PEt	(28:3e)	(28:3e)		601.42	601.42	5.76	C33 H62 O7 N0 P1	-2.62	0.04	1.54	DR
PEt(28:3e)+H	PEt(28:3e)+H	PEt	(28:3e)	(28:3e)		601.42	601.42	6.23	C33 H62 O7 N0 P1	-3.67	0.01	1.84	DR
PEt(29:4+2O+Ox)+H	PEt(29:4+2O+Ox)+H	PEt	(29:4+2O+Ox)	(29:4+2O+Ox)		673.37	673.37	15.00	C34 H58 O11 N0 P1	-1.39	0.03	1.75	DR
PEt(34:3)+H	PEt(34:3)+H	PEt	(34:3)	(34:3)		699.50	699.50	8.35	C39 H72 O8 N0 P1	1.16	0.03	2.01	UR
PEt(48:0)+Na	PEt(48:0)+Na	PEt	(48:0)	(48:0)		923.74	923.74	13.40	C53 H105 O8 N0 P1 Na1	1.54	0.00	2.19	UR
PEt(50:3)+H	PEt(50:3)+H	PEt	(50:3)	(50:3)		923.75	923.74	13.40	C55 H104 O8 N0 P1	1.41	0.01	2.13	UR
PI(34:1)+NH4	PI(34:1)+NH4	PI	(34:1)	(34:1)		854.58	854.57	8.35	C43 H85 O13 N1 P1	1.92	0.04	1.92	UR
PI(34:1)+H	PI(34:1)+H	PI	(34:1)	(34:1)		837.55	837.55	8.33	C43 H82 O13 N0 P1	2.09	0.03	1.87	UR
PIP(14:1COOH)+NH4	PIP(14:1COOH)+NH4	PIP	(14:1COOH)	(14:1COOH)		684.20	684.20	0.69	C23 H44 O18 N1 P2	-1.23	0.03	2.04	DR
PIP(14:1COOH)+NH4	PIP(14:1COOH)+NH4	PIP	(14:1COOH)	(14:1COOH)		684.20	684.20	4.39	C23 H44 O18 N1 P2	-1.52	0.02	1.69	DR
PIP(34:2e)+H	PIP(34:2e)+H	PIP	(34:2e)	(34:2e)		901.52	901.52	12.94	C43 H83 O15 N0 P2	1.40	0.05	1.79	UR
PMe(29:3e)+H	PMe(29:3e)+H	PMe	(29:3e)	(29:3e)		601.42	601.42	5.76	C33 H62 O7 N0 P1	-2.62	0.04	1.54	DR
PMe(29:3e)+H	PMe(29:3e)+H	PMe	(29:3e)	(29:3e)		601.42	601.42	6.23	C33 H62 O7 N0 P1	-3.67	0.01	1.84	DR
PMe(30:5+3O)+H	PMe(30:5+3O)+H	PMe	(30:5+3O)	(30:5+3O)		673.37	673.37	14.49	C34 H58 O11 N0 P1	-1.43	0.05	1.89	DR
PMe(49:1)+Na	PMe(49:1)+Na	PMe	(49:1)	(49:1)		921.73	921.73	13.88	C53 H103 O8 N0 P1 Na1	1.10	0.01	2.04	UR
SPH(d14:0)+H	SPH(d14:0)+H	SPH	(d14:0)	(d14:0)		246.24	246.24	5.27	C14 H32 O2 N1	-1.00	0.05	1.78	DR
SPH(d16:0)+H	SPH(d16:0)+H	SPH	(d16:0)	(d16:0)		274.27	274.27	0.65	C16 H36 O2 N1	-2.28	0.01	2.16	DR
SPH(d16:0)+H	SPH(d16:0)+H	SPH	(d16:0)	(d16:0)		274.27	274.27	3.23	C16 H36 O2 N1	-1.83	0.05	1.65	DR
SPH(d16:1)+H	SPH(d16:1)+H	SPH	(d16:1)	(d16:1)		272.26	272.26	4.59	C16 H34 O2 N1	-4.60	0.01	2.11	DR
SPH(d18:0)+H	SPH(d18:0)+H	SPH	(d18:0)	(d18:0)		302.31	302.31	9.41	C18 H40 O2 N1	1.55	0.04	1.92	UR
SPH(t18:0)+H	SPH(t18:0)+H	SPH	(t18:0)	(t18:0)		318.30	318.30	0.65	C18 H40 O3 N1	-2.43	0.01	1.83	DR

SPH(t18:0)+H	SPH(t18:0)+H	SPH	(t18:0)	(t18:0)		318.30	318.30	11.94	C18 H40 O3 N1	-1.62	0.02	1.92	DR
SPH(t18:0)+H	SPH(t18:0)+H	SPH	(t18:0)	(t18:0)		318.30	318.30	15.49	C18 H40 O3 N1	1.04	0.00	2.16	UR
SPH(t20:0)+H	SPH(t20:0)+H	SPH	(t20:0)	(t20:0)		346.33	346.33	4.57	C20 H44 O3 N1	-3.31	0.01	2.21	DR
TG(11:0_6:0_6:0)+Na	TG(23:0)+Na	TG	(11:0_6:0_6:0)	(11:0)	(6:0)	479.33	479.33	5.83	C26 H48 O6 Na1	-1.16	0.01	2.13	DR
TG(6:0_6:0_11:1)+NH4	TG(23:1)+NH4	TG	(6:0_6:0_11:1)	(6:0)	(6:0)	472.36	472.36	4.62	C26 H50 O6 N1	-1.50	0.04	1.82	DR
TG(6:0_6:0_11:1)+NH4	TG(23:1)+NH4	TG	(6:0_6:0_11:1)	(6:0)	(6:0)	472.36	472.36	7.32	C26 H50 O6 N1	-2.03	0.00	2.08	DR
TG(26:0)+Na	TG(26:0)+Na	TG	(26:0)	(26:0)		521.38	521.38	4.67	C29 H54 O6 Na1	1.57	0.02	1.85	UR
TG(16:1e_9:0_9:0)+Na	TG(34:1e)+Na	TG	(16:1e_9:0_9:0)	(16:1e)	(9:0)	617.51	617.51	10.86	C37 H70 O5 Na1	1.13	0.05	1.51	UR
TG(20:2e_8:0_8:0)+NH4	TG(36:2e)+NH4	TG	(20:2e_8:0_8:0)	(20:2e)	(8:0)	638.57	638.57	12.79	C39 H76 O5 N1	2.11	0.05	1.98	UR
TG(36:3e)+NH4	TG(36:3e)+NH4	TG	(14:0e_11:0_11:3)	(14:0e)	(11:0)	636.56	636.55	10.48	C39 H74 O5 N1	1.87	0.02	1.88	UR
TG(14:1e_12:0_12:0)+NH4	TG(38:1e)+NH4	TG	(14:1e_12:0_12:0)	(14:1e)	(12:0)	668.62	668.62	11.02	C41 H82 O5 N1	-3.20	0.04	1.89	DR
TG(5:0COOCH3_17:1_17:1)+Na	TG(39:2COOCH3)+Na	TG	(5:0COOCH3_17:1_17:1)	(5:0COOCH3)	(17:1)	743.54	743.54	9.27	C43 H76 O8 Na1	1.78	0.03	1.89	UR
TG(18:2+8O_15:0_15:0)+H	TG(48:2+8O)+H	TG	(18:2+8O_15:0_15:0)	(18:2+8O)	(15:0)	931.67	931.67	10.94	C51 H95 O14	-1.58	0.02	2.08	DR
TG(20:4+Ox_15:0_15:0)+NH4	TG(50:4+Ox)+NH4	TG	(20:4+Ox_15:0_15:0)	(20:4+Ox)	(15:0)	858.72	858.72	11.69	C53 H96 O7 N1	1.74	0.02	2.07	UR
TG(9:0CHO_19:0_22:4)+NH4	TG(50:4CHO)+NH4	TG	(9:0CHO_19:0_22:4)	(9:0CHO)	(19:0)	858.72	858.72	11.69	C53 H96 O7 N1	1.74	0.02	2.07	UR
TG(18:3+O_16:1_16:1)+NH4	TG(50:5+O)+NH4	TG	(18:3+O_16:1_16:1)	(18:3+O)	(16:1)	858.72	858.72	11.69	C53 H96 O7 N1	1.72	0.02	2.08	UR
TG(51:0)+Na	TG(51:0)+Na	TG	(51:0)	(51:0)		871.77	871.77	8.37	C54 H104 O6 Na1	-1.76	0.04	1.78	DR
TG(28:0_12:1_12:1)+NH4	TG(52:2)+NH4	TG	(28:0_12:1_12:1)	(28:0)	(12:1)	876.80	876.80	9.79	C55 H106 O6 N1	-5.12	0.01	2.24	DR
TG(30:1_6:0_18:1)+NH4	TG(54:2)+NH4	TG	(30:1_6:0_18:1)	(30:1)	(6:0)	904.83	904.83	10.87	C57 H110 O6 N1	2.18	0.03	2.06	UR
TG(30:0_10:0_14:3)+NH4	TG(54:3)+NH4	TG	(30:0_10:0_14:3)	(30:0)	(10:0)	902.82	902.82	8.09	C57 H108 O6 N1	2.19	0.01	1.83	UR
TG(20:2_14:1_20:3)+NH4	TG(54:6)+NH4	TG	(20:2_14:1_20:3)	(20:2)	(14:1)	896.77	896.77	10.90	C57 H102 O6 N1	-1.49	0.01	2.12	DR
TG(29:0_10:1_16:1)+NH4	TG(55:2)+NH4	TG	(29:0_10:1_16:1)	(29:0)	(10:1)	918.85	918.85	11.50	C58 H112 O6 N1	1.58	0.01	1.81	UR
TG(57:12)+Na	TG(57:12)+Na	TG	(57:12)	(57:12)		931.68	931.68	11.03	C60 H92 O6 Na1	-2.07	0.04	1.62	DR
TG(57:9e)+Na	TG(57:9e)+Na	TG	(57:9e)	(57:9e)		923.75	923.74	13.40	C60 H100 O5 Na1	1.51	0.00	2.19	UR
TG(30:0_10:1_18:4)+NH4	TG(58:5)+NH4	TG	(30:0_10:1_18:4)	(30:0)	(10:1)	954.85	954.85	12.73	C61 H112 O6 N1	-1.23	0.00	2.10	DR
TG(30:1_9:0_20:2)+NH4	TG(59:3)+NH4	TG	(30:1_9:0_20:2)	(30:1)	(9:0)	972.90	972.89	13.23	C62 H118 O6 N1	1.24	0.01	2.14	UR
TG(59:3)+NH4	TG(59:3)+NH4	TG	(59:3)	(59:3)		972.90	972.89	13.36	C62 H118 O6 N1	1.62	0.05	1.50	UR
TG(27:0_12:3_20:4)+NH4	TG(59:7)+NH4	TG	(27:0_12:3_20:4)	(27:0)	(12:3)	964.83	964.83	12.30	C62 H110 O6 N1	-1.67	0.02	1.72	DR
TG(30:1_10:2_20:1)+NH4	TG(60:4)+NH4	TG	(30:1_10:2_20:1)	(30:1)	(10:2)	984.90	984.89	12.95	C63 H118 O6 N1	-3.34	0.00	2.22	DR
TG(29:0_14:4_17:0)+NH4	TG(60:4)+NH4	TG	(29:0_14:4_17:0)	(29:0)	(14:4)	984.90	984.90	13.09	C63 H118 O6 N1	4.32	0.00	2.10	UR
TG(60:5)+NH4	TG(60:5)+NH4	TG	(60:5)	(60:5)		982.88	982.88	11.86	C63 H116 O6 N1	1.82	0.04	1.84	UR

TG(28:1_10:0_22:6)+NH4	TG(60:7)+NH4	TG	(28:1_10:0_22:6)	(28:1)	(10:0)	978.85	978.85	12.46	C63 H112 O6 N1	-1.01	0.04	1.80	DR
TG(67:6+6O)+NH4	TG(67:6+6O)+NH4	TG	(67:6+6O)	(67:6+6O)		1174.94	1174.94	10.39	C70 H128 O12 N1	2.32	0.00	2.15	UR
WE(14:0)+NH4	WE(14:0)+NH4	WE	(14:0)	(14:0)		246.24	246.24	2.75	H32 C14 O2 N1	1.61	0.03	2.04	UR
WE(16:0)+NH4	WE(16:0)+NH4	WE	(16:0)	(16:0)		274.27	274.27	0.90	H36 C16 O2 N1	-1.05	0.04	1.94	DR
WE(16:0)+NH4	WE(16:0)+NH4	WE	(16:0)	(16:0)		274.27	274.27	3.23	H36 C16 O2 N1	-1.83	0.05	1.65	DR
WE(16:0)+NH4	WE(16:0)+NH4	WE	(16:0)	(16:0)		274.27	274.27	7.31	H36 C16 O2 N1	-1.85	0.01	1.55	DR
WE(16:0)+NH4	WE(16:0)+NH4	WE	(16:0)	(16:0)		274.27	274.27	11.92	H36 C16 O2 N1	-1.59	0.03	1.71	DR
WE(16:1)+NH4	WE(16:1)+NH4	WE	(16:1)	(16:1)		272.26	272.26	3.07	H34 C16 O2 N1	-2.37	0.00	2.21	DR
WE(17:2)+H	WE(17:2)+H	WE	(17:2)	(17:2)		267.23	267.23	2.94	H31 C17 O2	1.72	0.04	2.01	UR
WE(18:0)+NH4	WE(18:0)+NH4	WE	(18:0)	(18:0)		302.31	302.31	9.41	H40 C18 O2 N1	1.43	0.05	1.84	UR
WE(7:0_14:2)+H	WE(21:2)+H	WE	(7:0_14:2)	(7:0)	(14:2)	323.29	323.29	12.77	H39 C21 O2	-1.42	0.03	1.84	DR
WE(22:1)+NH4	WE(22:1)+NH4	WE	(22:1)	(22:1)		356.35	356.35	1.53	H46 C22 O2 N1	2.01	0.01	2.14	UR
WE(32:0+Ox)+NH4	WE(32:0+Ox)+NH4	WE	(32:0+Ox)	(32:0+Ox)		512.50	512.50	10.25	H66 C32 O3 N1	1.25	0.01	2.00	UR
dMePE(22:0_10:2)+H	dMePE(32:2)+H	dMePE	(22:0_10:2)	(22:0)	(10:2)	716.52	716.52	8.33	C39 H75 O8 N1 P1	1.01	0.03	1.83	UR
dMePE(34:3)+H	dMePE(34:3)+H	dMePE	(34:3)	(34:3)		742.54	742.54	9.27	C41 H77 O8 N1 P1	1.02	0.04	2.00	UR
dMePE(43:2)+H	dMePE(43:2)+H	dMePE	(43:2)	(43:2)		870.69	870.69	11.45	C50 H97 O8 N1 P1	-1.50	0.01	2.00	DR

DR=down regulation, UR=upregulation, VIP=Variable importance projection
